# Antennal Transcriptome Screening and Identification of Chemosensory Proteins in the Double-Spine European Spruce Bark Beetle, *Ips duplicatus* (Coleoptera: Scolytinae)

**DOI:** 10.3390/ijms25179513

**Published:** 2024-09-01

**Authors:** Jibin Johny, Ewald Große-Wilde, Blanka Kalinová, Amit Roy

**Affiliations:** Faculty of Forestry and Wood Sciences, Czech University of Life Sciences Prague, Kamýcká 129, 16500 Prague, Czech Republic; jibinmjo@gmail.com (J.J.);

**Keywords:** antennal transcriptome, bark beetles, Coleoptera, chemosensory proteins, *Ips duplicatus*, olfaction, RNAseq, tetramer OBP, orthologs

## Abstract

The northern bark beetle, *Ips duplicatus*, is an emerging economic pest, reportedly infesting various species of spruce (*Picea* spp.), pine (*Pinus* spp.), and larch (*Larix* spp.) in Central Europe. Recent climate changes and inconsistent forest management practices have led to the rapid spread of this species, leaving the current monitoring strategies inefficient. As understanding the molecular components of pheromone detection is key to developing novel control strategies, we generated antennal transcriptomes from males and females of this species and annotated the chemosensory proteins. We identified putative candidates for 69 odorant receptors (ORs), 50 ionotropic receptors (IRs), 25 gustatory receptors (GRs), 27 odorant-binding proteins (OBPs), including a tetramer-OBP, 9 chemosensory proteins (CSPs), and 6 sensory neuron membrane proteins (SNMPs). However, no sex-specific chemosensory genes were detected. The phylogenetic analysis revealed conserved orthology in bark beetle chemosensory proteins, especially with a major forest pest and co-habitant, *Ips typographus*. Recent large-scale functional studies in *I. typographus* chemoreceptors add greater significance to the orthologous sequences reported here. Nevertheless, identifying chemosensory genes in *I. duplicatus* is valuable to understanding the chemosensory system and its evolution in bark beetles (Coleoptera) and, generally, insects.

## 1. Introduction

The northern bark beetle, *Ips duplicatus* (Sahlberg, 1836) (Coleoptera, Curculionidae, Scolytinae), also known as the double-spined bark beetle, is native to Fennoscandia, Siberia, and East Asia and has become an emerging economic pest in recent years [1]. Their primary host trees are various species of spruce (*Picea* spp.), mainly Norway spruce (*Picea abies* (L.) Karst, but they have also been observed on pine (*Pinus* spp.) and larch (*Larix* spp.) in Central Europe [2]. *I. duplicatus* shares an analogous ecology to the notorious and widespread European spruce bark beetle, *Ips typographus* (Linnaeus, 1758) [1]. However, *I. duplicatus* has a shorter development time and predominantly attacks the upper parts of shaded trees inside the stand, making their early detection a challenging task [3,4]. Additionally, they carry specific fungal plant pathogens for their Norway spruce colonization [5]. Currently, *I. duplicatus* infestations have been reported in 80% of spruce trees from sanitary felling in the northeastern parts of the Czech Republic [4,6]. The first outbreak of *I. duplicatus* in the Czech Republic was reported in the 1990s [6], and recent climate changes, physiological tree stress, and inconsistent forest protection have favored the rapid spread of this secondary pest to become a serious primary pest [2,4,5,6,7]. Recently, the bark beetle infestations have doubled the spruce mortality in Central Europe, reaching nearly 20 million m^3^ in the Czech Republic [3,8].

With up to three generations of *I. duplicatus* per season, early monitoring is key to successful control and management of this species [9]. As olfaction plays a crucial role in bark beetle communications and host colonization, most of the *I. duplicatus* management strategies rely on pheromonal traps baited with specific synthetic lures, such as ID Ecolure; however, there is observed seasonality in captures due to overwintering generations [9,10]. The key *I. duplicatus* aggregation pheromone components are ipsdienol (ID) (Bakke 1975) and *E*-myrcenol (EM) [11] in a ratio ranging from 1:1 to 1:9 for ID and EM across the populations [12]. *I. duplicatus* has also been found to respond to aggregation pheromone components of co-habitant *I. typographus*, viz., 2-methyl-3-buten-2-ol and *cis*-verbenol [12,13]. Additionally, amitinol has also been identified as an aggregation pheromone component from the Mongolian *I. duplicatus* population [14]. Moreover, host monoterpenes like (−)-α-pinene together with (+)-limonene have a synergic effect on the attractivity of *I. duplicatus*’ aggregation pheromone [13,15]. However, such lure-based traps were ineffective due to the biology of these beetles [16]. Although reverse chemical ecology approaches are promising, unlike in *I. typographus*, the peripheral olfactory detection in *I. duplicatus* at cellular and molecular levels has been understudied until now.

Peripheral olfactory detection in most insects occurs at the olfactory sensilla present predominantly in the insect antennae [17,18]. At the cellular level, these sensilla house olfactory sensory neurons (OSNs) that carry receptor proteins that bind to specific odorants [17,18]. While the pheromones and other volatile compounds are detected by odorant receptors (ORs) and ionotropic receptors (IRs) expressed in two distinct sensillum types, the taste-related and environmental chemosensory signals are detected by gustatory receptors (GRs) [19,20,21,22,23,24]. Insect ORs are seven-transmembrane proteins originating from GRs [25,26] with an inverted topology compared to classical G-protein-coupled receptors (GPCRs) [21]. ORs function as a critical subunit in the heteromeric ion channels formed with ubiquitous OR coreceptor (ORCo) [27]. The ratio of OR and ORCo in such heteromeric complexes has been recently identified as 1:3 [28,29]. However, monomeric channels without ORCo have also been reported [27]. The ligand binding activates the receptor, allowing the flow of ions through the membrane and generating an action potential that is transferred to the brain [21,27]. The OR repertoire ranges from 62 ORs in *Drosophila melanogaster* [19,21] to 375 ORs in ants [30]. In *I. typographus*, 73 ORs have been identified based on genome and transcriptome approaches [31]; however, such information is lacking from other *Ips* spp.

IRs are transmembrane proteins with an ancient protostomic origin and are distantly related to a variant of ionotropic glutamate receptors, iGluRs [23,32]. They are involved in broader functions for detecting environmental and intercellular chemical signals and are co-expressed with receptors IR8a or IR25a [23,32]. iGluRs are generally classified by their primary agonists, viz., α-amino-3-hydroxy-5-methyl-4-isoxazolepropionic acid (AMPA), kainate, and N-methyl-D-aspartate (NMDA), whereas the IRs are classified as antennal IRs and divergent IRs [23,32]. GRs are seven-transmembrane proteins that are evolutionarily related to ORs and have an inverted topology compared to GPCRs [33,34]. GRs are involved primarily in detecting tastants, such as sugar, bitterness, and CO_2_, and are primarily expressed in taste-sensing neurons [35]. GRs are also expressed in OSNs that detect pheromone compounds in *D. melanogaster* [36].

Apart from receptor proteins, non-receptor proteins, like odorant-binding proteins (OBPs), chemosensory proteins (CSPs), and sensory neuron membrane proteins (SNMPs), are also involved in peripheral olfactory detection in insects [17,37,38]. OBPs are small proteins (~120–200 amino acids in length) found abundantly in sensillar lymph and are mainly involved in the transport of odorant to the receptor [37,39,40]. They are classified based on function and structural features like conserved cysteine residues [41]. CSPs are also small globular proteins found in the olfactory sensillar lymph. Their exact function is unclear, as they could either transport odorant to the membrane receptor or protect the odor from degradation [40,42]. SNMPs are a CD36 (cluster of differentiation 36) family of transmembrane proteins with diverse functions [22,43]. Two SNMP classes have been reported in insects: SNMP1, expressed in OSNs and involved in lipid-derived pheromone detection [38,44], and SNMP2, mainly expressed in OSN-supporting cells with possible involvement in the pheromone clearing process [45,46]. The role of SNMP1 proteins in insect pheromone detection has been explored recently by their ectodomain analysis [38], cellular expression [46], and in vivo gene-silencing methods [47].

Genomic and transcriptomic approaches have been instrumental in identifying and characterizing key vital chemosensory proteins in insects, especially in Coleoptera [31,48,49,50,51,52]. In bark beetles, OR deorphanization is in progress, mainly from *I. typographus* and *Dendroctonus ponderosae* [53]. The conserved response patterns observed between the OR orthologs in these species renders the search for orthologs in another *Ips* species more interesting [53]. Here, we report the antennal transcriptome-based identification of chemosensory proteins from *I. duplicatus*, one of the emerging economic pests of forests, and we analyze the conserved orthology in bark beetle chemosensory genes. With limited control measures available against these beetles, our findings have enormous implications for developing olfaction-based management strategies.

## 2. Results

### 2.1. De Novo Antennal Transcriptome Sequencing and Assembly

We generated four *I. duplicatus* antennal transcriptomes using Illumina paired-end sequencing as IDUP_AF1: *I. duplicatus* female 1, IDUP_AF2: *I. duplicatus* female 2, IDUP_AM1: *I. duplicatus* male 1, and IDUP_AM2: *I. duplicatus* male 2. The IDUP_AM1 generated 20.37 million paired reads, assembled into 105,416 transcripts, with 88.56% paired reads mapped to the preliminary *I. duplicatus* genome (unpublished). IDUP_AM2 generated 23.82 million paired reads, assembled into 121,285 transcripts, with 88.30% reads mapped to the genome. IDUP_AF1 generated 19.74 million paired reads, assembled into 91,822 transcripts, with 87.97% of the reads mapped to the genome. IDUP_AF2 generated 20.35 million paired reads, assembled into 98,264 transcripts, with 88.63% reads mapped [54] to the preliminary genome. All the individual transcriptome assemblies showed 93.7–95.1% completeness, based on a BUSCO v5.3.2 assessment using *insecta10* as a reference [55]. A combined assembly of all the reads from all four samples generated 204,588 transcripts with a BUSCO v5.3.2 completeness of 99.71% against the *insecta10* dataset. The assembled transcripts were further refined by clustering to a final count of 63,835 transcripts. The assembly statistics are provided in Table 1.

### 2.2. Odorant Receptors in I. duplicatus

**Figure 1:**
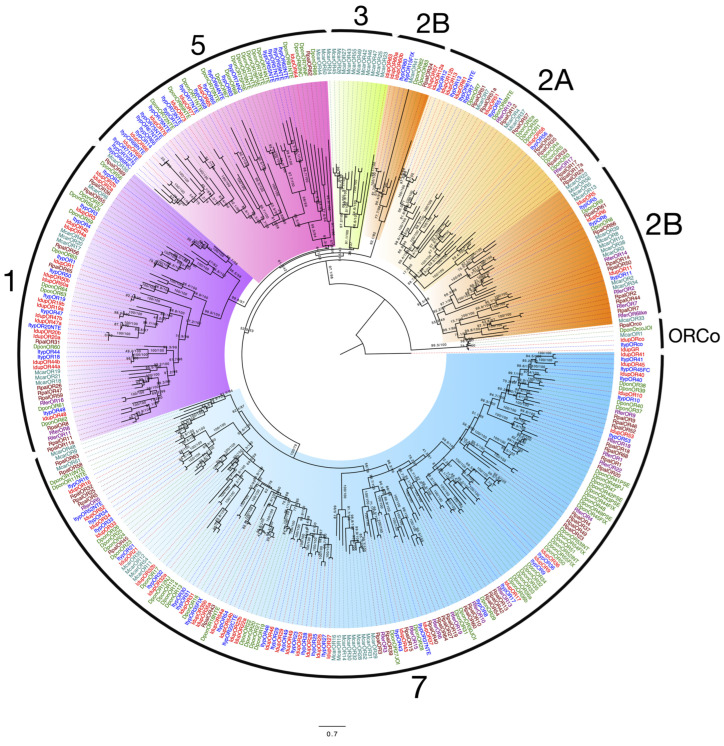
Maximum likelihood phylogeny of *I. duplicatus* ORs. The phylogeny was reconstructed using predicted OR sequences from *I. duplicatus* and selected coleopteran species. The tree was rooted with IdupGR, with ORCo at the basal node. Each coleopteran OR subfamily is highlighted with separate colors and marked with respective numbers. ORs from *I. duplicatus* (Idup) are colored in red, and other species were colored as follows: *I. typographus* (blue), *M. caryae* (pale green), *D. ponderosae* (green), *R. palmarum* (brown), and *R. ferrugineus* (violet). The branch labels indicate SH-like approximate likelihood ratio test (SH-aLRT) value/bootstrap value. The scale represents amino acid substitutions per site.

Our manual annotations of *I. duplicatus* transcriptomes using separate and combined assemblies revealed 69 IdupORs, including the co-receptor, ORCo (Supplementary Information S1). With predicted typical seven-transmembrane regions, we report 50 IdupORs as full-length and 19 as partial sequences (Supplementary Information S2). We found no sex-specific ORs in our analysis. All the IdupORs were named based on their orthology to ItypORs [31,53]. The ML phylogeny reconstructed using JTT+F+R9 based on the Bayesian information criterion (BIC) score revealed the seven reported coleopteran OR subfamilies [48,53,56]. The phylogeny was rooted with IdupGR1 (Figure 1).

Next to the basal lineage ORCo, the most divergent OR subfamily 2 had 12 members equally representing 2a and 2b subfamilies (Figure 1). The OR subfamily 3 (green) was reported only in *M. caryae* [57], and no IdupORs were grouped into this clade. A partial sequence of *IdupOR63* was found within the subfamily 3 clade, however, without enough bootstrap support (<50%) (Figure 1). Seven IdupORs were grouped into subfamily 5. Interestingly, we found bark-beetle-specific OR expansions in this subfamily. Seventeen IdupORs were grouped as OR subfamily 1; however, seven existed as variants (Figure 1). Thirty-one IdupORs were included in the subfamily 7, the largest in coleopteran OR subfamilies (Figure 1). We also found three members in this subfamily with variants, but not isoforms, named with the suffix ‘a’ and ‘b’ (Figure 1). Conserved orthology was identified between ItypORs and IdupORs throughout the phylogeny, including a 1:1 orthology in a well-characterized *Ips* spp. specific OR clade in subfamily 7. The receptors in this clade include *I. typographus* pheromone receptors [31,52].

### 2.3. Ionotropic Receptors and iGulR Family Receptors in I. duplicatus

**Figure 2:**
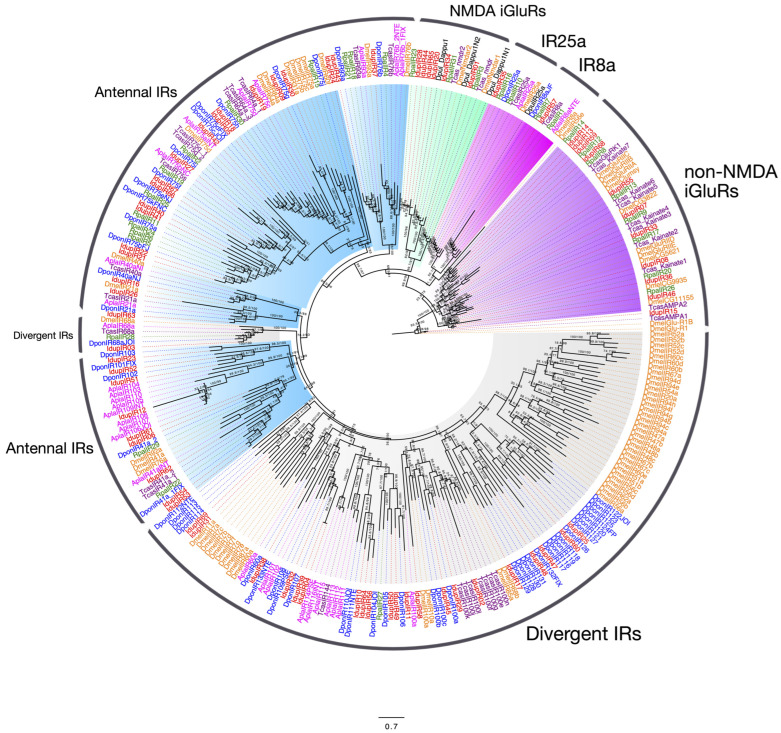
Maximum likelihood phylogeny of *I. duplicatus* IRs. The tree was reconstructed using predicted IR sequences from *I. duplicatus* and selected coleopterans and *D. melanogaster*. The tree was rooted with a non-NMDA iGluR, DmelGlu-R1. All the iGluR sub-families are color-coded and marked on the taxa labels. The IdupIRs were marked in red, and the other species were colored as follows: *D. ponderosae* (blue), *A. planipennis* (magenta), *T. castaneum* (violet), *D. melanogaster* (orange), *R. palmarum* (green), and *Daphnia pulex* (black). The branch labels indicate SH-aLRT value/bootstrap value. The scale represents amino acid substitutions per site.

Our annotations using insect ionotropic glutamate receptor family proteins (iGluRs) identified 69 transcripts in *I. duplicatus* antennae as iGluRs family members. Fifty IRs were identified with no sex-specific full-length transcripts. As iGluRs are further classified based on sequence homology, we reconstructed a maximum likelihood phylogeny using all the well-reported classes of iGluRs [23,32]. The ML phylogeny was constructed based on the amino acid substitution model LG+F+R8 identified based on the BIC score and rooted with non-NMDA iGluRs from *D. melanogaster*. Six groups of iGluRs were identified from the phylogeny as non-NMDA iGluRs, IR8a, IR25a, NMDA-receptors, antennal IRs, and divergent IRs. Eleven receptors were found to be non-NMDA, including kainite receptors (Figure 2). We found one representative for the IR co-expressing receptors IR8a and IR25a (Figure 2). Six iGluRs were found to be NMDA-receptors (Figure 2). Within the remaining iGluRs, 28 IRs were classified as potential antennal IRs, and the remaining 22 were classified as divergent IRs based on the phylogeny (Figure 2). The number of IRs identified was similar to the annotations reported in *D. ponderosae* [58]. The IR phylogeny revealed the divergence of antennal and divergent IRs in Coleoptera and Diptera. Interestingly, bark-beetle-specific expansions were identified in divergent IRs, similar to the species-specific expansion found in *D. melanogaster* divergent IRs.

### 2.4. Gustatory Receptors in I. duplicatus

**Figure 3:**
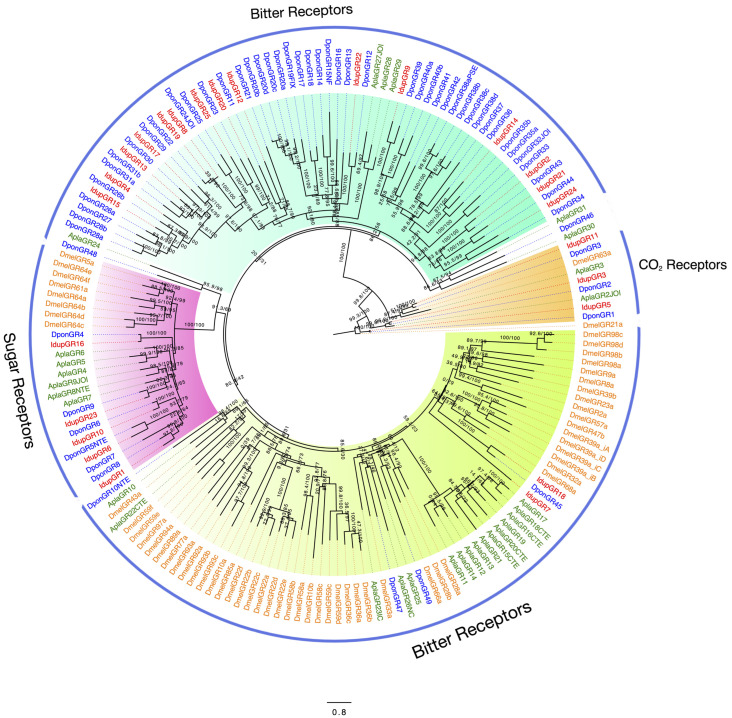
Maximum likelihood phylogeny of *I. duplicatus* GRs. The tree was reconstructed using predicted GR sequences from *I. duplicatus* and selected coleopterans and *D. melanogaster* GRs. The tree was rooted with a CO_2_-sensing *DmelGR21a.* GR clades are color-coded according to the characterized *D. melanogaster* sequences (orange). The IdupGRs were marked in red, and the other species were colored as follows: *D. ponderosae* (blue) and *A. planipennis* (green). The branch labels indicate SH-aLRT value/bootstrap value. The scale represents amino acid substitutions per site.

Our antennal transcriptome analysis revealed 25 GRs in *I. duplicatus*, with none being sex-specific. The ML phylogeny revealed different classes of GRs based on their similarities with well-characterized GRs from *D. melanogaster* [36,59]. The phylogenetic tree was constructed based on the LG+F+R6 amino acid substitution model identified based on the BIC score and was rooted with DmelGR21a. Both DmelGR21a and DmelGR63a are known for detecting CO_2_ in *D. melanogaster* [36]. We found three IdupGR candidates in the clade of GRs sensing CO_2_ with 1:1 orthology to DponGRs (Figure 3: orange clade). The two main GR classes identified were sugar- and bitter-sensing receptors based on the characterized DmelGRs [60,61,62]. Five candidate IdupGRs were identified within the clade of sugar-sensing receptors with orthology to DponGRs (Figure 3: violet clade), whereas in the bitter-tasting receptor clades, species-specific expansions were detected (Figure 3). Similarly, coleopteran-specific GR expansions were also found in the phylogeny with 15 IdupGRs and a similar number of DpondGRs (Figure 3).

A large clade of GRs found between the CO_2_- and sugar-sensing receptors were classified as potential bitter-sensing receptors. We also found two IdupORs sharing similarities with *D. melanogaster* GRs known for courtship behavior [36,63,64]. Interestingly, conserved orthology was identified between IdupGRs and DponGR but not with AplaGRs, indicating bark-beetle-specific GR expansions. Such bark-beetle-specific expansions were observed in the sugar-sensing receptors and within the large clade of bitter receptors (Figure 3).

### 2.5. Odorant-Binding Proteins in I. duplicatus

**Figure 4:**
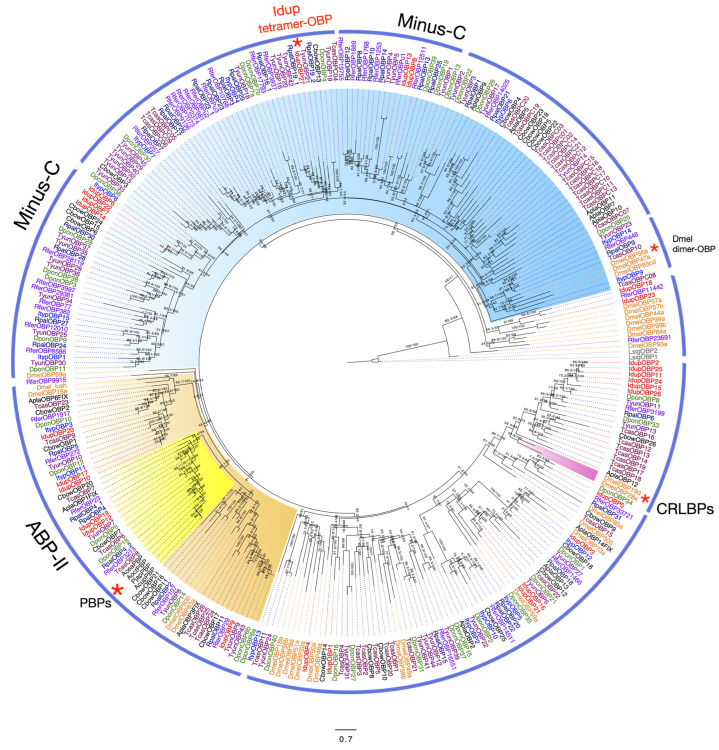
Maximum likelihood phylogeny of *I. duplicatus* OBPs. The tree was reconstructed using predicted OBP sequences from *I. duplicatus* and other selected insect orders. *L. y-signata* OBPs were used as an outgroup. The IdupOBPs are colored in red on the taxa labels, and other OBPs were colored as follows: *I. typographus* (blue), *D. ponderosae* (green), *A. planipennis* (black), *T. castaneum* (maroon), *D. melanogaster* (orange), *C. bowringi* (black), *T. yunnanensis* (violet), *R. ferrugineus* (light violet), and *R. palmarum* (dark blue). Reported pheromone-binding proteins from *Popilio japonica, A. corpulenta*, *A. cuprea*, and *A. octiescostata* were labeled and marked with red (*) within the ABP-II clade. Other OBP subfamilies are marked on the taxa labels based on a sequence analysis; however, the sequence homology is less between the groups. CRLBPs and a dimer-OBP from *D. melanogaster* are labeled and marked with red (*). The tetramer-OBP from *I. duplicatus* is marked with red text and *. The branch labels indicate SH-aLRT value/bootstrap value. The scale bar represents amino acid substitutions per site.

We identified 27 OBPs in *I. duplicatus* antennae, including 20 full-length OBPs. Signal peptides were identified from 18 IdupOBPs (Supplementary Information S3). OBPs are generally classified based on function, antennal expression, and structural features [65]. As 27 OBPs were annotated from the antennal transcriptomes and shared less homology between orthologs, we used sequence characteristics to classify them as Classic OBPs, Minus-C OBPs, Plus-C OBPs, and atypical OBPs [41]. However, no sex-specific OBPs were found in our antennal transcriptomes. We identified 11 Classic-OBPs (six conserved cysteine residues), viz., IdupOBP1, IdupOB10, IdupOBP12, IdupOBP17, IdupOBP24, IdupOBP25, IdupOBP25, IdupOBP26, IdupOBP3, IdupOBP5, and IdupOBP9 in *I. duplicatus* antennae (Figure 4, Supplementary Information S4). The five Minus-C OBPs identified were IdupOBP14, IdupOBP18, IdupOBP6, IdupOBP7, and IdupOBP8. We found four IdupOBPs with one additional cysteine at the C-terminal region and classified them as atypical OBPs.

**Figure 5:**
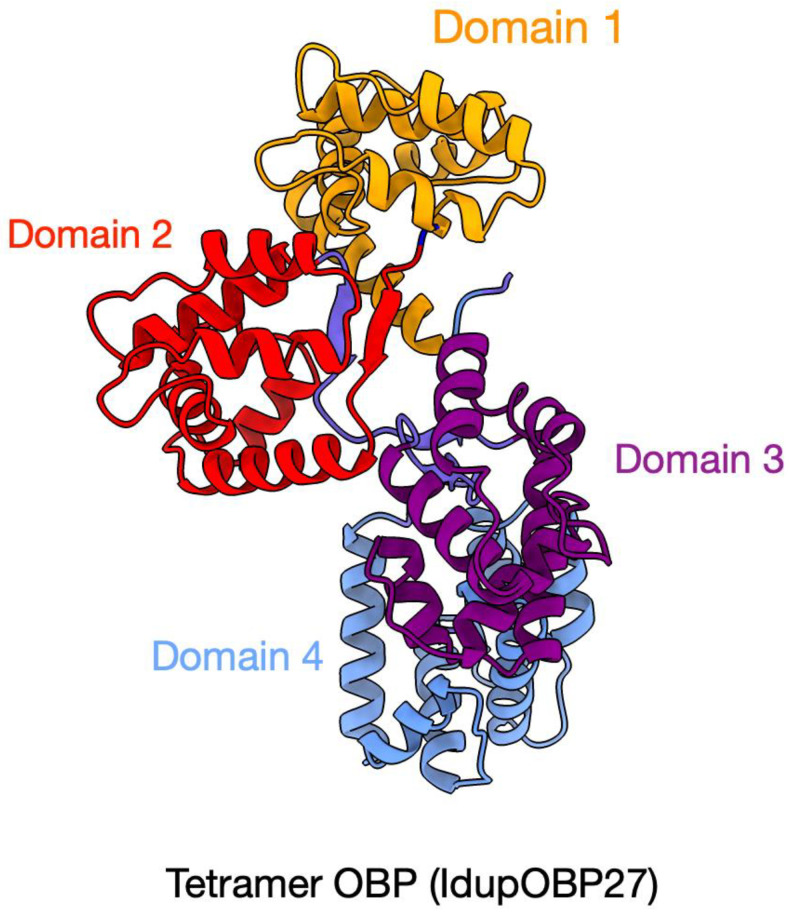
Predicted 3D structure of the tetramer-OBP, IdupOBP27. The structure was predicted using AlphaFold3, excluding the signal peptide (predicted by SignalP v6). Four GOBP/PBP-binding domains are colored and labeled (1–4) from N-terminal to C-terminal.

Interestingly, IdupOBP27 was found to have 12 cysteine residues in the C-terminal region; however, it showed no orthology to the dimer OBP DmelOBP83cd reported from *D. melanogaster* [65,66] (Figure 4). Further analysis revealed its sequence similarity to OBPs from *Dendroctonus adjunctus* (ACN: QKV34985.1) and *D. ponderosae* (ACN: AGI05167.1), and the presence of four structural domains concluded IdupOBP27 as a tetramer-OBP (Figure 5). The predicted structure of this protein is provided in Figure 5. The maximum likelihood IdupOBP phylogeny was reconstructed using LG+R4 as an amino acid substitution model based on the BIC score. The phylogeny provided more resolution to the classification, as OBPs show less sequence similarity across insect orders [67]. Based on the functions, OBPs are classified into General OBPs and antennal-binding proteins [41], which generally include pheromone-binding proteins (PBPs). PBPs are key OBPs specifically involved in the binding and transport of pheromones to the receptor and are well-characterized in many insect orders [68,69]. The antennal OBPs are highlighted in orange in the phylogeny, in which a clade of PBPs is highlighted in yellow (Figure 4) based on the sequences from the characterized PBPs [70]. The Minus-C OBP clade is highlighted in blue, whereas the remaining OBPs are considered general OBPs. The chemical-sense-related lipophilic-ligand-binding protein (CRLBP) [65] clade was highlighted in pink; however, no orthologs were found in *I. duplicatus*.

### 2.6. Chemosensory Proteins (CSPs) in I. duplicatus

**Figure 6:**
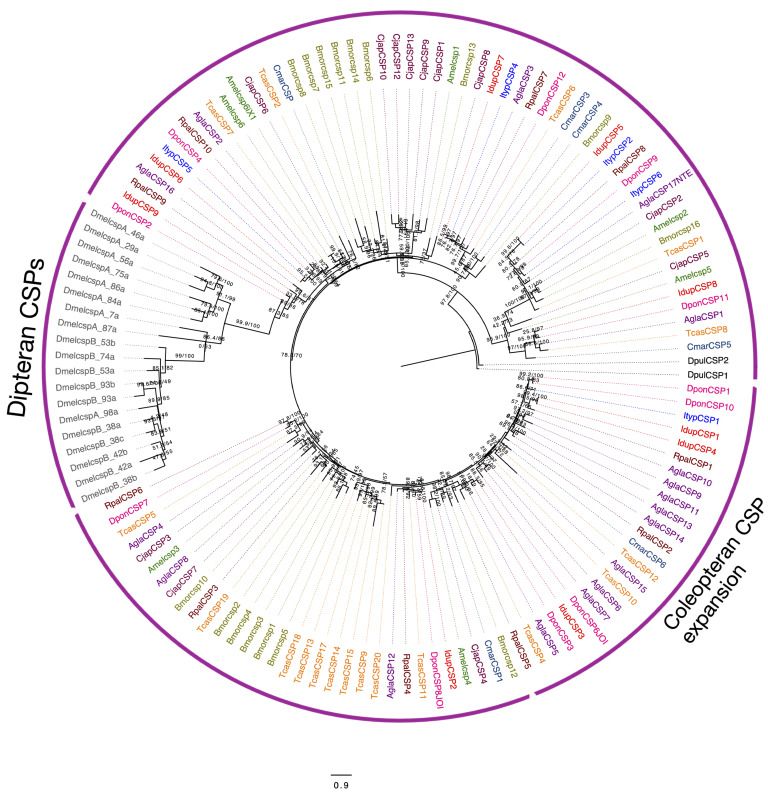
Maximum likelihood phylogeny of *I. duplicatus* CSPs. The tree was reconstructed using predicted CSP sequences from *I. duplicatus* and CSPs reported from other insect orders. The IdupCSPs are colored in red and other OBPs were colored as follows: *T. castaneum* (orange), *D. ponderosae* (pink) *R. palmarum* (brown), *A. glabripennis* (magenta), *Bombyx mori* (olive green), *C. japonicus* (maroon), *C. marinus* (dark blue), and *Apis mellifera* (green). *D. pulex* (black) CSPs were used as an outgroup. The Diperan and coleopteran-specific CSP expansions are marked on the taxa labels. The branch labels indicate SH-aLRT value/bootstrap value. The scale represents amino acid substitutions per site.

Chemosensory proteins with four conserved cysteine residues are known to bind odorants and pheromones in insects [71]. We identified nine chemosensory genes in *I. duplicatus* in our antennal transcriptome screening. Eight of them were found to have four conserved cysteine residues, except IdupCSP6 (Supplementary Information S5). Interestingly, IdupCSP7 was found to have a long C-terminal chain (Supplementary Information S5). However, none of the IdupCSP transcripts were sex-specific. The maximum likelihood phylogeny of CSPs reconstructed based on the LG+R4 amino acid substitution model (based on the BIC score) allowed further comparisons between the species (Figure 6). Only six CSPs were reported from *I. typographus* [53], and we found orthologs of five ItypCSPs, except for ItypCSP6. However, 1:1 orthology was found in *D. ponderosae* CSPs, as 11 CSPs were reported from that species [53]. Unlike other protein families studied, bark-beetle-specific expansions were not found in the CSPs (Figure 6). However, species-specific expansion was observed in the *D. melanogaster* CSPs (Figure 6).

### 2.7. Sensory Neuron Membrane Proteins (SNMPs) in I. duplicatus

**Figure 7:**
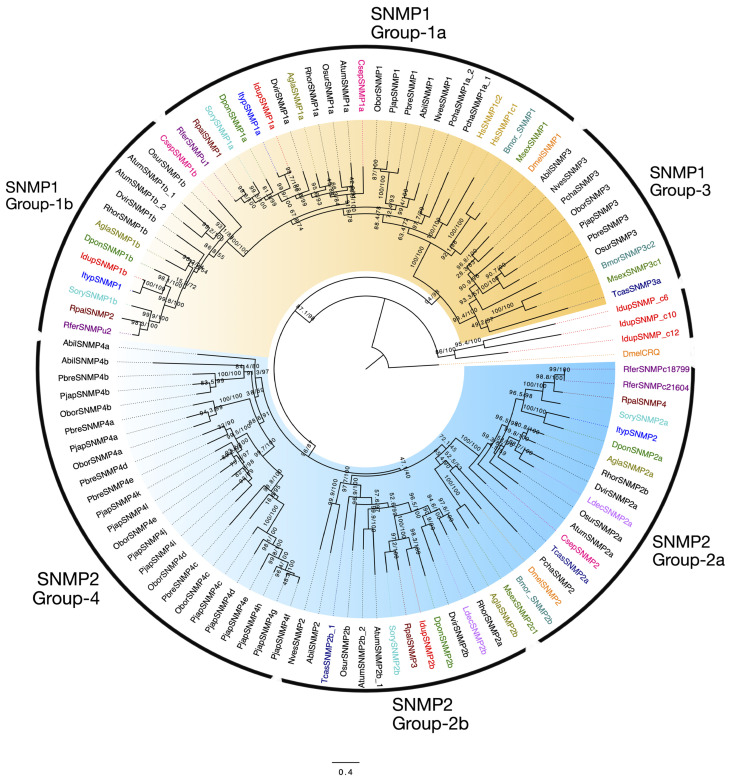
Maximum likelihood phylogeny of *I. duplicatus* SNMPs. The tree was reconstructed using predicted SNMP sequences from *I. duplicatus* and selected SNMPs from other insect orders. The SNMP1 and SNMP2 classes were highlighted in orange and blue, respectively. On the taxa, the IdupSNMPs were colored red, and other OBPs were colored as follows: *I. typographus* (blue), *D. ponderosae* (green), *R. palmarum* (brown), *A. glabripennis* (olive green), *D. melanogaster* (orange), *T. castaneum* (dark blue), *Aethina tumida* (black), *Manduca sexta* (dark olive), *R. ferrugineus* (magenta), and *S. oryzae* (light blue). SNMPs from Scarabaeidae beetles were not color coded. All the SNMP subgroups are marked outside the taxon labels. A non-SNMP protein, croquemort (crq), from *D. melanogaster* was used as an outgroup. The branch labels indicate SH-aLRT value/bootstrap value. The scale represents amino acid substitutions per site.

We identified six SNMPs in *I. duplicatus*, representing contigs in both male and female transcriptomes. Using a maximum likelihood phylogeny reconstructed based on the LG+R4 amino acid substitution model (BIC score), we classified three candidates as SNMP classes 1 (Figure 7: highlighted orange) and 2 (Figure 7: highlighted blue). Two SNMP candidates, IdupSNMP1a and IdupSNMP1b, were grouped as SNMP1a and SNMP1b classes of proteins, respectively (Figure 7). The one SNMP2 protein identified in *I. duplicatus* belonged to the 2b group and was named IdupSNMP2b (Figure 7). No IdupSNMP1 candidates were found in Group 3, and none in the IdupSNMP2 belonged to Group 4. Three candidates, IdupSNMPc6, IdupSNMPc10 and IdupSNMPc12, were identified as SNMPs with low blast identity scores, respectively, to *Anthonomus grandis* (ACN: AWF93834.1), *Drosophila navojoa* (ACN: XP_017969087.1), and *Meteorus pulchricornis* (ACN: QCS38482.1). These three SNMPs were highly divergent in the phylogeny (Figure 7) but were unrelated to the outgroup CD36 croquemort protein. They shared sequence similarity with scavenger receptor class B proteins. The ungrouped SNMPs were named with the suffix ‘c’, followed by a contig number. The SNMP1 candidates shared orthology with IdupSNMPs and ItypSNMPs, whereas the SNMP2b protein was an ortholog of DponSNMP2b. The single SNMP2 candidate reported in *I. typographus* [53] was in the SNMP2a group, sharing orthology with DponSNMP2a.

## 3. Discussion

The northern bark beetle, *I. duplicatus*, is an emerging economic pest in forests in Central Europe [1,2,6,10]. Like most insects, olfaction plays a central role in the survival and host selection of these beetles in the forest [11,12,14], and pheromone traps are widely used for monitoring purposes [4,15,72] with limited success [3]. Co-habitants like *I. typographus* further complicate the management strategies. While the olfactory communication in *I. typographus* has been an active research area for over a decade, with crucial pheromone receptors being characterized, the same in *I. duplicatus* is understudied [53]. The conserved responses reported in bark beetle pheromone receptor orthologs [51] make the current search for chemosensory proteins (orthologs) in *I. duplicatus* interesting. Here, we report the chemosensory proteins from *I. duplicatus* sharing conserved orthology in most of the chemosensory gene families studied. The near-complete transcriptomes, with good coverage of full-length ORFs reported here, will serve as a resource for future olfaction- and detoxification-related research works.

Finding 69 ORs, 69 GluRs (a total of 50 antennal and divergent IRs), 25 GRs, 27 OBPs, 9 CSPs, and 6 SNMPs shows comprehensive coverage of the chemosensory proteins in *I. duplicatus* antennae. The number of receptors was similar to that reported from the genomes of other bark beetles, *I. typographus* [31,53], *D. ponderosae* [58], and other coleopterans [48,73]. No sex-specific chemosensory genes were found in our antennal transcriptome analysis. We currently lack the chromosome-level *I. duplicatus* genome assembly to explore this further. Our phylogenetic analysis revealed conserved orthology in most bark beetle chemosensory gene families, except in OBPs. Such conserved orthology in chemosensory genes has recently been reported in other insect orders, like Blattodea [74].

Within the bark-beetle-specific OR expansions in coleopteran OR subfamilies, the subfamily 7 OR expansions are of special interest, as they include *ItypOR46* and *ItypOR49*, the pheromone receptors detecting (*S*)-(–)-Ipsenol and (*R*)-(–)-Ipsdienol [31], respectively. Interestingly, the five-member clade detected monoterpenoids with different ecological origins, and we found 1:1 orthology in this clade between *I. typographus* and *I. duplicatus*. Importantly, *IdupOR23* and *IdupOR29* were found to be the orthologs of ItypORs detecting fungal volatiles, and *I. duplicatus* are known to carry specific plant pathogenic fungi [5]. Reporting the orthologs from a sister species provides valuable insights into the functional evolution of odorant receptors in *Ips* spp. Similarly, coleopteran ORs have been extensively characterized in recent years, especially in the following species: *M. caryae* [57], *I. typographus* [31,51,52,75], *Holotrichia parallela* [76], *Rhynchophorus ferrugineus* [49,77,78], *Rhynchophorus palmarum* [79], *D. ponderosae* [51], and *Hylobius abietis L.* [51]. This allows for more functional comparisons exploring the functional evolution of coleopteran ORs [51]. Characterizing the pheromone receptor orthologs will be particularly interesting, as ORs, specifically, the pheromone receptors, hold the potential for developing novel pest control strategies [67,80]. OR-based biosensors have massive potential in food sensing [81] and pest monitoring [80,82].

We identified 69 ionotropic glutamate receptor family proteins (iGluRs) in *I. duplicatus* and categorized them into non-NMDA, NMDA, and IR families according to the sequence characteristics and phylogeny [23,32]. The IR phylogeny rooted with non-NMDA iGluRs provides evolutionary divergence of these receptors [32]. The number of IRs was similar to that reported from the genome of *D. ponderosae* (57 IRs) [58] and higher than the IRs found in *I. typographus* transcriptomes. While the antennal IRs generally share only a fraction of the total IRs in Diptera, [32] the Coleoptern IRs share a nearly equal number of antennal and divergent IRs. Unlike in Diptera, no species-specific expansions are found in coleopteran divergent IRs. The bark-beetle-specific expansion in divergent IRs indicates the orthologous nature of these proteins and shared commonalities in their environmental stimuli.

We identified 25 GRs in *I. duplicatus*, classified into sugar-, bitter-, and CO_2_-sensing receptors [60,61,62]. The number of GRs reported was similar to that reported from the *Agrilus planipennis* genome but lower than from *D. ponderosae* [58]. However, GRs from the antennal transcriptome are essential for chemosensory detection. The phylogeny rooted with CO_2_-sensing receptors showed two distinct clades that include sugar- and bitter-sensing receptors with orthology between *I. duplicatus* and *D. ponderosae* GRs. This indicates their shared gustatory preferences as wood-boring insects and bark-beetle-specific GR divergence [58]. The 1:1 orthology observed within the CO_2_ receptors signifies their responses to the common environmental stimuli that apply to dipteran GRs from *D. melanogaster*. The large clade with no *D. melanogaster* GR orthologs shows the probable expansion of bitter-tasting receptors in Coleoptera, which include bark-beetle-specific GR expansions. Such expansions are important for insects, as bitter-sensing receptors are known to detect versatile chemical stimuli, including metals, fatty acids, and bacterial components [83].

OBPs are known to increase the sensitivity of odorant receptors to odorants [68]. These are some of the well-studied chemosensory proteins in insects. We identified 27 OBPs in *I. duplicatus*, and the total number was slightly lower than the OBPs reported from other coleopteran species [48,58,84]. The OBPs were classified based on their six conserved cysteine residues [67], and we found Classic, Minus-C, and atypical OBPs but not Plus-C OBPs [85]. Interestingly, we found a tetramer-OBP in *I. duplicatus*, while only dimer OBPs have been reported and described so far in the literature [85,86]. The structural predictions provide insight into their domain architecture, awaiting functional studies. As bark beetle PBPs have not been characterized, our data provide attractive candidates, as the OBPs expressed in the antenna are more likely to include pheromone-binding proteins [69]. Due to the diverse tasks performed, the function of OBPs still remains unclear [86]. Odor detection ability, short sequence length, thermal stability, and easier purification make both OBPs and CSPs suitable candidates for developing biosensors [87]. The number of CSPs identified was similar to that reported in other coleopterans [58,73]. However, the numbers vary across the reported 10 insect orders with low sequence homology [71]. The Dipteran sequences in the phylogeny explain no sequence homology to any of the coleopterans CSPs. However, conserved orthology was observed within Coleoptera, especially in bark beetle CSPs. As different CSPs are known to bind different classes of compounds, functional studies are limited in most insect orders, including Coleoptera [88].

SNMPs belong to a large family of CD36 proteins that perform various functions and thus often require a clear phylogeny to differentiate the SNMP sub-groups [38,89,90,91]. Although we identified six SNMPs in *I. duplicatus* based on a blastx homology search, only three were grouped as SNMP1 and SNMP2 classes. The structural and functional analysis of SNMP1 proteins has proposed their role in pheromone detection as tunneling proteins that transfer odorants from OBPs to ORs [22,38,47,92]. However, the functional distinction between the 1a and 1b groups remains unclear [43]. The SNMP2b protein IdupSNMP2b could be involved in the pheromone-clearing process, according to the proposed functions of SNMP2 proteins in insects. However, unlike SNMP1 proteins, no orthology was observed in SNMP2 proteins between *I. typographus* and *I. duplicatus*.

## 4. Materials and Methods

### 4.1. Insect Collection and Antennal Tissue Dissection

Norway spruce logs with *I. duplicatus* adults were collected from Kostelec nad Cernými lesy (50°00′07.2″ N 14°50′56.3″ E), located in the Central Bohemia region in the Czech Republic. The beetles were reared on Norway spruce logs in a laboratory under 70% humidity, 24 °C, 16:8 h day/night period. The logs were debarked, and adult beetles were collected and stored at 4 °C in collection bottles for sex separation. Cold-anesthetized adult beetles were separated by sex under a light microscope and stored at 4 °C in collection bottles. For total RNA extraction, the antennae were dissected from ~500 cold-anesthetized *I. duplicatus* adults, generating four pools (2 × 500 males and 2 × 500 females separately) under a light microscope. The dissected antennal pools were stored in RNA*later* (Themo-Fisher Scientific, Washington, DC, USA) until extraction.

### 4.2. RNA Extraction and Sequencing

The total RNA was extracted from the four pools of ~500 adult beetle antennae using PureLink RNA Mini Kit (Invitrogen, Carlsbad, CA, USA) as described earlier [49]. In brief, each dissected pool of antennae was freeze-dried in liquid nitrogen and ground using a pestle and mortar maintained at low temperatures. The freeze-dried, powdered samples were then homogenized using a lysis buffer, and the total RNA was extracted using a PureLink RNA Mini Kit (Invitrogen, Carlsbad, CA, USA). The total RNA was quantified using a NanoDrop spectrophotometer (Thermo, Wilmington, DE, USA) and sequenced at Novogene (HK) Co., Ltd., Cambridge, UK.

After the RNA sample quality check, mRNA was purified from the total RNA, followed by library preparation with poly A enrichment, and a quality check was performed. After the quality check, the quantified libraries were pooled and sequenced using Illumina NovaSeq6000 with Paired-End 150 sequencing. The library preparation, sequencing, and quality controls were performed at Novogene (HK) Co., Ltd., Cambridge, UK.

### 4.3. Transcriptome Assembly and Gene Annotation

Quality checks for the RAW sequencing reads were performed using MultiQC v1.15 [93]. The de novo transcriptome assembly was performed individually for each sample of paired-end reads using Trinity-v2.15.0 with default settings for strand-specific reads [94]. The representation of reads to the genome preliminary assembly was verified by mapping all the reads back to a preliminary *I. duplicatus* genome (unpublished) using HISAT 2.2.1 [54]. A combined assembly of all four sets of transcriptomes was performed using Trinity-v2.15.0 with default settings [94], and finally, redundant sequences were removed by clustering approach using CD-HIT v4.8.1 [95]. The candidate coding regions were identified upon predicting open reading frames with Transdecoder v5.5.0.2. To verify the completeness of the assembly, the combined assembly was subjected to BUSCO v5.3.2 analysis [55] using insecta_odb10 with *E*-value 1 × 10^−3^. The raw data for the transcriptome assembly are deposited in the NCBI SRA repository with BioSample accession numbers SAMN42904001 and SAMN42917254.

For manual annotation, we created databases based on the longest assembled isoform of each transcript. tBLASTn searches [96] were performed on these local databases using reference datasets of each multigene family: ORs, IRs, GRs, OBPs, CSPs, and SNMPs as queries with an e-value cut-off of 0.001. The retrieved sequences were further verified by BLASTx searches [96] performed on local databases. The OR reference dataset included protein sequences (Refseq NCBI or published) from *I. typographus* [31], *Megacyllene caryae* [57], *D. ponderosae* [58], *A. planipennis* [58], *Anoplophora glabripennis* [58], *Tribolium castaneum* [97], *Leptinotarsa decemlineata* [98], *Drosophila melanogaster* (Refseq-NCBI), and *Rhynchophorus palmarum* [48]. Finally, all the sequences with insufficient similarities compared to the reference dataset were manually filtered out based on an all-against-all BLAST analysis and subsequent clustering in CLANS v2.0 [99]. Combined and individual transcriptome assemblies were performed to identify the orthologs of *I. typographus* chemosensory genes. All the IdupORs were named based on their similarities with ItypORs. Finally, before the phylogenetic analysis, the candidate protein sequences were assessed for transmembrane domains using DeepTMHMM v1.0.24 [100] and TOPCONS v2.0 [101].

For the IRs, the reference dataset included sequences reported from *D. ponderosae* [58], *A. planipennis* [61], *Inquilinitermes inquilinus* [74], *R. palmarum* [48], and insect iGluR amino acid sequences [32]. The GR candidates reference dataset contained amino acid sequences from *D. ponderosae* [58], *A. planipennis* [58], *A. glabripennis* [58], *I. inquilinus* [74], *D. melanogaster* (NCBI RefSeq), and *T. castaneum* (NCBI RefSeq). For OBPs and CSPs, the reference dataset included amino acid sequences from *D. ponderosae* [58], *A. planipennis* [58], *A. glabripennis* [58], *D. melanogaster*, and the dataset used in [102] and [103]. The SNMP dataset was created using sequences from *D. melanogaster* (nr), *Aethina tumida* (nr), *Manduca sexta* (nr), *T. castaneum* (nr), *D. ponderosae* [58], *R. palmarum* [48], and *Sitophilus oryzae* (nr). Finally, the predicted amino acid sequences of each multigene protein family were retrieved manually from the transcriptome assemblies based on the blastx search results.

### 4.4. Phylogenetic Analysis of Candidate Chemosensory Proteins

Maximum likelihood phylogenies [104] were reconstructed for each family of *I. duplicatus* chemosensory protein, using similar protein sequences from closely related species. GenBank accession numbers are provided in Supplementary Information S6 for the other species sequences that were used in the phylogenetic analysis and are available in GenBank. For OR phylogeny, we used amino acid sequences from *I. typographus* [31], *M. caryae* [57], *D. ponderosae* [58], and *R. palmarum* [48], *R. ferrugineus* [73], and a GR from *I. duplicatus* was used as an outgroup.

For IR phylogeny, protein sequences were retrieved from *D. ponderosae* [58], *A. planipennis* [58], *T. castaneum* [97], *D. melanogaster* [32], *R. palmarum* [48], and *Daphnia pulex* [105]. For GR phylogeny, *D. ponderosae* [58], *A. planipennis* [58], and *D. melanogaster* GR (Supplementary Information S6) amino acid sequences were used.

For OBP phylogeny, amino acid sequences from *I. typographus* [53], *D. ponderosae* [58], *A. planipennis* [58], *T. castaneum* [97], *D. melanogaster* (NCBI), *Colaphellus bowringi* (NCBI) and *Tomicus yunnanensis* [84], *R. ferrugineus* [73], and *R. palmarum* [48] were used in the analysis. Reported pheromone-binding proteins from *Popilio japonica* [106] and *Anomala corpulenta*, *Anomala cuprea*, and *Anomala octiescostata* PBPs from NCBI were included for analysis, and *Lepismachilis y-signata* OBPs [107] were used as an outgroup.

For CSPs phylogeny, sequences from *D. melanogaster* (Refseq-NCBI), *T. castaneum* (NCBI-nr), *D. ponderosae* [61], *R. palmarum* [48], *A. glabripennis* [58], *Bombyx mori* [108], *Camponotus japonicus* (NCBI), *Clunio marinus* (NCBI), and *Apis mellifera* (NCBI) were included, and *D. pulex* [105] was used as an outgroup.

For SNMPs, protein sequences from *D. melanogaster* (Refseq-NCBI), *T. castaneum* (NCBI-nr), *D. ponderosae* [58], *R. palmarum* [48], *A. glabripennis* [58], *A. planipennis* [58], *A. tumida* (NCBI-nr), *M. sexta* (NCBI-nr), *R. palmarum* [48], *R. ferrugineus* [47], *P. japonica* (NCBI-nr), and Scarabaeidae-specific SNMPs from [109] were used. A non-SNMP protein, croquemort (Crq) from *D. melanogaster*, was used as an outgroup.

Multiple sequence alignment was performed for each chemosensory protein family using MAFFT v.7 [110] under the E-INS-i iterative refinement method and trimmed by trimAl v1.4 [111]. The best-fit amino acid substitution model was determined using ProtTest v.3.4.2 [112] and was used for the maximum likelihood phylogenetic reconstruction using IQ-TREE v1.6.12 with 1000 bootstrap replications [113]. The local node support values were calculated using the Shimodaira–Hasegawa (SH) test [114].

Additionally, the signal peptides in the odorant-binding proteins were predicted using SignalP v6.0 [115]. Multiple sequence alignments for OBP and CSP classifications were performed using MAFFT v.7 [110] under the E-INS-i iterative refinement method. For the identified tetramer-OBP, the domain architecture was predicted using the NCBI conserved domain database [116], and the structural predictions were made using AlphaFold3 v0.0.8 [117] and visualized using USCF ChimeraX v1.6.1 [118].

## 5. Conclusions

The current study provides comprehensive coverage of candidate chemosensory proteins in *I. duplicatus*, an emerging economic forest pest in Central Europe. We used multiple antennal transcriptomes to achieve a high-quality assembly of *I. duplicatus* and annotated the genes using a traditional assembly and mapping approach. The identified gene repertoire includes multigene family proteins: ORs, IRs, GRs, OBPs, CSPs, and SNMPs, with numbers comparable to those reported from the genomes of other bark beetles. The phylogenetic analysis revealed the divergence in each chemosensory protein and the conserved orthology in bark beetle chemosensory genes. Finding the orthologs of *I. typographus*, one of the actively studied coleopteran species, provides valuable functional insights and serves as a resource for future olfaction research on bark beetles. Understanding the chemosensory system in *I. duplicatus* also aids in formulating olfaction-based eco-friendly forest pest management strategies.

## Figures and Tables

**Table 1:** Assembly and mapping statistics of the four *I. duplicatus* antennal transcriptomes generated in this study. The transcriptomes are named male antennal transcriptomes (IDUP_AM1 and IDUP_AM2) and female antennal transcriptomes (IDUP_AF1 and IDUP_AF2).

	IDUP_AM1	IDUP_AM2	IDUP_AF1	IDUP_AF2	All Combined
Total raw reads	40,749,080	47,677,670	39,480,112	40,689,528	
Total transcripts	105,416	121,285	91,822	98,264	204,588
Total genes	61,659	70,337	50,264	54,391	125,878
GC content	39.89	39.82	38.96	39.26	39.21
N50 length	2101	2027	2455	2036	2317
Average length	1049.33	1011.11	1244.16	1048.26	1027.03
Complete BUSCOs (insecta_odb10)	93.71%	94.37%	95.17%	94%	99.71%
BUSCOs fragmented %	4.24	3.58	3.22	2.37	0.15
% mapped to genome *	88.56%	88.30%	87.97%	88.63%	

* Preliminary genome (unpublished).

## Data Availability

The datasets generated for this study can be found in the NCBI repository as BioSample 1: SAMN42904001 (IDUP_AM1: SRR30040105 and IDUP_AM2: SRR30040104) and BioSample 2: SAMN42917254 (IDUP_AF1: SRR30041954 and IDUP-AF2: SRR30041953).

## Supplementary Materials

The following supporting information can be downloaded at: https://www.mdpi.com/article/10.3390/ijms25179513/s1.
