# Supplementary material for: Antennal Transcriptome Screening and Identification of Chemosensory Proteins in the Double-Spine European Spruce Bark Beetle, Ips duplicatus (Coleoptera: Scolytinae)
Source: Int J Mol Sci. 2024 Sep 1;25(17):9513. doi: 10.3390/ijms25179513 (PMC11395090; doi:10.3390/ijms25179513)
Supplement: Supplementary file 1 [file ijms-25-09513-s001.zip › Supplementary_File1_Johny_etal2024_v2.pdf]

Supplementary information for

Antennal transcriptome screening and identification of chemosensory proteins in the double-spine European spruce bark beetle, *Ips duplicatus* (Coleoptera: Scolytinae)

Jibin Johny[#], Ewald Große-Wilde, Blanka Kalinová, Amit Roy

Faculty of Forestry and Wood Sciences, Czech University of Life Sciences Prague, Kamýcká 129, Prague, 16500, Czech Republic

Table of Contents

Supplementary information 1: Protein sequences of complete chemosensory-related proteins identified from Ips duplicatus in this study. ***2***

Ips duplicatus **odorant receptors** **2**

Ips duplicatus **Ionotropic glutamate receptors (iGluRs and IRs)** **12**

Ips duplicatus **Gustatory Receptors** **25**

Ips duplicatus **OBPs** **28**

Ips duplicatus **CSPs** **30**

Ips duplicatus **SNMPs** **31**

Supplementary information 2. Predicted transmembrane regions from IdupORs. ***32***

Supplementary information 3. Predicted signal peptides in IdupOBPs ***54***

Supplementary information S4. Conserved cysteine residues identified in IdupOBPs. ***62***

Supplementary Information S5. Conserved cysteine residues in IdupCSPs ***63***

Cited Supplementary Information References from the Manuscript ***64***

Supplementary information 1: Protein sequences of complete chemosensory-related proteins identified from Ips duplicatus in this study.

Ips duplicatus odorant receptors

>IdupOR1
MDDKQTIISRSVSFKALILARVLPDRGRDSLAYYLSVRVIFTFVWLLVASDAFYIYSRI
NDNYGFEVVEFGLVCIVSLSGVMWITMFCTKNRHLLIALAQGLSDFTAFGFPPNFEK
FMQQLNFYSKIHLFYLTGGALMYFLLFAPLHKRNCDELKQEKNLSEICSLLLPLNAPF
VEYQSFGKFPTLQLLNIIIFLSLMYMYMCCGTIVWLNVELVEHIRIRIRHLKLMILRAL
KSNDREFRRKKFREAIRYHEYICSMSGLADEFFGTELFLHVVLTGAILGISAYLISNGS
LETVMIFVGWLNAIIMGSVAGQRLINESLGISDIIYKVDWYNFETALKKDILFFLLRSK
KPMFIRAGNVVINNALIIQILRTAYSYVTFLSSINKX
>IdupOR10
RASHSPRSSQFSEEMFNPTNYQLIKWTRLSMILAGVWNFPLYENNILQKLYEIYAFA
MKCGYAVFFSCLVGETLRLILCNYELSVVFATIGILFNATKISIKVLIYIHFDIFQLFEEII
EKEKEVWHSGNQDVITMYKKKIRICTMYVLVLAASTFMAIFFLELSGAVATYKLIEH
NQLNNDTLEPHFMYQTLFPLNKLDHIPLFFASQAFWAWSGNTFNIMTHGVFVTLLIY
AVSQLEILQIRMRNFVGNVQTEILEPEEVNQKIVILKVLIQDHIHIIEVVKYINNATKYP
MLLEYLLTSLDIASVSVNLIKSRSEWLWLLVFLLLLVIQISLISWTCNEIRVQSVAVGD
ALYESQWYLLNKEAKFLVGFVIARSQIPLSITIGPFGTMTTGSALTVFKAAYSYMTLM
KEX
>IdupOR11
SGLLALHGLEIVSEKMIQVLKLFDLDSQFTKNYQIFFWLGFWKPDKNYKHKTLYNLY
TVFCFGVLITFISPQLIYMIVFGSDIYKLTSTLYVFTSFFMNLVKTLGIYFNMDVIKDIL
RNFKDNPLLQAKFEKHVKIAKETKKRGDFLFLYGTFLGFGTQLFWTIYPFSQNKRML
PVNGWYPYNPMNSPNFELTSFFQNFASAFNIFHTMNIDSFTINLMMQTGLQCDFLQL
TLKHIMQFHTVDGILCKNLQQNTESLEDTLTPNLKTCIRHYIEIKRIARKLEDIYRTSM
AVVFVGGAFIFCTIFYQMLHSQSNPTEIFYLLFFLFSMLTEQFIFCWFGNEITFKSGQIH
GALYTIPWVDCSVKFRKMLAIFMISLARPISLNAGRIIPLSAKMFLQVMRTSYSYLTLL
NSLX
>IdupOR12a
MADYQRYDIQIFLKDERLSLGITGFVPTHLKCHLPTLVTYVLVLGQVMASAYYGLSS
TDLAEISASFMITITHINTIYKVISVQLRPLSTLDQIIEKPIFTLVDHHEMTILRGTLAKC
QHFLTLYSVTIVGSIVFYVVAPLVANYKTHEKNYPTLAKFPFNPDDYYWAIFVGELF
ASSLSALCNGCVDRLFAKHVAIATGLLKILGHRIKHIMDVNDQQVIEAKMKYCVLY
YNEVIGYAKQIENQFSFGILFQFLCSCLVICLTEFQFLVATSSGTFGLLFAYLACMITQI
SIYCWYGHQLMEESNSISMEFYNLNWLDMSIKNQKTMLTSKERAKNPITLKASRVFS
LNLTTLMTVSREGATSSATVDWFQILRASYSCFAVLHQVYTKX
>IdupOR12b
MADYQRYDIQIFLKDERLSLGITGFVPTHLKCHLPTLVTYVLVLGQVMASAYYGLSS
TDLAEISASFMITITHINTIYKVISVQLRPLSTLDQIIEKPIFTLVDHHEMTILRGTLAKC
QHFLTLYSVTIVGSIVFYVVAPLVANYKTHEKNYPTLAKFPFNPDDYYWAIFVGELF
ASSLSALCNGCVDRLFAKHVAIATGLLKILGHRIKHIMDVNDQQVIEAKMKHCVLY
YNEVIGYAKQIENQFSFGILIQFLCSCLVICLTEFQFLVASSCGTVAVGLLFTYLACMIT
QISIYCWYGHQLMEESNSISMEFYNLNWLDMSVKNQKTMLTSKERAKNPIVLKASG
VFELNLATLMKILRTSYSYFAVLHQVYTKX
>IdupOR13

MANHQLSDIQKFLKEERLFLGITGFVASNVKYHLPKSVIYILTSMQVLASVNYGLSTT
DLGEITAAFMITISHINTLNKLFGLHLKSLSALDRILDKQIFALADNHELTILEKTLTAC
QNMLTMYLVTILGSIILYGATPLVANYKTFEKNYPTIAKFPFNPDNYYWTIFAGEFFIS
SLSALSNGCMDRLFAKHVAIATGLLKILRHRIKQITEVSDQKVIEAKMKHCVLYYNE
VIGYAKQIENQFSFGILIQFLCSCLVICLTEFQFLVASSCGTVAVGLLFTYLACMITQISI
YCWYGHQLMEESNSISMEFYNLNWLDMSVKNQKTMLTSKERAKNPIVLKASGVFE
LNLATLMKILRTSYSYFAVLHQVYTKX
>IdupOR16
MYNIPENERENYFLKFSRMTMLMLGIWPVRRGGDLLEKLYESYFLTTFLYYIAFNLS
GLALAIITWSNNYLTTTSSMGIVIEYMSNAYKVLLFKSSIFKGLIKEIQDRERDIFEGSD
EAFKEIYIRNAESNKKVVLFYTIMGTSGISLYFITPLVSNVLMPLGYNNVTGVHDHYFI
VFNWFPFDPNRYYWAAYLIQFTGCLIGYSYIVHCGAFYISILNFIRTQLKILRHVIVNM
SEYSFLYKTTYKLTEEQSQFVLLRAIVLEHQKIISFVKRTNYTIQLFTLINFVISSFQLAL
LVYQIFQVAILQQITVLSYFVTLSTQLLLTYYAAHMIVFEVX
>IdupOR17
MGLMTTPRFFLQLWGIWPVDTLTAKLSQKLYIMYGFCIIGWYSFFNIFQFIASIRLVLN
NESFDRISRCISVMVTVVLMLVISLIYKQNGIPQLCSTVLEIEADLAKSKDEKVTQAYR
TALAKSKYLNIYIVGSSLFTLSVFTGLSLLDVIKVGPTFWDFDNVTFMHELYLPFNRR
NHQLLIITTNIFTACESVVVNGAIQTTFYALVMYGALRFKILRLNLKKIDQTEGDRKW
RMRELIQDHQYCIRFVEELNQATKNVLLMSFVLNSLKVASILFPLMAISEFTDLAFPLI
YSSMLVSEVVFQGWICNEVTVQSVQVAETLYDTFWYKESKQYNVLLQLMLMRAQR
PLTMRIGPFGAMTTSTILTTMRAAYSYATLMMNSSX
>IdupOR19a
MMYEVRKDLPFYFSLLILGTMRFYPLGQKSKKRFFVELIVMLILGNVICADVIVLFGS
SLKNGDYFLVTSSFPFALAIIVINVSILSFAVNKKKWSNLFKSLADCHKFGKPTNYDL
LKKNGDRKGMIYMTCYISTCAMCAIIEAVEEQRCLKNVSKHEICGFIIPVWWPTNYR
PSTFIKTMVQLYEVVSIIIFSNFTIIATLQYQICEYIAAKASHLGLNFNAIDPTSDSQIQFE
QFKFFVHYHQYIISLCAEFDCLCKRTVGHVTFTTAAISALFSYHGMQGNYKLLAFLA
LYILNLGFMCHTGQNLEDAMLGISNSIYSSKWYELDIRVRQWIPLLLARTQKRIGLDA
VPVGYLNYALFMTVLKTTCTYLNLLNHTIX
>IdupOR19b
YISKRTVLMMYEVRKDLPFYFSLLILGTMRFYPLGQKSKKRFFVELIVMLILGNVICA
DVIVLFGSSLKNGDYFLVTSSFPFALAIIVINVSILSFAVNKKKWSNLFKSLADCHKFG
KPTNYDLLKKNGDRKGMIYMTCYISTCAMCAIIEAVEEQRCLKNVSKHEICGFIIPVW
WPTNYRPSTFIKTMVQLYEVVSIIIFSNFTIIATLQYQICEYIAAKASHLGLNFNAIDPTS
DSQIQFEQFKFFVHYHQYIISLCAEFDCLCKRTVGHVTFTTAAISALFSYHGMQGNYK
LLAFLALYILNLGFMCHTGQNLEDAMLGISNSIYSSKWYELDIRVRQWIPLLLARTQK
RIGLDAVPVGYLNYALFMTVX
>IdupOR20a
MEKPTYYSVGKTLPFYTTLAILQYFGYYQPQSKKFPKGLVAWTIIWVLGLLNLLVLII
TRLHDGDYFAASAAIPFAIGLTVANLINLLFALKQDTWSKLFLSVTDFSICGTPTEHLV
LKQKSDKVAKFCVYFLTAFMTFLVVMALLLESNCLKGNDKEYICGLLLPVWWPSGF
EVTLLTKRFFLGFQLVISLATIPYIFIAGLQYQVLQYIVAKVRHISQLIKTVPHQNSVEQ
SRDFNVIITYHNHILDVSYRFNLVCRGTFGHVTLTIAVVIAFWSYHFIAGDVKCIAYIFI
YLGNLWFLCHTGQQLEEQAAIIGNSFYSSDWQRYSRSIGKTIPFVLLRSQKQVTFDAV
PIGALNYEFFFAVIKDX
>IdupOR20b
MEKPTYYSVGKTLPFYTTLAILQYFGYYQPQSKKFPKGLVAWTIIWVLGLLNLLVLII
TRLHDGDYFAASAAIPFAIGLTVANLINLLFALKQDTWSKLFLSVTDFSICGTPTEHLV
LKQKSDKVAKFCVYFLTAFMTFLVVMALLLESNCLKGNDKEYICGLLLPVWWPSGF

EVTLLTKRFFLGFQLVISLATIPYIFIAGLQYQVLQYIVAKVRHISQLIKTVPHQNSVEQ
SRDFNVIITYHNHILDVSYRFNLVCRGTFGHVTLTIAVVIAFWSYHFIAGDVKCIAYIFI
YLGNLWFLCHTGQQLEEQAAIIGNSFYSSDWQRYSRSIGKTIPFVLLRSQKQVTFDAV
PIGALNYEFFFAVIKSVFSFISLLNQTLX
>IdupOR21
MILPNNQLLKIPMYLSSTVGIWPFIFEENKYLKRLYDIFATILYYYYLEFICRSYYQLT
VLLRAKKLNVEEILGNLCITLIYTCSMFRLKAFNTKEIRTLFSNIISTENQILQGADLQV
RQIYLKGVKVNRIYHILFFVNGWVVTLLYFLHPFFMELPTMVVNNETITLKTLPLST
WWPIDIQKHFWPAYWWNVFDGTLGSSFVINSDMLAFSLVAFVVNQLDIISYKLLQL
KAPTENDWKDNMECLIKLIKHHQTIIKYIDTFNTGLKYVILFDFLQCSIQLATITLQLL
VMKINVQNVIFVSEFFITMLIRLVIYYYNGNEVIFKSEKLAMDIWNTDWYLRSNEDK
QLILIFLIRAQKPLKFDIGPFGALSLPAFLSVIRATYSYMMLFINTNQX
>IdupOR22a
MYCCSIFGCFPAKLMFKNHESLQKIYAHYGNVMLTYYTWMTFSIYLELVQTLQTDL
NRMDVISGILSIGLINTATVLRQLTMKFHPNFTELVQKVVDLEHTILSIRIPEVVEAYN
SFVDRYNKNCKFYLYFMIILTLNFFIRPLVVKPYEIQAGNQTVVIKTVPLPIWMPFDK
QNHYDLAYTWATVNAIIGASYVAISDVFMCTLIAHPFGLLTVLRQILIHFEKQTQICQ
TDELMSDEDAEFYTFTCIIKWHNQIIEFVDEYNRVMSGVMLFDFLQSSVQAAFIVVQI
LESGITLMILSYVLFFMLTMLFRLILYYHYGDEVTCLSQSLAYEIWDTRWLEKPPRVK
YMMRMFIMRGQKSLTYDIGPFGTISMAALLSLLRTTYSYVMLFEGFSRQSNVGKLTL
DSEHX
>IdupOR22b
MYCCSIFGCFPAKLMFKNHESLQKIYAHYGNVMLTYYTWMTFSIYLELVQTLQTDL
NRMDVISGILSIGLINTATVLRQLTMKFHPNFTELVQKVVDLEHTILSIRIPEVVEAYN
SFVDRYNKNCKFYLYFMIILTLNFFIRPLVVKPYEIQAGNQTVVIKTVPLPIWMPFDK
QNHYDLAYTWATVNAIIGASYVAISDVFMCTLIAHPFGLLTVLRQILIHFEKQTQICQ
TDELMSDEDAEFYTFTCIIKWHNQIIEFVDEYNRVMSGVMLFDFLQSSVQAAFIVVQI
LESGITLMILSYVLFFMLTMLFRLILYYHYGDEVTCLSQSLAYEIWDTRWLEKPPRVK
YMMRMFIMRGQKSLTYDIGPFGTISMAALLSVCTYSILSKRKTQLCFSFVCSFX
>IdupOR23
IFAVCKCSTSATMAVYPKSENLKVPAIYCSTVGIFPWKFMFQDNKSLQTIYRCYSIVM
LSWCINFVFTEYIQLAILLTSETLDMQEISFNTCITLLFTCIGLRAVIIYFSPISEDIIQSIID
SEKVTYLDDAECMKLEKKHLGSVRLISHCYFISLLFSTVQRNVYFFSKEPDFIQNGNE
TEIVKVHMVSIWFPFDREKHYLTVYIIEVIDSFIGTFFVACVDMYSFNMISYPKGQLK
KLQYMFKHFHNYKAKDRFETNGENDFIVFKDLVQRHKKIIQHINAFNESMEFVAIFE
FGQSSAQIGCVLTQTSLEDLTIGSFLFVMSFLISMLVRLFLYYYTANEVTMESTNLAQ
CIWKSNWYEESQKLKLSMLMVIIRAQKPLIFRIGGFGAMSVQSIVTILKATYSYITLVY
RRYPX
>IdupOR25
MKVYPDTKFFDVTAKYGSIVGLYPWQFMFPDNKTYRQIYRWYSHIVFLTFSVLLLTL
YIELIILLKSKETSKDELGENLSITIVFTSVGLRAVLLRRGSNLITLIQNVMDEEKKQISA
DSKEVQLLEDKCLKVVRKLSYIYIITAVLTASQKSIRVPFQTSRKDGNVPTRDLILSA
WFPFDKHEYYWKAYCIQIYHTIIGTSYVLCLDVLMFNLLSYPIGQLKKLQFLIVNMK
VQHYTDNYIENKSVDNVVRSIIERHQYIIQYVDFYNTYMGTFALFDFLQSSVQIATVL
LQFSPTPGTIIFIIIFFVLMLLRLFLYYYTANEVTVQSEKVGKAVWESQWYEQPQKIKH
ALLLIMTRAAKPSKYIIGGFGVMSTYSIIQVLKATYTYITIMFRX
>IdupOR27
MRVYPDIENFKITAIYSSTIGLFPWKLMFQDNQMLQQMYRVYSYFIYGSFVVFIITAYI
ELIIMLNGDVLKMDAICSNICLTLAFTCSALRATVMRVGPNLLKIITQVMRAEKNRAS
IEDQTSLKLERKFIKTMRKLSHLYAVAITMIASSKCALAPFEKGETVHIGNTTIIDRPLII

SAWVPFDKNTHYWAAYIIQIYFAALGAWHVAYVDMFMFNMLGYPIGQLKKLHHYI
KNILTQNDDSLEEFKNVVRQHQQIISYVQFYNDSMGTFAIFEFLQSSVQIASIFIQTSPS
DMNLGQFGFIVGFFIGMLFRLFLYYYTANEVMTESEKVGVSVWESDWYEQPTHLK
MALLTVMVRGQRPLYYKIGGFGLMSVQSIVAVFFHLIX
>IdupOR28
MCQLDSANMGLYPASRYFKNSIMWSSVLGAFPWQLIFEENPKLQQVYRWYSNFML
TWYFGLVISEYIQLYHILNANVIQMDEVCENVCLSLVFTCTGLRVWVMRSTSGLSEII
QTVVDAERETDGLDDKKTRQYEDIHVANMKKVTFIYSLFVFISVTYGCISTLYEDTK
TVIIDNSTIIIERPLIISSWFPFDKNEHYWVAYGLQVFDGYMGALTVACTDSLMFSLIS
YPTGQLTKLQHLVRNMAVFKTDIEAFTTFRKLVQRHKHIIRYVELFNETMGTFAIFEF
VQSSVQIASVLMQNSPDDMPLMSLCYIVLFFVSMLTRLFMYYYSANEVILESIKLGDS
VWESRWYHQPHKLKQGMMIVMVRCQKPVSYKIGGFGIMSMQSIVAILKATYTYISV
ILRNX
>IdupOR29
MSGYPKCKNLRVAIIYSSIIGVFPWQFMFQQNRLRQTLYRWYSVFLFSWFAGFMITE
YIELYVQCTADELKLDEICANICVVMVFTSTAVRQLVMRFNQTVNDLIQSIINGEKQS
NFLDDDETKEIEDKFIKASDSISNWYAAPVYIALLQYVFFPMLSKPDIIQVGNTTQALR
PLIVDSWFPFDKMEYYWIVYTLQMFDMIVGALYVTYLHILMFNMYRYPVAQLKKL
QHIFRNFERYKVEHMKLSNYNERHSAFVVFRECIKKHNKIIQYVDGINDCMSTYTVF
DFLQSSFQIAALLVQTSPNDMTFSSFLIVFTFITTVMIRLFVYYHSGNEVIFESVNISMA
LWESNWHQQSPQIKSMLLLVMRRAQKPLCYKIGGFGVMSLQSIIAILKATYSYVSIIF
RQX
>IdupOR2a
MRILQRETDITFFKFNIWVLKTCLLWPEDLNYKYDKKRFFKDVTMVTSLMPCFLPIL
ADFLQQLYEEVPDLTEAVENMIALNCLIGMFYMVICFVRNRRMIIQLMIDIRTFNKYG
NDSITHEVDNKANLFSKVFMFYGILGNFVYMAMPQIRVSKCHLNRTEDMIEKGVPC
GLVVRSYFPFKFDYSPVFEIVFVHQIYTCTMVSVVVLVLTMLFCGFLMHIVNQLKHL
RVLIARLKNVPPKSFEKKLIFVVRYHVAIIQYSQNTARAFSTMLLFYITLTSVVLSVLC
FEILMVDAFAESVRFTLHLLGWLIILLSICYNAQLVLDQSQEVANDVYSLDWFNLPV
DVQKKVQTVIMRSQKALVMEAGGMGIVSLPAFLKVLSSAYSFFTLLLKFKX
>IdupOR2b
MRILQRETDITFFKFNIWVLKTCLLWPEDLNYKYDKKRFFKDVTMVTSLMPCFLPIL
ADFLQQLYEEVPDLTEAVENMIALNCLIGMFYMVICFVRNRRMIIQLMIDIRTFNKYG
NDSITHEVDNKANLFSKVFMFYGILGNFVYMAMPQIRVSKCHLNRTEDMIEKGVPC
GLVVRSYFPFKFDYSPVFEIVFVHQIYTCTMVSVVVLVLTMLFCGFLMHIVNQLKHL
RVLIARLKKVPPKSFEKKLIFVVRYHVAIIQYSQNTARAFSTMLLFYITLTSVVLSVLC
FEILMVDAFAESVRFTLHLLGWLIILLSICYNAQLSQEVANDVYSLDWFNLPVDVQK
KVQTVIMRSQKALVMEAGGMGIVSLPAFLKVLSSAYSFFTLLLKFKX
>IdupOR3
MAENDLQIMRLHTTILKCLLVWPISRLSERQNLIAKSAAFMFSFTCSLPVFAATGYQF
QSGIDRVNILLEALIGVHNIIGNAILYIHFLRSQQKIQEVIDDIKLLVKYCGLKTIKDAD
AEIYNFSKYLLGYVTIGVICNLAWPLLSVKNCLISRGTEFYIQHDPCGMPTRNLYPFD
ASHGLSFWILFAIEAVFCYHTCCVFSLATVTLIGFLKHILVQLRYCGHEFETIFDGVDK
KIAGKHLTLQHFIKVVKYHQEILRYTEKVFSTFNVMIVVYTGVTSFILAITGYQITSPE
TGGEDRIRYTMLIIGWTLLFYWICYYGQQIQDEASKIADAIYNSKWYENTDTVVLVR
RDIIIIYLRTKIVLDFKVKFLGAVNMEVFVAVMRRAYQIFTLLLSVTX
>IdupOR31
FRMDIYPKSDHLNFSFALCFMLGILPWRLVFQDHYFLQALYYLYSKALLIVNLIFITTE
WMEVCRILNQDPVNLTDLNNAIAPVLLFTVTAIRMIIFNSNPDFMRLLNYIINRQGFM
AQRDDEIRKPSQKRIKVNKWIGVAYLILYLGVIYQLLALPLVLGPFEQQTANQTTTIR

FLPLLSWIPFDTQQNYWACYLWQALNLQLASSNICHIDVLMLALILYPIEELGYIKHV
FRNFASFKSRTGIENSNFASFTVFKDVIRIHNNVINYVDTLNDTLSFVMLLDFLQSSLH
IAVILGAVVVGGPTDLASLSFVGTHFFSMVLRPFLYYYYANQVMVLGANLTEEVWN
VDWFDESKDVKYMVQFFNMRAQKPLQYFVGSFEVMNLQSFISILRVAYSYVMLLHT
LQX
>IdupOR32a
MSXFPESKQLRLVSILCGLIGILPYKFISPDKKLYQDIYRAWSFLSNLIFVVGMFLAYT
KLYTLFKEEKLRFVELSRNLAVTMLCTMTMARQIIIRVKPEVXNMLSQILESEEAILV
KNDKTVAGIYLKNADNLYRKSVYFWFIMLNVVVSHIVRPFFIFEEIQQGNVTVIHKTL
TLSLWFPVDNQEYFWECYIISSIYAVSFASFQSYTDIFMYAMITYPVGQLQILYHTIKN
FHMYKTELKSNFDGMSDSTAAKLMMKNCVDMHLLIIKYIEDYNNCMSILTVFDFFQ
TSIQITSILSQIVMMEITVFLAVFILIFLTMVFYRLLMLYYYANEIILTSEDLCQAVWQS
EWYNEPPQCKFMMQIMMMRSKTPLMLKIGPFGSMSLRAFLSILQASYSYFMLVYSQ
KDNX
>IdupOR32b
VYIAVAMFAALIGLLQEQPLRMGEIFKNVATTLLCSVSLGRMLALRLNPAFVKNVKF
MIEFEKIIQSTEDDQVKQIYKAYGKQTNLIAYSYIVSVFFLTFFYILRPFLLGNSDSDVL
VDDNYKSFPMSMWFPFDSETYYWCALTWSCINTIVMASSVTSNDLFTYLLLIHPVSQ
LKILHHILRNFDVYLKKMNGLYRVDGVNMDAFTLKACDDIYRNIIQYX
>IdupOR33
MAFFNSVKNKLFGIFLSLGHYICILPEIQSSKSNLENVCRKVYTVSILSLALFLHASQIF
ALYQTTTEEIFIFDEVLRNYLITSLHFSCLLKCVFLRGSDSKLLIQQILDFEETLERSDD
EEIKKMYKYHVDLAASVKKKFITGVLLISMFYSSAPVSREPYHIETGNNTIMVRQIPM
SMWVPESWNYWVASIYSGSAGVFLTVYFTTADLTLFCYIAFATCQLHILKQNIDNFH
KICLEIAYTKGCSKEESYRLFQKECIIFHQEIISYVHVLNRNLKNLMLIDMVPGSIQIAC
SLFQMVKNLNLVQCIILCEFTLTLIFRIFMFTSTIHNMGDLSQKIGSAWYNMDWMELP
RDVRNNLMFCIMRSQRPLWITLGDIDTISMGSFLAVLKGAYSYLMVLLTVX
>IdupOR34
MKFRELIHNDFLGICIQLGYYFCIIPEKAAATDKKESRNYFFYSCIIRALILYCHICQWV
KMYQIITADIFIFDELVRNCAITSIHFQSFVKTSIFRRNYRLFENIINFENALYKNNDQK
VLLIYRDTLRAIKNSRLVYVLGTLIVIVFYIAAPLFRGPYYVKMGNETVTIIQLPLSAW
SPTDNYFSNFAVTGAMGAYLAMVFVQTDLLYYCFLYFSICQLNILEHYIMHFYQYCD
EFMNDHKCSHVRALTLTQKIYIKYHQNIIKNVKQLNDAFKNSLLIDLVPSSIQFANQF
YIIGTNLNIMQCVCIGFFTIMLMSRVMAYCYLANQISVHSQRIGSAWFQINWSDLPNE
MKKMISFCIMRAQKPLVITLGNFGNVTLMTFVGILQASYSYVMLFITLX
>IdupOR35a
FGRKCNFRLRGQTLKIEHDSVCSILVESFKSDFIMTGYQPKFLLFTKRLATVLGIFPSK
LIFQDNRLKNQCYAIYTKFSLGLFNLYLLTSFVQLFVIITSNPIDFTELSKNLIITPLFTV
TVIRQICMSQPVFIKLIQHLISHEMYLDTTTDKEVIKIRQTSDQMITRNIVTYLVMMIIT
EISYTIRPLLEQETTVVRENYTVLVRPMPLSTWFPFNPDQNFYVAYIIQSSNIVFCSTFD
TVGEILLTTVLVYPTVRLKILRHVLHNFQKYEDNFTDPHSADRLMKICLKIHMDIIRY
VEEFNTAMGTCMFLDFIQSSIHMACVLAEILTGDVSLIELVSTSAYLVILNFRLFIYYY
YANEVIVLSQDMGVAILYTNWYEKKSAVSYGTLRIIMRSQKALKYKLGIFGDMSLSK
YLSILNAAYSYVMLLTTVKX
>IdupOR35b
MTGYQPKFLLFTKRLATVLGIFPSKLIFQDNRLKNQCYAIYTKFSLGLFNLYLLTSFV
QLFVIITSNPIDFTELSKNLIITPLFTVTVIRQICMSQPVFIKLIQHLISHEMYLDTTTDKE
VIKIRQTSDQMITRNIVTYLVMMIITEISYTIRPLLEQETTVVKENYTVLVRPMPLSTW
FPFNPDQNFYVAYIIQSSNIVFCSTFDTVGEILLTTVLVYPTVRLKILRHVLHNFQKYE
ANFTDPHSADRLMKICLKIHMDIIRYVEEFNTAMGTCMFLDFIQSSIHMACVLAEILT

GDVSLIELVSTSAYLVILNFRLFIYYYYANEVIVLSQDMGVAILYTNWYEKKSAVSY
GTLRIIMRSQKALKYKLGIFGDMSLSKYLSVSNATLYSHGVVLLFCRYX
>IdupOR36
MASDEFIKIPKIFLILGGFWPFAVTKNPFKSKLYKVYSKFQTYSYVTLIASLVLKLVILI
LRKEDETLIFRNVNVLVLVFETCVKILIFQTLKIPHMFEHAMKHERNILKSDDQELITC
YRNQVKYGRRVNLSQFFVTTFSTSTFAVTALWDVYWAADMSKYEKEPFMHDLWFP
FRRETHMFWVILFNLFMDAQGTCFNTATQATLINLIIYSSSRLKILGLKIRKFDPTATE
NVLETVRELIFEHKDLLSFVETLNVRIKYVLLMEFILNELGLASGIIQLIVTDTPSYMVS
VVTIIILQLFQIFVMAWTANEITIQGSKIADSVMESNWVDQPTHIKKLFLVMVMRAQR
PIGLTAGPFFNMNANTAISTVKAAYTYLTFMTRNYNX
>IdupOR37
MFSATQWLVMASGIWQLDIKEKYRIPYKIYKFYIRVLYVVVAILIHSNLFYYLGKDN
NRALEIVAHCINLFQCLIKLAIFMRKRVVELVKIAHEDTFNSTDNIPECNQTLKKCKS
YVAKISLFNAAYCVSVIAFFTTYGGFMDYKRLRKCFAEGVEEPEYVFNLWYPFSYRK
YLKLVFFLEAFLLIQACTTSFSVISLLNTLTMYVVLKLKLLQFGFKNFDRKFNTNNGN
WIISHSEAIENLKSLIEEHQGIIRYCTELDRHLKTVLLIEYSITSLMLASLFLQLMQGNR
VELFTPYFIMLVFQLFLLSYNAEKIKTESGKISWAIYESNWFLHGPEAGYLTGLIILRS
RKVLSLNIGPFGPIGLDAAMDRVKLAYSYLAVLRX
>IdupOR40
MPIAANEKLILWTKTLMIIAGFWNQPISKNPIIRKLFFLYSIMMRICCILFWVFLFLEML
RLIVFGYEWEIIIPTLSVVFTDSKVMAKMIIYMKNNILDIIPEMTKKEKEVWESESKEI
KTVYLNKIKFLRIATIAVATTNFLTISLLELMGVVATIQARDYNKVYNGTIEAHYMY
QVLLPLDKIKHIYWFFSTQVIWAWIGYIFSVVTQIVYFTVLCYAAVHLEILQIRFKNFV
ETSFNFSASVEALEKKTEVLKGLIQEHQQIIKFIRTFNQNSRYITMMEFVMSSFDMAM
VVVPLLKVDGRPQDLSQMIFWIFYFTVLMTQLFLIGWTCNEIKIQSGAIGTALFESNW
YLLNHEARKLIQMVILRTQTDLTMSIGFFGPMTTDSVILVLKGAYSYINLMSPRNX
>IdupOR41
MEAKKNLNKKLISWIRIIMIITGYWDIPISSKKFVNRLLHVYSVGMRASCFLFWIFLAA
ELIRLIIFDYPEEVIMSSVAIVVTQLRVITRSWTYLKNNTLDIFNQILNGDQEIWIYSKR
EIEEVYRRKTKFLNIGAAAFVMADAMTVISLDILGIIGTLRIHSHNLANNDTLESHLM
LQLIFPLNNMAHLNFYLLSEMFWSWIGFTQNVATVLIYVTSLVYAVTKLEILQVKFR
CFVEDDYELCSSNDKIQEKTDMLKNLIREHQSIILFVTQFNEKVKYITMAEFIFSSLDL
ASVCMNISKDVTIEDGWVFFYCFLMVTQLFLIGWTCNEIKIQSENIGIALFHSKWYVL
NQEAKQMILCVITRARRPLLMTIGLFGPMTTDSCVLILKAAYSYLNIMKKX
>IdupOR43
MGRTLFKICRTLAFYAGLQKKDSHLHPHYTYHIYKVVTTIVHLVYIFCLPSLCIALCQ
NVGKDMDAALALMKNITFVAVVVFKTVIVQSDAIVKLVKVASVEEEKIRNLTDQTIR
KMYKSNVDCCNRVAKIIITNLYASGTMYALDGLYRSYTFYKTHSNVEPENPKPHTV
MLWFPFDHNLYYKTAMAYEIFHIFQTLNYNAVAQSVVSSVMVFLKIELKVLQHHIR
AIQGGSRDYQIKVLIKCAEKHQQIIQWVNDFNSNFRSIILLEYSMVSLTLATSLIDILEG
TKIPFNVTFFTLNFIQLFVLAWNANQISYESSKSLSDALYACSWYEFDKTTQEFVLFM
TLRCKKPLTISNGPFGYITMNAALSRVKLAYTVVSVLSTTKX
>IdupOR44a
FSQKMYTISKEKPFYCTLVLLRAFFWYPDTPKCSLSFILCSLVLRLLSILVALGTLVHL
VLNFNEETKAAISEDIGDLTGFVGCMSSCLNFLWHHSNWSSFFNRLLDFKQFGTPPG
YAKMVRRGNLTTLACILYTIPGMLWYSYLTHLDIPRCEALNRKFGMKEACGMVNPT
WIPTGYDRGNGWRFWVLYVLQCTGIFVYLPSNFVIANIPLEAVGIIVTRIDHLNSKLK
RCGSDLGRLYHCVKYHQDIIEVCKELSDLVQATMGTLLLTGAIVIGSLGSQVIKASTP
KAVTFILGYTTTIFVVCHAGQKLINQSLTLADQVYWMEWYQQDPKIRKDLRFVLAR
CQKPLSLVGPPSMGFAGYSLFLIMLKTSYSYLTLLNEVILX

>IdupOR44b
QKMYTISKEKPFYCTLVLLRALFWYPDTPKCSLSFILCSLVLRLLSILVALGTLVHLVL
NFNEETKAAISEDIGDLTGFVGCMSSCLNFLWHHSNWSSFFNRLLDFKQFGTPPGYA
KMVRRGNLTTLACILYTIPGMLWYSYLTHLDIPRCEALNRKFGMKEACGMVNPTWI
PTGYDRGNGWRFWVLYVLQCTGIFVYLPSNFVIANIPLEAVGIIVTRIDHLNSKLKRC
GSDLGRLYHCVKYHQDIIEVCKELSDLVQATMGTLLLTGAIVIGSLGSQVIKASTPKA
VTFILGYTTTIFVVCHAGQKLINQSLTLADQVYWMEWYQQDPKIRKDLRFVLARCQ
KPLSLVGPPSMGFAGYSLFLIMLKTSYSYLTLLNEVILX
>IdupOR45
MTGTINIKNHNQANNDTLESHMTIQVLLPLNRMNHLKWFFGTQWFWAWNAITCNL
SSLLIYVNVLVYSVAQLEMLQIKFRNFVEPPFSLDATTLEVKSKCAMLKELIREHQHII
RFIKDFNNKMKYITMMEFIFSSLDMASVSISVIKvssgsfflNGTVGWQCFYVSLLITQLFL
IGWTCNEVKVQSEAIGDAIFESRWYLLNKDAKMLVAMTITRSRRHLEMTIGPFGPMT
TNSIVLVSITECPTMYIX
>IdupOR46
MKAFPDSDSLKAFKFTSVLGLFPWKLTFQQNKFYQTLYYWYSLFVLCWDIGFVFTS
YVELVILLRGEVLNIEEICTNIRITTIYTCIIIRLVMIRTSSGLLNLIQEIIDSDKQVTNMD
DEETTKLVKKDTSNNNLKFIWYVSICCSIGLQFFIRPLVTEPETIQIGNTTVVAPKDLII
LTWFPFDEQKYYWVAYFLQVIDGIIGTLFVALSDVFIVNLILYPTTQLKKIQYIFRNFE
YYQQSYKTLNVGETEESAGIKVITELVQRHQRIIKFVDTFNGWMGPLMVFDFLQSSIQ
IASILISDLRRDITFAMVCFIVTFFVGMVLRLYLHYYSANELILESQKLADAIWYSNW
YEQSPKIKYLMLIVIIRAQKSLKYNIGAFGIMSLESGIAILKATYTYITVFTSNNX
>IdupOR47a
MYPIRKDLAFYRTLIVLNFVGLYSFNDNRKQTRITYIIVRAIFRNLWSLDALLLVIVSG
INGDYFMLTQAIPVLLGLLVINTTNLLFDCNQKRLSDLLISVSDWTFGKPPGYDRLQK
TGDRVGLVYLVSFVAIFILIGIRQVLYEEQCLEVTGNEDLCGYVVPLWWPPIFERSTL
NKRLALVCVLINVTAISPFTLITTLNLQVFQHIDSKVEHLLLLLKEIPDCATEEEQLKLF
IKFIQYHQHIISLSAKLNNFCIRTLGHVTLSTAVVIGFLSFHAILGNYDFILYIILYFFYL
WFMCYPGQLLEDRMLSIGDGLYFSDWQKYSHEIRKMIPFVLLRCQKPVTLDAVPVG
KLNNALFLAVVKSTFSYFSLLNQTLX
>IdupOR47b
PVLLGLLVINTTNLLFDCNQKRLSDLLISVSDWTFGKPPGYDRLQKTGDRVGLVYLV
SFVAIFILIGIRQMLYEEQCLEVTGNEDLCGYVVPLWWPPIFERSTLNKRLALVCVLIN
VTAISPFTLITTLNLQVFQHIDSKVEHLLLLLKEIPDCATEEEQLKLFIKFIQYHQHIISL
SAKLNNFCIRTLGHVTLSTAVVIGFLSFHAILGNYDFILYIILYFFYLWFMCX
>IdupOR48
MDLYHPLNRKSPFYFTILALKILHFYPTTSSQSNPKLFYSLSLVVRVLTSFVCLECLLH
FLAAVKENGNVDLSEDLSILTGVSNCILVCLLFQANLADWSRVFKQVADTSKFGIPPT
LPQTIEKTNFLSLFYFTFCFVGVQLYAVVNVLSTNCDPIKEVCGTASNFWWPGPSPSIS
TPVKAVIIIAQVLSMTFYSPSSAILTYIPWEATTLLVCKIDHIKLLLNDVVKVKGCQVQ
RARLHHCVRYHQEILRIFHDLRGCFKKTFSVMVLTASIILSCLCAEMLKSFAPGTVVH
FSGYSAAIFLLIFAAQKTWDESEDLQDSVCGLRWYCLDTTLIKECQFIVLRCQRPARL
DTIPFGVLDYSLLVTIVKTAYSFLTLITRTX
>IdupOR49
CNMKMTIYPKTENMRLTAIYSSTLGIFPWKFLFQDRKFFQKLYRYYXIFILTWYIGFV
VTAYIELFVLLRGETIKMDEVCTNMCLTLVFTCAGLRACVMRYGNRLNDTIQGVIDT
ERDTNLLDDENVMEYENRYISVMRTFTHCYAASVIIPXAQRSVFVAIANPQITEVGN
DTVSYKPHIMSSWFPFDKQEHYWPAYWCQVFDGAMGASFVAFVDIFMFNLISYPVG
QLTKLQHLIRNLKTYQDKAWQAGVQRADLTVLNDLVRRHQKIISYVDFYNNYMGT
FAIFEFIQSSVQIASVLAQTSPDNLTLEETSFIVCFFISMTIRMFLYYFSANQVIVESRKV

ALSVWESNWYEQSPEIQKSLLIMMIRAQKPLCYRIGGFGIMSVESIIAIMKGTYTYITII
YRGYX
>IdupOR4a
QLQTMDEQSQILKFHIKILKFLMIWPFDNLNRNQNYYLMRGCFAYACFCSIPVFSGA
AFQFCVGIDNVKVLLEVLVGVGNITGYNIAYVCFLKNQEKIEFLIKDFQEFVQFSGLEI
IRKTEEKTTQYTKYLLSYASIGLVITFIWQMLSTESCETQRGGDYYVRHDPCWLPVR
NWYPFDASQPKLFWIVFPIEAIYSIHICLFFSLATSTIIGFLMQITSQLQYCSNRFEHVFD
EVDVKQFQQIKSDYLFLIKYHRKILDYSKRLFNVFDALIVVYISLTSFIMAIICYQIVDP
KISAQDRIKYAILLIAWCLLVYLICYYGQKVQDEALKIGQSIFKSNWYGGTTAIALKP
YILFTLARTQVPLEFKAQLFGTISLLQFMKVMKWSYSGLTLLLAVTDEDX
>IdupOR4b
QLQTMDEQSQILKFHIKILKFLMIWPFDNLNRNQNYYLMRGCFAYACFCSIPVFSGA
AFQFCVGIDNVKVLLEVLVGVGNITGYNIAYVCFLKNQEKIEFLIKDFQEFVQFSGPEI
IRKTEEKTTQYTKYLLSYASIGLVITFIWQMLSTESCETQRGGDYYVRHDPCWLPVR
NWYPFDASQPKLFWIVFPIEAIYSIHICLFFSLATSTIIGFLMQITSQLQYCSNRFEHVFD
EVDVKQFQQIKSDYLFLIKYHRKILDYSKRLFNVFDALIVVYISLTSFIMAIICYQIVDP
KISAQDRIKYAILLIAWCLLVYLICYYGQKVQDEALKIGQSIFKSNWYGGTTAIALKP
YILFTLARTQVPLEFKAQLFGTISLLQFMKFVIGQKKVSQTNHCAEIYAHVNLHNSLF
LLVX
>IdupOR5
MLNHDYPKNVLSSVDTILMICGLGKISKTVPWVIRLAYSIYNYLVMSIAFIFLIFELIAF
KMALDDLPTFLSQIAMVLTHCAGFVKLWMLVNMMNSMEKIRNKLQDGRFKYVPV
GNFQPGLKMRKAKALMSRVTVLIFTMYSFVGVSAHISAGADVIKNTHNGKFMEGIT
CHNIIPFNFYIPFDISTPTMCHYALMYMNISLDAQAFYIATFDLIFVCFLHLLSAQLDIL
SDAFTTIRERSLKKLEMDPEKQCFYDDQCSELEKEMYREITHCNQHLNLLIEVSNDIE
HVFSLITLIQTSASLLICASCLFVAAKVQGSSPTFFSQLEYTAAILSQISLYCWFGDKITI
ASAQIPMALYKSDWLSCSQRFKISMLMAMTRMRKPLYVSIGKFTPLALNTLLAVLR
GSFSYFTLFQRAGX
>IdupOR50a
MADLQVDSPFYVSRHFLAKVLRLFPNSSKSNQRQFKIFLYIWLGFWSGPTLNNGVLF
MYNLANGNYEMFTEDFSYLLGHIVAMLSCYRLGIDYKKWANLIDIVTEHKWGKPSN
YEKVKRQGEFKAKLLILYLTLMLTLFTIFVILDEKTCELAYQRKRICGLLDPTWWPKT
IQLTGTFKRMLLIYQMLAAYFMSGLFYATYFVLQSNEFIVAHVDHLINMFEEICASTP
EEQFAKFKEWVSYHNYIIGLCKTLNVLCIRSLGLLSVLVPVTLACLGYQMLLNRTLK
GTVYILGFLILITVVCHTGEKMEMAMTKVGTTIYLAEWQKCDWKIQQWLPFVLEKT
QCSIGLDVVPIGRANHVLLLMMLKATYSYLTLLKQTMX
>IdupOR50b
MADLQVDSPFYVSRHFLAKVLRLFPNSSKSNQRQFKIFLYIWLGFWSGPTLNNGVLF
MYNLANGNYEMFTEDFSYLLGHIVAMLSCYRLGIDYKKWANLIDIVTEHKWGKPSN
YEKVKRQGEFKAKLLILYLTLMLTLFTIFVILDEKTCELAYQRKRICGLLDPTWWPKT
IQLTGTFKRMLLIYQMLAAYFMSGLFYATYFVLQSNEFIVAHVDHLINMFEEICTSTP
EEQFAKFKEWVSYHNYIIGLCKTLNVLCIRSLGLLSVLVPVTLACLGYQMLLNRTLK
GTVYILGFLILITVVCHTGEKMEMAMTKVGTTIYLAEWQKCDWKIQQWLPFVLEKT
QCSIGLDVVPIGRANHVLLLMMLKATYSYLTLLKQTMX
>IdupOR51
MLWTGICRSTSFATSVGRNITATISFTIQIIYVIALMNYVITNFNNLLKILQPLLLTFTTV
NVLAKTAAFAIKADVFTELDELMSDEILYDTARESKIFIKKQIASTVWIAKCYRIWTM
QALLAYLVTPFVMPLLGYRMESIIFPWNTNKLIHYFLANLFQGFCGIIVTTLNTTTDTI
FAILCSICTTELRVLKRNFQTLDFLSDDSFRRAELKKNLQLYNKIQEITGIIEEMYTYGL
LAQFLGGAVIICLTAFVLTSANTDENLEDMLVKNSFNILYFVYLLLQVSIYCLYGHDV

MTESNDINEAIYLSNWYRSDRVFLNNFLIFRERLKRPIILTAGKLFPLTASTLVELLRV
SYSFFAVLRNMQHFX
>IdupOR52
CLAMVLVLTDLFYYCLLYFNICQLEILEHYVKNFYEYAEKLMENCNYCTKKEAFRM
MQVKCINFHQQIIRNVQILNQTSKACMLVDFVPSSIQFANQFYIMFTNLNVMQCICIG
CFTTMLMARVLVYCFLASQLSAQSQRVGTAWFQLDWKEIPMELKRNMVFCIMRSQ
KPLVLTLGDFGNITLMTFLMILKGTYSYVMLLTTLHX
>IdupOR53
MINSNEKLVRILKFNLVIIGFWKRPLTSLKLNLIYRYYSKCTILLFRFLCLSIWAEFIRLI
VQKYDYETITSGLLVFMYTIKVTLKLFAYKQLKVVELLNEVIETEDKIWKYGDKDV
QAFYQQKVKLSNSFAATLTGITFSTVLAVVTMCMHHTTTKLKFCQRLNESLKFVIFV
EYAVSSLDLATAVTNFLQADSTRGFFQQTLAVCLLTTQIVILGWAANELKLQSGRVA
DAIFESPWFNLNPDGMRMIKFVVQRAQKPLVVTIGPFGAMTTQSAV
>IdupOR54a
MEIFPNCKQLMISMYCCSALGCFPSELMFRNHKTYQKAYSLYGKIIFAYYMWLMCTI
YMELLSHSLNIDVVSGILSAGLINSATVFRQWVIRFDTRFTKLVQKAIDMENAIDSIQL
PEVVDAYNSFIKKYNRNCKAYLYFMVVLTLNFFIRPMLVAPVETYVGNETITTKALP
FPCWLPFDKQKHYEMAYVITWLHTILGASNVAISDVFMCTLIAHPLGILTVLRQVLV
NFEKLAQINRHENNSLNIKDAEYYTFIDIIEWHIKIIEFIDEYNILMNGVMLFDFLQSSV
QVAFIVVQVLGSEITPIILAFVISYILTMLFRLVLYYYYGNEVTFLSERLAYDVWDNQ
WFDKHPKVIFMMRVFIMRCQRSLVYRIGPFGTLSMAALLSLLRTTYSYVMLFNRFSD
EX
>IdupOR54b
AIIDVFMCTLIAHPLGILTVLRHILVNFEKLSQIYQQENKSLNIEDAEYYTFIGIIEWHIK
IIELIDEYNNVMNGVMLFDFLQSSVQVAFIVVQVLKSRITPISLAFVISYILTMLFRLVL
YYYYGNEVTFLSERLAYDVWDNHWFDKHPKVIFMMRVFIMRCQRSLVYRIGSFGTL
SMTALLSLLRTTYSYIMLFNGFSDEX
>IdupOR56
MSMQNFVAYPKKILKFSAIWPEKKPSFSYNLRTLFTLSTIFLLMICLTYNAAFHLNSFV
KLSESLYMLISIVNTLLKVVMLSSNGQVFMDLMAMMEMPSFTKYSVQYSEVVTQFK
RLLKIVENVYWVQVNSTILFLSLFPIVEEESLPFDFPHFNEGKYHYPFYAFEVLSLYIS
AYDNMAVDLITVSAISLIVVQLRILNRKLIDTELNVRNSPDFMEKNIEEDVLRYVEEC
CQHYCDIEKYTNQIFKVFSVIVFVQLGTSIIAICNAGMLVITVDPFSIEALSLMLYMVT
MFAQLTMYCWFGNFVYVESLEIMTSCYLSHWDKRGTAVRRALFMLMERVKRPLKI
QGLKFVTLNFDTLIAILKWSYSYFTLLKNYSKDKLNX
>IdupOR57
MLRIVLLPQRLSEKKAQHPAKPIKNPEHINNTQCTCSFAVISKIFSSYKLDNVSSLHKS
LTIRTFQIIFKSLNLICENNEIQFQVKDNVDKVYRNCRTDAX
>IdupOR6
CILLTKSHKVMSEKHQRYATDFFSVNQWMLRRAGLWRPSTKNQYYQFCYTLYAISV
FIFVNLWFTSTEFISLFYTYKDKYALIKNVNFFLTHFMGAIKVVFWYFRGQYLMVIM
QDLEDPNYHYEGYKDYQPGIISQKFKKQGTKYSLMFLILAHATLTSSYVFSTITTIQH
MKGNSTVALPDRLPYYSWMPFSYDTGPKYLLAMAYQAGPMFSYAYSIVGMDSLFM
NIMNCIAGNVTIIQGAFKTIRERALPGQSKNVLRESKADMKVLKAELRKIVNHLQTIF
KACDKLEDVHRMVTLCQVTATLFILCTCLYLVSIAPPLSKQFLVEFVYMLAMSFQLY
LYCWFGNEVTIKFQELPRYIWASSWLATDTQFKKALLFTILRTKRPVFLTAGKFSRLV
LPTFMSILKTSYSIFALIRNTSKX
>IdupOR60a
KMLLEKDFFSFICRILQFLLLLPPSSSSVALNVLYWVVAVPHFSLLSLVAVLEGANFFF
GEEEKFSTNILNLGTVILHAMTLNRVIRWLFIRNEYFKVLNKLRKISEDFRAYSPLLKE

YHEKVMVATGKQCQRIVVSLLVFVPVNLFFSYFFNYLSPVKVRWNPGLNKTSTYRD
YPYPVWFPFDTSLSDGYYWLGYFYQLYAFQFLVSGFFCVDWLCTCTIIHLTSLIKVLR
RTFQLIDEPVQPHEIIFTIKEQRLKYSINKLREIYECATLLNNTFSSQLLIQEVCMAGVV
CCCVYRVTEKTSTVEASSLSTIVSAAFMELFTVSWFNHNFTLGFFKILRDIERLDWLN
YNIKLRKMLVFVMMRIQKPFYFTMGLGIPLDMKVFMTMVKSSYSLYTLITQSAESD
NVPKKCEX
>IdupOR60b
KMLLEKDFFSFICRILQFLLLLPPSSSSVALNVLYWVVAVPHFSLLSLVAVLEGANFFF
GEEEKFSTNILNLGTVILHAMTLNRLQLKHEPNTLKLHISYRVIRWLFIRNEYFKVLN
KLRKISEDFRAYSPLLKEYHEKVMVATGKQCQRIVVSLLVFVPVNLFFSYFFNYLSPV
KVRWNPGLNKTSTYRDYPYPVWFPFDTSLSDGYYWLGYFYQLYAFQFLVSGFFCVD
WLCTCTIIHLTSLIKVLRRTFQLIDEPVQPHEIIFTIKEQRLKYSINKLREIYECATLLNN
TFSSQLLIQEVCMAGVVCCCVYRVTEKTSTVEASSLSTIVSAAFMELFTVSWFNHNFT
LGFFKILRDIERLDWLNYNIKLRKMLVFVMMRIQKPFYFTMGLGIPLDMKVFMTMV
KSSYSLYTLITQSAESDNVPKKCEX
>IdupOR61
MAQVHIDILQYLKPEQIRLGIGGFYPSRVKRTLLIHLFTTCTYLFIFGHITLVVNYALST
NDLAKVSEIFLLSMTQISFLNKLINFHLNNWKLAELDKTLEQDVFTKGTATEQKIFSK
TIASGQRALWFFYGICVIGVLAYWLAPLIDTATTGRKSYLFPAKFPFNPDDYYVIVFIA
EVIVLIMSAWINANMDCLFVKHAVIATALCKILRKRITTVSRNTEDDRIVEDRLKRCI
VYHNQIIAYVSKIQDIYSYAVLIQFLCSAIVICLTGFQILVVSPKNGKIGLAIYMLCMTV
QLVLYCWYGHILTDESNEITSACYEIDWHNLKLTNQRMLVIIMERAKRPLALQAMD
VFSLDLFTLMTILRSSYSYFAVLQQLYKDX
>IdupOR63
MHTVLKEWTSIHNTTIHSPKLKKTLFNITLKAKVTFVIGFVLTTATVVIDSYYFIQDSL
LLVILDRENYLTRDSLLYKVLIKVLYLWNVMLISMSVSAPFYYIYGLLQDKVAVLLV
TDYVYGISDKYKHSKSLAYSVKEELDF
>IdupOR64
LVASCGISYEWRYHGFCQLLVQEKYWGFLWPLLMLTYRFLNFIAFITVLNVPQQCLY
NLNNLKFHLFVLQHQIENFEEGLKSKRVNPRFVMKVIISKHQEILRFNAFLREKANKII
IIMAFSVLIALIFIAQQMFSRQFSLLVSFILVFNSVYFIWLITYHGQLLENMMENICERL
YHLNWYEWDARSAKTLHIMLMNISIPLNWNAVTPSHSINYGYFKEYINLLYSTGRLF
YSVFYQNNX
>IdupOR65
RQYLQPPLAFLFILIMTVFNTLSIFSLYYFFYYLLHFKYQFRLFDVYIKRIAAEIGKCGS
KENNDEEQLKVKRALFAVVERHNILKEWGLFMSRSGQAILLMYILVGGFLVICFGIA
GLFIKDDIRVLFWVIILMSIGYVHLLAYFGQEYENNX
>IdupOR66
MPTQILTEKAPNGLLPTMYNSIQLTKQLVPFIRINMVVNIVLIIVHTTYFSWKFDSSSF
MQFSSSFIVNVHLVLIQAIWVRIEDKVVVVVTKYIKESLQLKRQITDLQLKQLEKNVIK
KAYFTFLWITGCVLFLVIVNRNYSFDESFVEHLVEHHHHLSVGILLDIIGILLIIIWTTIS
TIMMVSTPICFLYVMLQGKIAVILLIDYVCSINHMKCCCETTMKHLKVIIKKHLEIIRY
NQFVLKSNQYFYLLYLFNEIFFGLLPMFEYFSPNRSIVAVSSASIYFFCLVYAVNDAGE
EYVTLIDYFSTEIYNLKWYEWDVSCRKVYSILITYLNEPMKVDFVVVNINRALFWKI
LKTLYPVLNFFYTTNRIGRVX
>IdupOR68
MNSILESNQVLYFLYIYNGVLVALLSMCAYFSPENRVLQAGVNSTCFLFFIYMFDDSS
EEYVQLCHELAYVVHNLDWYNWNLQSQKTYLTVVTYMSRPVNVHILLVDINRELF
TKMLKFLYPMLNFLYTTRNQYKX
>IdupOR73

MFQVTKTDPFLPFIFKFLKFHRNIKHLCTIFTIILTVLIVPQTYQLCRYPSPEVVLKYGT
FYLSCIYVVCINTLYLSEAEQVNKILSSFKPLFIVNTENILDQTTTKIKVIVARTNALLVI
WFSAAVFCVEALMYYNCIFDKGERMLIVVFLKQHTNCSSFVIELIKYAAFISVQFACI
CSTVPIYFIYINFYLKLNLIILTDQVKRFNNNLRRSGPSYQKAVIKFITREFLKINRYTL
DIMEVQQVKCILFVCAGLLVGFMGCCTFFSVNLIHFSVIFHX
>IdupOR76
QKLLKLTILKANALLVCLSVSAFICAFMNSYYWGLRVDERLLILSVLHEHLVPADSLF
SKLLLYSLYASTLHTTIVSVSAPLYYIYGVSLQKVMLLTLMEFVVQFKDQCKRGPVQ
EKYVLDNLKLLALKLMENKRYYSIMFETSQLIYFLYICDGMVFAMVGILSCFPPYGH
AVLGIVNCSVFLFFLFFINDTAEGYTEYGEILNATVSDLPWYNWTVKSQKTYLLVLV
QLSKPFKIVLLMWLVVSRDLFKKLCQILYPVFNFLYSTKRX
>IdupOR77
SRVSENMLPKESPNNFLPEMYQFLRLSKYLQPLFTFYIVEYFILFLIQSAYLGKLYKEG
LLIQYLSFYCANIYMLFVTLIHRSVIQIIDINLKSKAEIFYVNSNIFHPKVAKCADKLVK
KIKRNFRLLMILSFAVMYLVTKFWFCSRDIVIFWVLNQQFNYSDSFALKFLNWLIGIS
NLMTVLPIASSPVYFIYVLFHVELEQLCLCGYVYKFKDECKLAIENRRVTIETLRCND
EFEYFVLNQLKCITKKGIKVKSYIKYFFTSNQTLYFMYILNGNITLAIYASLIYFSQAPT
YIGILGVIVFIHCIYVTCECCEDFQQFENMYSEAINNLTWYNWNKKCQQTYKIILIDSC
RNREFTIFSFISVNKDLFKKNVKTDLYSSKLFLQHHVVYX
>IdupOR9
MVHSIVNLTKFHMIVIGFWKIPLTENVWHQKLYKYYSHFASISILLYTIMLTIRLIQLVI
EGQTPNAKLYRCFTINIVIYMMTANLIIFRRYGLPDLISEVITDEEASLNSPDKDIRNAY
LAQTKVYQFTSVAQVVSTFASGLMFIALNVYMKLKGLLQHEAFMYELWFPFSRENH
EGFVIFFNLYIVVLIMFCNVASRIIPQTMIIYANAQLMILQIRLKKAFDAPCPDPLVKIQ
ELVKKHQDLINFITFLNSALRNVIFMEYIINAINVAAGLLQFITVRAAMDLVYAFVHFS
LLVIQIFVLALNANNVSTQSEAIANAAYDSQWMDQNNNVKKLIYIMIMRAQKPLVL
NIGAFGVMNAESALTTMKAAYTYVSIGLQRX
>IdupORco
MMNKFKVAGLVADLMPNIRLIQASGHFMFNYYADDSGSLHILRLAYSCMHLFLVLL
QYGCIFGNLVREKDNVNYLAANTITILFFTHCLTKFIYFAVQSKLFYRTLGIWNQSNS
HPIFLESNNRYHALALKKMRNLLYIIIVGTIISACAWTAITFVEDSVHEIPDPDNENGTI
VEAIPRLLIKSWYPFNAMSGMMYYIALVYQIYYVFFSMFHSNLLDSLFCSWLIFACEQ
LQHLKEILKPLMELSASLDTYVPKSADLFKSPGSATSQDNLVDNDFNAKDDLKGVYS
TRQELGNLHFRSGALQTFGQGGGGVGPNGLTKKQELLVRSAIKYWVERHKHVVRL
VTAIGDAYGVALLLHMLTATIMLTLLAYQATKINGINPYAATTLGYLIYSLAQVFHF
CIFGNRLIEESSSVMEAAYSCHWYDGSEEAKTFVQIVCQQCQKALSISGAKFFTISLDL
FASVLGATVTYFMVLVQLKX

Ips duplicatus Ionotropic glutamate receptors (iGluRs and IRs)

>IdupIR01
ENLTCATVKGSAVDMYFRRQVELSNMYRTMEANNYDTAERAIADVKAGNLMAFI
WDSSRLEFEAAQDCELVTAGELFGRSGYGIGLQKGSPWSDDVTLAILDFHEGFSX
>IdupIR02
MNNLTVMFKWITVIFVVLLCNSGIHSEYQIGGIFSDRVHHVAFQVALNKLSQGFDLV
GESFNVSYFNSLDARKAVCTLLEKRVIGIIGPSSTHTSNYIQSICDYKEIPQIEFHYDTKI
SRNRCVINLHPHPSEI
>IdupIR03
MSHKLFYCSIILVIPCFALGTRIKQEIMASMTENSEDSFGILLQEIIVKSVRSYKCLAILS
DQLYLPLFQESWFQVFHDYVSYVLVSVDESEDLLAPSNETQWSLLLAKNNGCQMYII

LISNGFQMGRFLKFGDRYRVLNTRSKYVMLFDNRLFEKPLHYLWKRIINVVFIKKYIS
KKTANVRKTEWFELTTVPFPINFGDVLIPRRLDIWAQLKFRKGVDLFKDKTADLRNE
TLRIATFAHVPGTVKSNQSVSNKIRANIRLSNTTEVFSGMEIEILDTLSKVMNFQCELY
EPVNADTELWGRKQYGTGVFTGLLGELFTSKADMALGDLYYTPFILDVMDLSIPYN
TECLTFLTPESLTDISWKTLVLPFSPIMWACVLICLLISSLSFHLLSKFHVSTSKYKEES
KRAKVNATDESNKRRMQNISLYSQMTKLDFNMKYSILKDQYRRPKGTGDPEGLYQF
SEPGNSVLYTYSMLLLVSLPKLPTGWSLRMLTGWYWLYCLLVAVAYRASMTAILSK
PTPRVTIDSLQELIESRLTYGGWGEINTEFFKSSSDEFISKMIWNFEFVTCSDEAVSRVI
EGNFAFYENVYFLKEAIAKQLQQPGKRENLTDNHQTNNNRNLHIMKDCIINMPVSIG
LQKNSPIKPRVDKLIRKLLEAGLINKWLNDVMQKILIEGTQEKTEGQKALMNLNKM
YGALVVLAIGYFLGVIALVGELYYYQHIEATKPGFNRYSKMVYAFKKSX
>IdupIR04
APNSERAFANNQTTDYELLQKAEVCEIGFHNNCFDQVTLKKNTAFLVEKGAADYVK
VEYLKQEALSFHLNLLMVKGFWGYDMINDLISYSVDSGLATIWMKSALDFNKTERP
FTNRK
>IdupIR05
MGVVLDGDCPNAANLLERCQRYKVFDSKHFWLVFHTNKNYRYIFEKANLNVDSNI
KVVYPKMGNNSTYLIDDVYNPAYGKGGTLKSFTIGRYTNLNGYQTNERLNKYFLRK
NLTGVQFNSAIVLPEPFEGLLKDYLMNDKMVEVNTLNRFHARLMQSCMSYYNYSV
KIKPIKSWGIFKPDKTMDGLVGLLGLKLVDFGSSPLIVKKTRREFMSYGKNTWPLKM
AFLFRNPTTKKRYLIFLKPLSEQVWLIIVFCSVFLVLAQYFGYKFNPRDLTNKKDEFW
SLSIVSTFGAFCQQGVITFPDSLSGRMAALCNLLLGLMVYQFYLSTLVSFLLNVPGSVI
NSVKEILDSGFNIGFENVLYATALLRESSSETVAQVLKQVSVSNNSGFLTREVGLNLV
KQGHYAFHVELVTGYPFIRKHYSESMVCELKSVSLFPPMFMHANYQKWSPFKDLLD
VCLHRLSENGVINRELIFWHPKKLECIRSASTINIHTGLESFYPALVVLVLGILASLNIL
LLEIMWFKIQKRQVLPYTEX
>IdupIR06
VAQSQWLLPFTTPADPVMIKRKFPSGSVVVNTAPSSISTTVFHNFGCQIFVLDVLDPV
HTFEILEHEIRRHKESLHSRKYLILTTGNLLKIPLRIVGDGLLVNVSKFLTWPGKEEIEV
SIWKLQHLTQPQVLIDKWFSINQSFLWNYTISPEDHSDLKGYIIKVGTFDHPPYVVVG
KNFRVGSTGIVDISGADIEILKTTAQHLNVRLEFHLTSGLWGEIFHDKNASGLKGLIM
KGVLDLGAGX
>IdupIR07
MLQILFFAYLCHYVPSIKAEYNLVAFFEENENGLNKKAFDLAVSLVNQKTEEFQLTPI
YDDLIAQDPFNAIQKTCSVMKNGVLGIFGPKSTCNINAMQSVCDEKELPHILTRWMY
YPLRPGTAVNFYPSASLLAKAYWEIIDKWQWETFTVLYEDDESLLRLSELIITAKNQG
IIVTVEQLDREGSGSYRDALKRVWKTKQKYLVIDCHIGNLVDVLVQCQQLGLMTSE
YSYFLTNMDAHTKDLSPFQWSETNITGIRLINPENPYVQEISSQLFEDADFGDIGVILA
SQLETEAALLFDAVHMFSETLNKLHITEPPQSLDCEQSNSWELGYSLVNLLKTSSYK
GLTGPIEFNNEGHRSSFGLQVYEVREGGIINVATYNSTLGRLNVTRQHEEEEIYDENS
MRNKTFVVIISLTEPYGMLKETSDNLVGNERYEGFSIDLIYELSLLEGFNYTFTVQHD
GKNGNKDPVTGKWTGMIGAVINGIADMAITDLTITSDRAEAVDFTSPFMNLGISILFQ
KPTKSPPNFFSFADPFAIDTWIALAIAFVVVSLSFFLLGRICPDEWNNPYPCVEEPEYLI
NQFTMSNSVWFATGAMLQQGSEIAPIAIPTRLVSGVWWFFVLIMVSSYTANLASFLV
SESNIELITDVNSLVENAEKYGIRYGSKMSGATMDFFNKSTSNELYQKIAKHMQEHP
EDMPTENKDGVAMAETMRYAFFMESTSIDYNTQRHCNLKRVGDQLDEKGYGIALK
KDSPYRNKLSTAILKLQSSGVIEKIRKKWWEERKGGGQCTGPAEDAEATPLDLQNVE
GVFYVTIFGTIFGAILVLFEYAFHILKVSKKKRIPLGQTVKQELKFFFKFGSNVKPVLE
GSEEDDKSEIDSHPKSTSDRSKSKSEKSRTTANGDSIRPYGFVISPSLDRLTDTPX
>IdupIR08

APDIWMYALSAYVLVSITMFVVARFSPYEWHSPHPCDFENEQLNNQFSLANSFWFTI
GTLMQQGSDLNPKATSTRIVGGIWWFFTLIIISSYTANLAAFLTVERMITPIENAEDLA
GQTEIPYGTLESGSTMTFFRDSMIETYKKMWRFMENRKPSVFVPTYEEGIKKVLEGN
YAFLMESTMLDYVVQRDCNLTQIGGLLDSKGYGIATPMGSPWRDKISLAILELQEKG
EIQMLYDKWWKNTGETCTRNDKGKESKANSLGVDNIGGVFVVLLCGLAFAVVIAI
MEFCYNSKRNAIAEKRSPVTPQQSICSEMGGELCFALQCRGSRQRPALRRQCSKCLS
GGVTYAASSLDIPPHPPQPPARSCPQHIHFVSSSKX
>IdupIR09
EETRIKFSSEMFLKIFLFICLLSYSLQFQKQSMSILSCLRFYQPRSVAIFTCWTRQDKVK
LQRTIQNGNLNVFLVFANTDSLAFINRKNGDRFTYFLDMDCPGADNVLKQASNLDR
FGKFQFIYSWFLLLDDNRNVNVLKQIKTRMDMDVKLVEFGSNQIWEVYNPGINIGIQ
TRLIATLNEDYIQLHSNQSESYYESRRNMSGVLIRSANVIRYPFTTSFHEYMVDPKLM
KYDIYSKFHYQLFLCLSEIHGFSLQVLRPAQLVSGIEIPIFRSYK
>IdupIR10
MLFRLLYFTFLFHECTNLLPTLKPAFKPTNFLEKVISRSNATVLIVNSDIQLGLPCMYF
STDRQIVADQVCDNEEVYIILNSIDEEVFEVVKKMAMYCLAEAHILVLEKPNQTSLN
YLETNGIYKIVFVDTHQLNEFNGWPSSNRTFNVCYTVHVPYTTIDNHHSYGLESLIIN
HSGNFARIAVKLIKHTEGCTSGGSMGVFEHLKNRSCDVVIGAVMCNEILPFIMSSPYF
LEDGTVLVTPNVKCSRQLFSAPIVAPVLWVFISLISVEISQIKFKI
>IdupIR11
KIWYPSSTRTKFNLTVVLRITNPYVSANGTGLEEQLLKYIYDWLEINVTFMYLPFGAT
AIPYKNEKMALWNHSIDIFGGHILVQPEDVEEFDISPAYLFDSVRFMSPKPRNVSITER
LFLIYSPLYWILMSTSAILSAVLTKVFFNIGFLDKLLDYIAIFVGFPVKHFDTNIVSLRV
LLVVWLFSLMVLSKMLSNILFVLHATSLKTKVINTFDDVVQSGLPVYSSLDLKKHYV
LVEEIEKLNSMDVRLCHNYVTCLYNVIDNQNCITVGGAGIAKFYHLPKLYRQRGEL
MFHVSDESIFSFPLMFLYPKGHPVYKLASETFLLSLSNGWLINKYKNLKSIAKLHKRI
NLQYRYQLSLKEFDVLCLWVIGIALSLVVFATELLFKCCINQLVKKNLFLSLTIKKVE
NCEYGSIRLERRIRVSYIKAILACLARLKX
>IdupIR12
MKIVYQNLIVLCSFAAYNYFCLAKDNVKIAVFTNERQSKQELNEWTQIGEKIDFDVS
EPIVVPDTDSFAITKIVCKALAENGYTAVLGPRHRISSEVIGSMCSNLQIPYFQTHWTPI
NEKQYPGTFNLYPDANYFSQGLVTIVKSLDWDKFIVMYENEEGLAKLQDVLKIQLF
KDRSDKNSILLKHLGSGPDYRCVRLIRFNLNLNAHYFTGLFX
>IdupIR13
MDLITEIANLVNITFEFQLTKENSYLNLVNDLVERRADLGICDFTITPQRSELIDFSLPF
MNLGIGIIHKQSTQEEVDNLYAFMRPISWTVWFYIWTLSLASSVTMFIVARLSPSEWE
NPKPWDPESKEVENIWNVKNFLWFSVGAITAQGCDILPKSVAPRIISASWWFFSLIIM
SSYIANLAAFLTMQKRDVTIDSVEELAAQSRVKYGLMSKGSTEMFFSTSNNSLYQK
MWNTMKNEKPSVFEKDNPSGVERVLSTKNELYAYFMESTGIEYEMGRKCDLRKIGG
QLDSKSYGIGMPLNADYRHTINSAILRLQESGKLMELKQKWWEKEREGEPCNRVTE
EQSDALALSNVGGIFIVLAVGVALAYIIAIIEFLWNVRKLSIDEHLSFMETLKCELKFA
CKIYIKKKRTKPVVSESSSSTKSFNREKLSAARDLFNKSASLLDFNPDTNEYIRNRVKS
PNRTEGSTVSRRFKVDNHLDTX
>IdupIR14
MNFRRIGIVLCVLVTFSLAKDNITLGIFFNKDENGITYWEALEDILDERLDIIPEVAEK
DENDVFEKDITEAKRRYCNFISETKSRIVATLGPRSSFVSPILESISHHLGIPYFITTWRS
KSEQLPNVFNVYPEADLLARGLAKIIESFNWPEFVVLYEDDQGLLRLQEVLKIQQFEE
GSLKNGMTLEKLEIGGDNRHIFKKIRSTSINKIVLDCHVDRIKDYFNQANQVGLMSDF
SRSFFLISLDAHTLDLESLKSQTNITSIRLLDPATVN
>IdupIR15

ISAQAALMYDAVFVVVEAFNKILRKKPDNFKNFNPRPGPRQFFTNGSKILDCNPSGG
WVTPWEHGDKIARYLRKVEIEGLTGEIRFSEEGRRQNYTLHVVEMTVNSAMVKVAE
WTDEAGFNPVAAKYTRLKPAQHIERNKTYVVTTIEEEPYIMVRKEEPGELLIGNNQF
EGYCKDLADLIARELKINYELRIVKDGKYGAENNEVKGGWDGMVGELVRNEADLA
IAPITITSERERVIDFSKPFMSLGISIMIKKPMKQKPGVFSFLNPLSQEIWISVVFAFVGV
SIVLFIVSRFSPYEWRILHHSDEPIRHPPHLHTNSGGTMANDFSLLNSLWFSLAAFMQ
QGGDISPRSISGRIVGACWWFFTLIIISSYTANLAAFLTVERMVAPINSPEDLASQTEVE
YGTLIGGATWEFFKRSQITLYSRMWEFMNSRKHVFVKSYDEGIRRVRQSKGKYALLI
ESPKNDYTNEREPCDTMKVGRNFDAKGFGVATPLGSPLRDAVNLAVLKLKENGELT
KLKNKWWYDRTECLKDKQDSMRNELSLSNVAGVFYILIGGLFVAMGVAALEFCYK
SHLEAKRAKIPISDALKNKARLTLGVGRDFDNGRYYTPANQIGPTTDNETPHSNTHTS
QYTPSCTVX
>IdupIR16
MAVAWWVVVLVQLVNASKVSCRIPLPLSEDYEMEMVKISSGGDLVTASHDIIKGLP
ENKVVIGFHKTDENFIKYLLKKLGESEISVLYFNLSATDVQDKYFDYLAEQTENHLP
VTSMFFGTPRFYQHILIQIYAQNSIRRNLIYIFNWGANFPDVYFRQNMHYAMRIVAIT
NPRYGTFRINYNQAVSNMEHHLKLINWWNEDTGLFHHPTLPQKVSVFKNFHKKVLQ
IPVLHKPPWFFVRYNLSDNRSLDVFGGRDDRILRVLSNKLNFRYNYYDPPERILGSSE
NQFFNGVIGQVAQRKADLFIGDMAITYERSKVVEFSFITLADSGAFVTHAPSRLNEAL
ALLRPFQWQVWPAIGITCVIVGPMLYALIALPNAWQPRFLVRSHARLFFDCTWFTITI
LLKQTGKEISSTHKSRLFIMVLLFSATYVITDMYSANLTSLLARPGREKAINNLYQLK
AVMESTNFKLFVEKHSPSFGLLKNDTGIYGQIWEIMERSQNKYVMDSVEEGVKLVN
NSRNVAVMAGRETLFFDIQRFGSMNFHLSEKLNTAYSAIALQPGCPYIEEINKILMAIF
EGGIISKMTENEYENLGKQQKIPDAKIRENEAKTTAVERVKAAEDDKLKPISLKMLQ
GSFYILFIGNAFSGLVLINEIALYKYRLKYRRKKKGRCQTVKKAWKRVKGRMRNAK
NNVSRMYRNFMHDAVTMTLEYIEX
>IdupIR17
GFLEEILRLRLVRNWLLDPNDYVEEFHKSGIPTIIFNDFDDYPELSNDLNVCFLFVKNS
FPDMSMLWKRRLIHIIVGDFKLEDMKQFSEFIFREYWMTDLSFTNLSFAQVWRYNVF
TKSMLEDSTPNLVFQSFITNMALYPLKVVMFQNIASIWNGVQFVGRDGLMLSEVTK
RLNATPVYVKTHQGNFGTVLPNKTLTGILGFLSNQKAHLSMNTRILLGDAEKYVDFT
YPHDQDNIILALPIEYVSRDEAALQVLPIWQQCISFSVAILFAIYLKCTTRLPLSGVFIV
SVQLLLGSGVQLATFKSSRILLMSVLVYFFYQTTLYQSNLASVLSLGITKPQLVTMEQ
VSETSLTFLAHNVTFYPLEMVKHSTDTQSVLDVLSRTTFIPSAHFNQTLLKCPSSVGLI
SVSRVFDNFADHIKDENGYKKKCYYKVKEYFTSVKQTFSLTKGSPLLSRINKIIRAVI
EAGLQNHWEQMNWFSYEDRKVNSPTKGSLKELEWIFLGMVIGLMVSTGVFLYELK
AX
>IdupIR18
MDGLVGDLIEKKIDFGLSPLFVKKDRADYISYGRKTWNLRAAFILRNPKSRKSYQIFI
RPLDTKVWICVIALSCIAVLVQTLSYKLDWKLSNWTSKERKHIDFTWGFSAVSTCGA
FCQQGIIAFPSLLSGRIAAYMTLLLGLLTYQFYSASLVSFLLNVPTTVITTVQGILDNDF
DIGCEDVLYDKDFLKYATDNITKQVLKRLSTSNNNRTSFLKPAEGLNLVKLGKYAFH
VELVSGYQFIEKEFDETTICELKEVPMFATQQMHANYQKGSPLKDVLDTCLHRLGEN
GVMQRELRFWHPRKPECVRSGNTIVINTGLEEFYPALCVLIAGIVLSLEILGIEYFLYQ
SYQTSLLPFVNX
>IdupIR19
MLAKKVNIVNFLTTGHGKMGVVLDGSCREAQNLTQVAGRHNFFNLKYYWLIFSTK
TNLENWFENVSMNVESNIYVAHQDSATNYVIYEFYNPASTKGGVLHKTEAGYFNKS
SKYLIKNIDDGKFWKRRNLTGVTFNTPIVLQEPYSIPVEDYLRTDKHREINTYNRYQY

RLMKFCQQNYNFSIKLTVLTSFGYKQEDGTMDGLVGELQMHRADFGLSPLFVHEDR
VEVMTYGRHTYSLNSAFVFIIPRSKSNFYIFLRPLSSSVX
>IdupIR20
MGIVIGLITLLVEHLFFKYILPILREKPKESMWRSRNIMFFSQKLYRFINCVELVSPHH
AARELVHTIRQGQITSLFQKSIKRKEHEQRRRRKSKAQFFEMIQEIRSNMRRQQQQEE
TQPHLEALTEVDSEYNSPEEKKSRLSPNILKRTFLRSSPKSDNEGKVKSPTGLFGKQLF
SPRSKKAKSSNSLNVRRFSTDSVFNSETPADRSASIGRRLSKDASSFLNTSPPDINSRRS
SYLDIISTGSKLSGKSPVLSIENISDCGSAKSAEPIRKLSDCESIGKKLATLPKYQGSFD
KTKLQPQYSLTLGKSDETLTNTEKPEPVAAAKSFNSIDKLDQKSNLSDDEIAKSRKNII
NQLQTELKAKTKKQYSVDSSRPAYSPNPQIRIQVEDIDGMEKVTPKRNPPLAKTTRT
KTRHQSLGDPTQNPLISERSTRKLQEASKSLDSAESSSSRVKRSRSRSRHLDEDDLPPA
PPPPNCSPRNSDGKSPLDRLSKDDLVKLWRSSESELRSHLLKAIRDKEEPTDPTX
>IdupIR21
NPNNRANEAWRKLIYETKIMSKNKHPVFYSTAEGLKLVKLSYFALHVEYTTATDVIL
ATFTNEEMCAVRFIESIYKEDVPYISCPVNSTYAEYLLIGSVCCVINSVNGNGFCFQVS
QTIRIWTSFQRIQKKIFKITQVYWX
>IdupIR22
MMNFPLTCIMILVLGFVTAFEKTYLPSDKVAFLNDFLRQSKDKHAIIEDHMCWDHDE
SIILQKLLSKLRIRYAKPLNFSIPQSFHSTYLADSTCPGFQELIGDVIQTGLAKYPNKWL
IFGNTTSITNPGYYFPVNTYILVVNSNYGILSVETLYKLKNSSEYHVSRVAEWSKSTG
FIFFNQLNLIKNRTNFFKMPLTVSYVVTNNDSFQHESLLNNREKHIDKPTKINYLLYE
HLVDIWNMTHEKVLQREWGMEFHPEKLYYKGMLGDLCHDRADTAGTVQFTPTER
LKYFKFLVSTTKEMKIKFVFRAPPLPYSSNLFALPFDGKVWIGCATVLVFCCLVIWITI
KWEIDNPTFIAEREMYEENAPDFFDIVLMQIGVVCQISYSFKPRSTAAKIATLSLLVGF
VYIYNAFCARIVILLQSTANNLNNYKDLYYSKIDMGVEEAPYNIYYFSNPNNRANEA
WRKLIYETKIMSKNKHPVFYSTAEGLKLVKLSYFALHVEYTTATDVILATFTNEEMC
AVRFIESIYKEDVPYISCPVNSTYAEYLLIGFHRLFESGLHSRESRRKFSKLPKCTGRNS
IFVSVGMIECYFAFELFLIGVILSLVIFSMELVCYTYLKKRKNVQTVVLTPTNX
>IdupIR23
MCCIIMKVLSVLVIILINSVIINGEVLPLNYNNNLELLKTFLSSILPEHEYVCFFEEKENE
LANIIPRIFAVYTANISMLSNTIIQCNLYIFNIEMSHFKEVISLVPPHKTVIIFPFGLDGK
MQNYIIEDTYEHAVPAILVNIKENNVIELFYVMDNKTKYLSLNDTKSQYHIKEQQWS
PEKFLSKAGRPIVVTTFHCPPFVEVNNEEKSYEGFEYKIVEDIIKDWPVKYNIIENDRK
DVLINKFLLAIRTIQEEKSDIAFCFLWQRALMERNVDYSSAMFPTCVTFLVHKPTLLK
SYTFLFQAFHDLLTFVVFALASAFWELIFKVVVTIKGRSPSQSLSVRLTFVCSTIFYFLF
FSYYSAELTVISSFPRFSGDYIKTFTDMVDKKIQWVEPQNDIQTWLKNTKDNICVGIA
ENFRTESNRKIINLKLQGGKFGFLVKRFASNLLSGVEDLDDYGKTYLRALPGCLATF
YSSIGFQKNSPFTKYFNEKLYRYFDSGLVDYWEQIVSRKPAYSYMENFKSLYVGQIT
TKKFDLNKLSGIFYLLVLGYSLSVCCFVCEYFKLSHITNSNASNAX
>IdupIR24
MGLRAVWWFSTIYFMLANLTRSEHPTWLWNKSRNSFYLKQQSDFTYFNSRRVRTQI
FLDISNLTIDVLVHKLDPYLEMNSNTLGGFLGDTWNILRETLRFKSNFRVVRFKKGCE
LIQQNKADVFLGALVYTEETMDMIFSQPYFRNWYHLYFKLPEGKPADYLHFKTINA
KLWLYILIFFKLLTVSLWFSCVILHKLVPKIERSMLSITTFYLAVVASFLNTGYDLKLR
SFTARLVILLALIFGVLCYYALSASLVASLAVLDRPMPFSNLNEISVKKTHSLCMRNIT
FVYDSFTDSKHTLLPQWKSLVNNDKCPNDMVDWKSIPNALCQDKVAILENRIIMSW
VLQRVTCQVTLLPQRYFDTGNYIVFSKKFGQQKSISIMMLRMRSAGISRKLETKWIR
NHLTTQPTTGANQITFSHITGLLTIYSLMIGISLCVLCIEIVFHYYYNKHX
>IdupIR25

MNNLVVIVPKPTIVDRWSYVVWNFCTKSIVLMVVMSCLFAQLDKLFTNDHQLNLNF
FFFSTLRVPLQIKRKSTIKTAWLLFVLFFGIFLDNSFLNIILTDKYQTEIKTSEDLARSKL
KVYAFHFKNYDPKVIGLPYSFVTVTKSKWKNLVFKNDGTSAFLASFVEIDRIMKYFA
RQHIDFNYKYLQDVCLPKFASLVVKARSPYLKKFGMLAAKVMEFGVSGTTSYKIDEI
LAENVRITYQHFIGVFVILIVGLTISSLVFLAELYWRKLRGNQX
>IdupIR26
MSQSNTNNVCFYFRKLADISRSTRGVVVLCSDSKSAIRILDDAKRLNMMDGHFVWI
WIDTASSINIKNSSEEDAIDRSKRDSDEIILDENAFERREKRSAFLYSDINDMHINYLLR
NDNFLLLNRNIPSVASSKVNRKASQASSRISSSLKSERKSDL
>IdupIR27
INLKTRGVEYNMLFDKQLFLILIFSYLFTFSIKNTFGLTKLNRCRNEGIQNLDKAIRKP
VTNRSLATNQFKNQRNKRYVNNDFGGGKKLNFTKIFYYTNSSSPSIISMAPFTPIEDKI
AQLNFLLLTEVMKMTNSVLFPINNINFDILGTPVVLTTSTFSSYNVFGKMATVERKFIF
KAVPPSYISNVFVLPFDTHVWHCCFGLTGVIFIVVYIITFWESKVHVFNKNFEPSRSLR
AHPVDIVLMEVSCTAQQGFEAEPRSNSGRIAFIWTLVALMFLCHSFAANIVAILQSTS
ESLNTFESIVQSRISIGFEKDIPMNYFYQLTNSSPSEDNFDKLRNPWFFTLKQGVSTLQ
QEFFAFYANSFDVYEYINLLFSEDEKCTLREIVLKPTKAEYWSLVKKDFPYNEMLKV
SMIRLSERGILSRESRKLFSKKPYCEGEVGAFASVGFMESYGAFLIMLSGLLLSCILFFI
ERLVIYVRKPX
>IdupIR28
VRVDTIIWPGGDLSVAAVSTRARTVFRVVTALAPPFVMESELDEDGQCLRGLPCYRV
LTSDKDNLTLVFSNIQRREEEEEEEEEEEEQLEEYEDYPRYEHDMDEETFFPFQNFKYR
TNCCYGLSMDLLENIAQELEFDFRLYIVADGFFGSRIPTVKSNRVKRDVGKKFLSYRF
QDGVLKTHRITENVHPVNSK
>IdupIR29
MLAGFMSLSVLTSLVIAKFPSIGANCFLPHHAELYRQHFKLVKHIRITRVYDRQEYNS
EDLLEGLLGLNSHFLRKTIWMDHQHFITENSGSNPVTALPILSTLNISEEEVKGEFEEK
LQQLDVHLARVFIGQSPEVLLNYFQSVDPKIVTYSARCLTVVLFSTSSATTTKSTILLL
TFLWAKLSFVNVLVHFPCSLKLQEYVFTYKPFYRNGDFCGQSRRYHYTDVLKYPQL
LVNDVKNLNYCPLVLSLFQRYPTATATPPNFLLGSRVYEKIPLTSKFYGADGVAMSV
LSEYMNFSISLSTSEESGYYGTVTKSGAPTGSLKATVERSIDLQGNSRFMKPYGIEGY
DLTYIFDFDKVCVVVP
>IdupIR30
FNTTLTHSWFGNTSSGEDGGLAKLLWDDVIDISSAGCIIRLLGTERIDFYEFIVPYYKF
RSCFYFRNPGVVKPNFKEVLKPFTRQTHLVDKWRSGIIGRNSHWYGSIFIVIAVFAQQ
GLDNIPSRISSRIIFLNLLICSVLLYNYYTSSLVSSLISTEPQVLKTIRELFESRLRVGIEY
QPYTITYMLDRVKKDHWLDMLNKTKVYAHQVPNFFTAEEGVDMVHQGGFAFHAE
SITTYPLIAHTFEQDAICDLAEIVLINSDTSLMTQKKSQYKKLFEISLRKMWQSGIMKK
LYKTWVASKPECLSSTRMITVGVNDLFLPYFLLVLGVAGSAIILLGEMIWIKAGARIK
RCCSFYSSNDKQX
>IdupIR31
MFCVNYLSHHVGNSLNKRIKLGLFLYGTLLGNGNRNQLPHSSTLRIYFGHLLFLFMV
IGLYVQGALVSVLSGSLFEPPVMNVHQLAASKIPFKTTELFKSMIKLHPGLSTNRELIE
LVNRVQIINETILLNTLDDLIKRKDYSTISIKALIMLRPNLKKRFSYFPLMRLFVSFGM
DKNSFHSPQFSDWILQGLETGFMEKYKRLYQFSMALKWNVELEEKVFVLTVEQVQP
AVKLLLVLHFVASLVFIIEVICYKCNTRVNX
>IdupIR32
DKSIIKDVFETLIKYEVLNAVLLIADPENKEKLNLYGLVPFNNGYCSKIVTESYITLLD
YCINGSYMIGNDWYGHKVPKSFRSCELKVAYAEVAPYVINMKGKTYLRPSEFEHHG
IEISLITNIFSNMNITLKFFWSDIRN

>IdupIR33
MLKETTEQLVGNDRYEGFCIDVIQELSTLLGFNYTFVVQEDGKNGNLNRATGQWDG
VISQVIQGNADLAITDLTITSERENAVDFTMPFMNLGISILYKKPEPVPPSLFMFTSPFS
TSVWLMLGVAYIFVSISIFIMGRLSPTEWNNPYPCVEEPDYYVNQFSIRNSLWYTIGG
LLQQGSELAPISISTRTASGFWWFFVLIMVASYTANLAAFLTVETLVTPFKNIDDLAK
QTEIKYGAKKQGATENFFRDSNVSTHRKVYNYMKQHPEYMTKENEEGVKRVETEN
YAYLMESTTIEYVTQRHCSLAQVGGLLDDKGYGIAMKKDSPYRNDLSTAIIKLQETG
VLTRLKIKWWKEKRGGSTCSAKSGESEAQALSLQNVGGVFLVLFLGAALALLGSFL
EMVGHVYRQSRRRKESFLHQLKAELGFFVQFKRNIKQAASFESEEKX
>IdupIR34
MKLTMFAFNLLVASVVSGGPAWKETQRGSGLRIGEKSGRNNTSSRSSGIKLGSNARI
LRSTTTTTVSPEEDHLHPPTSSSSDGNKRITHLNIGIVVPYKSFGVRDYTKAITTTKSLI
ARKLKLFKTHDIQVHIVMKQMTPSPTAILKSLCKEFLNFNVSSILYLMNYEQYGRSTA
SAQYFLQLAGYLGIPVIAWNADNSGLERRASQSSLQLQLAPSLEHQTAAMLSILERY
KWHQFSVVTSLIAGHDDFIQAVRERVSAMQDRFKFTILNAVLVANKGDLAALVNSE
ARVMLLYCTKEEAIDILSAATDLHLTGENYVWVVTQSVIETAVQAPYQFPVGMLGV
HFDTSSSSLVNEITAAIKVYAYGVEDFLADPVNKHRSLTTHLSCEGEGAARWDTGDR
FFKYLRNVSVEAEQGRPNLEFTQDGVLKAAELKIMNLRPGVSKQLIWEEIGVWKSW
QKEGLDIKDIVWPGNSHTPPQGVPEKFHLKITFLEEPPYIKLAPPDPVTGKCSMDRGV
LCRVASDEAITEVLDPSEAHRNGSYYQCCSGFCIDLLQKFSEELGFTYELVRVEDGK
WGTNINGKWNGLIADLVNRKTDMVLTSLTINAEREAVVDFSVPFMETGIAVVVAKR
TGIISPTAFLEPFDAASWMLVGIVAIHAATFTIFLFEWLSPSGFNMKLAFNNGVSSNAH
RFSLFRTYWLVWAVLFQAAVHVDSPRGFTSRFMTNVWAMFAVVFLAIYTANLAAF
MITREEFFEFTGIDDHRLCRPFSHKPSIKFGTIPWSHTDSTLSKYFKDMHSYMKQFNK
TTVMNGVDAVLSGDMDAFIYDGTVLDYLTSQDEDCRLLTVGSWYAMTGYGLAFPR
NSKYLKMFNQRLLDFRENGDLERLRRYWMTGACRPGKQEHKSSDPLALEQFLSAFL
LLMSGILIAALLLFLEHLYFKYIRKHLAKSDRGGCCALISLSMGKSLTFRGAVYEAQD
IIRNHRCRDPICDTHLWKVKRELDVAQMRVKQLEKEMDMHGIKPPPCKRIVVSGEQ
AKARLRSIEPADIGSNSDLNGPTTTEIAEMETVLX
>IdupIR35
MIIRGFFCLFLFQVVQLQTTQNINVIYVNEEGNTVADKAIDVAMNYIKKTSKLGLSVD
MKRVVGNRSDSQNILDSLCSTYQKMLDSNSPPHLVLDATRAGLASETVKSFTAALGI
PTVSGSYGQQGDLRQWRNLQQNEEEYLVQISPPGDLLPEMIRTLVSNQNITNAAIIFD
DTFVMDHKYKALLQNIPTRHLIDEISTDVSKIPDQLEKLDKLDLKNFFVLGSLETVQV
VLESAEKKNLFNRKFAWHVLTKDPGDIKATVKNATVLFARPIVNPAYQDRLRNIQTT
FQLSNVVPEIDAAFYFDLALKAFLAVKEMLLDGSWKKNNVTNYVTCDDYEPKISPK
RFNLNLRSYLQKESSEPPTFGPFNIATNGLSYMEFAASLTVVYVRSGTSDKSRALGT
WQAGFDNNLTLFNPKDMQNFTADVVYKVVTVEQKPFIYKDPTAKKGFKGYCIDLID
KIAEILKFDYEIEAVADGMFGNMDEQGNWNGIIKDLIEKKADIGLGSLSVMAERENVI
DFTVPYYDLVGITILMKLPETPTSLFKFLTVLENEVWLCILAAYFFTSFLMWVFDKWS
PYSYQNNREKYKDDEEKREFNLKECLWFCMTSLTPQGGGEAPKNLSGRLVAATWW
LFGFIIIASYTANLAAFLTVSRLDTPIESLDDLSKQYKIQYAPVNGSSTMTYFQRMADI
EARFYEIWKDMSLNDSLTDVERAKLAVWDYPVSDKYTKMWQAMKEATLPPDLET
AVERVRKSKSSSEGFAYLGDATDIKYIHMTSCDFVVVGEEFSRKPYAIAVQQGSPLK
DQFNSAILQLLNRRELERLKEQWWNRNEESKQCETSDDQQDGISIQNIGGVFIVIFVGI
GLACVTLAFEYWWYKYRKNSNITNVIVSDPKHRRVAGFPKDVGGKANDEGAFRPG
KLYVKPKYX
>IdupIR36
MSNVSCDKEQPWDGGLSLINYINSVEFKGLSGPVEFKEGKRIKFKLDLLKLKQHSLV
KVGEWHPGMNVNITDRNAFFDHGTMNVTLVVTTILEQPYVMLKSYPHPHDHELYE

GFCIDLLKELATMVGFEYRIELVPDGKYGAIDLETGEWNGIVRQLMDKKADLAVGS
MTTNYARESVIDFTKPFMNLGISILFKVPTDKESAFFTFLDPLAVKIWLATLGAFFMA
GFTIYALAKFTPCEWVDLQPWKADNNKKLVNKMNMSNAFWFVA
>IdupIR37
MGLVQILIPILASLCFDRNCTDEMMKAKAESRIKNETFVVTTLQNGPLSGYLEVNQT
YIGTGVAFEVFNILQKEYGFHYVLQPPKVNTFEGEGGAKQMLLDGYADIVVAFLPEK
QGDPGISYSRPLDIAEWKVLMKRPKESATGTX
>IdupIR38
MLGLLMVLGQVISCGLCIVVQPKGPTHRAVAVDCLVKVSEDAYNFLQTKNALNRHF
LLTYSITGNLSTPAFEIQQTLLQRFHRQKVWSVEIIEGDAKLKFTAHSENDFRRPVMT
VLSDLNVLVCDSLENLDIKLNYLSKAESFNSEAFFIVYYSTKTVNAQNEAIAKKAFKV
IFAYSIRYVVVVIPKTLTSFEHYIFKMEAKEGVPCFAFSSYTLTMVHLCAPYNSNHHG
KNIFSETVIRDYNKCYAPVQALPYPPFVIEQNLGVEIDILKLVELMLNLTFIIELYPNYT
IHLGEKTPNGTWTDFLEPIFSDWQLGVGSIPPGSELTEDFTFSVPYSWNQNVYVVPIA
ALVPSWRILMAIFTVPMWGICFAGLVGFAGGCYLVKNQHETRIFQTFGGCFLVAFQL
VLAHPVHTHPKGDLVRVFFEGFAILCIILNCVYTCSLIYFLQNPVREHQISTQEEIISSG
LALGGTPAYKEMFNTSTDLSVRALAESYVTVDEQLNSESYWLNMVAQQRNISTIGV
KANLIFLMQTQNGLVSDRHGQPKIFLLSKPLRSQPIGILMRKENFLEKPINRAIQRLILE
AGFIEIILKGYERPSGSMVPEEGGHYEGDADSDQWVSALSTRNLEGAYALYAMGVV
SGVFVFILELLYHYSFKLYQFRFKAX
>IdupIR39
IDTVGLISIIKFNVWLLLVLLVSITTSIILLLTKRTKLELEEFQKLVATFVTVLLASLNFY
THLLPRTSEVRVVFSVILIFALIFNAAFTTYLTSVLAREVTTTEKYQTVNDIKKYNLSII
RAPNSERAFANNQ
>IdupIR40
MILEAVVDYLNGTHVTSFYSNFGLSHDENDLFQYGSMMGDVQRGKADVAGVSGW
PDRHRLHFLRFFGPTTPDTIRFVYRAPPISLVRNMYLLPFDLLSWYCIGGILMVGVAL
LFAVLKQEKRFSGRTNGSFVDVLMILLSALLQKSWDLHFTTYSGRLSLAITYLSFLVL
FNAYSASIIVFLQGTSDYPKTFSDLYSNNFELGVHNVNYNKFYFMVSVPLVKFSFRX
>IdupIR41
NSNDRTDEYWREKILKEKITRYGEDRFFLSLDQGVDKMQQGMYAFQSQVSAAHYVI
NKKFTQDQKCTIRYVKTVFRVDKLVYLGVRRNSSYLEHYKIGTRRMFEHGLQRRIY
LRLFNMKLSCEGNISHFNGIGTVEIRFIVQFFVAGIGTSLLILLLEILVNKTNLLX
>IdupIR42
MIVSVKNMHSINVLDHLVDNYNRMGVILDGDCHYSTELLLRCRKYKVFDTKHYWL
VLYNNSDYLNLFRNTNLNVDSDVKVVYPSENNDTYTIDDVYNQAFEKGGDLKSFIV
GLFTREKGYQVNEAVNKYVVRRNLTGIQFDTLIVLSESYEGPLIEYLRSDQSIQVDTL
NRFQARLLQYCQNYYNFSVFITNVTNSWGYYQPDGTMDGVVGTLARKKIDFGHSPL
VAKSERAKFISFGKGTWHLRMVFVLRNPNNKRSFEIFTRPFSLEVWICVITLALICVLS
QNVSFRLDKSHRKDVDSSWSFSALCTLGAFCQQGITAVPNCLSGRTSGFIILCLGLLA
FQFYSAVLVSFLLNVPVTVISTIQGILHSDFGLGYENVKYARSLLQQATNNQSKEIFRR
VSANNNSGFLTRDEGLNLVKKGHFAFHVELVTGYPFIDKNFDLAMICELKEIPLFPPM
YMYSGYQKWSPFKDVIDVCFQRFEEYGVMRRELLFWHPMKPQCVRTPSMIPIHTSL
EDFYPPLVIWSVGVVLSLQILVFEILWWRRETRLKEPYAYTNX
>IdupIR43
MFAGLLISFGLFWARIQGEIFPSLITTNASMVVVVDREYLAENYDKIRIGIEEYLVFGK
REILKHTGVNVLVSSWPRINVKRDLSIILSITSCEETWDLFKAAESEHLLHVAISEQDC
ARLPQRSAITIPIIERGQETPQLLLDLRTTGVYTWKNMVIIYDGSISNDLLIRVIKSITKR
VSNVGASGISMISLEAKAFANSSGIEDILSKTFSQLVWRKLSKNFIVVAKYELVEKIM
QYAKQQNLVDTNTQWLYVISNSNDTIRNMMRFKAMLREGDNVAFIYNSTSTKDICM

GGIECHINESLAAILHSLDRAYVEESEMSRQYSDEEWEAIRPSKLDRKTFLLSQVQKY
LFEHGNCDNCTKWKFASGETWGKEYQTHEDFSTVDIIPVGSWRPSDGPTMTDALFP
HVAHGFRGSNLPLVSFHNPPWQILKTNSSGDVVEFKGIVFDIIQELAKNLNFTYTVQV
IQRLISNNVTSKAGNKTDDDFLGILGTSSVSTFKVPQAILDIVHNKSAIMGACAFTVTE
QNKKIINFTDAISIQPYTLLAARPRELSRALLFISPFGGDTWLCLSLTILSMGPILFYIHK
YSPVYEYKGIRKKGGLATVQNCIWYMYGALLQQGGMHLPYADSARIIVGAWWLVV
LVIGTTYCGNLVAYLTFPKMEVAISTLEDLVSRKQSVTWSYAENSYFEAVLKDTQDY
TYKTIFQNAKQISNKRYMLEEIKAGRHVYMDWKIKLQYVIKQAFLEDDECSFSLGSD
NFFEEHIAFIVAPDTPYLSKINEEIKRLHQVGLIQKWLEDYLPKKDRCWKKQNTLEVN
NHTVNLDDMQGSFFVLLIGFCIALLVILVEKGWKQTMTNRKKKIVVNPFALX
>IdupIR44
DRDRDSNPTTFNIGGVLSSNASKGLFKETIDHLNFDSSFVPKGVTYYNTAILMDANPI
RTALNVCKYLISKQVYAVVVSHPLTGDLSPAAVSYTSGFYHIPVIGISSRDAAFSDKV
VLLHSPKSX
>IdupIR45
FNPFEDVSTPLYQRLAKKISRKIAKVDDNYTSMEIAAVFHIAGIERYLDGKILIHARFS
DKDGHSQLHIIPECPNSYFLSFIVPLGSPYLKVVNRFLIKVNEVGLASKWHQDFADGF
VYDARIRRLDDDTDEDNNGFKPFDWEDLKCIMAIWLIGLVIAFAVLILEIFCFQIX
>IdupIR46
MMEPLTFVVVAIWIVVASGGLPPVIRIGAIFTEDQKNSSSELAFKYAVYKINKERDILP
NTTLVYDIQYVPRDDSFRTSKKACRQMEFGVQAIFGPSDPILGAHIQSICEALDVPHL
EARIDFEPASKQLSINLHPSQENMNRAFKDLMSFLNWTKVAIIYEEDYGRELGLFKLQ
DLVKAPGVSKTEMYIRQASPASYRQVLREVRQKDIYKLIIDTNPRNIQQFFRAVLQLQ
MNDYRYHYMFTTFDLETFDLEDFKYNSVNITAFRLVDVDHPKVQDILATMEKFQPIG
HAILNRSGIIQAEPALMYDSVYVFAKGLAAMDSGYSIKPTNLSCDVEKPWDDGLSLY
NYLDSVTNLQGLTGNLEFQEGKRTNFKVDLLKLKKEEVRKVGTWTPNEGINITDPN
AFYDQHAPNITLIVMTREERPYVMVKDDQNLTGNARYEGFCIDLLKWIAGQVGFQY
SIKLVPDHMYGVYDPETKQWNGIVRELMEKRADLAVASMTINYARESVIDFTKPFM
NLGIGILFKIPTSQPTRLFSFMNPLAVEIWIYVLAAYLLVSFTLFVMARFSPYEWNNPH
PCHQDSDIVENQFSVSNSFWFITGTFLRQGSGLNPKATSTRIVGGIWWFFTLIIISSYTA
NLAAFLTVERMITPIESAQDLAEQTEISYGTLEGGSTMTFFRDSKIGIYQKMWRFMES
KKPPVFVKSYEEGITRVLQGNYAFLMESTMLDYAVQRDCNLTQIGGLLDSKGYGIAT
PKGSPWRDKISLAILELQEKGVIQILYDKWWKNTGDVCTRDDKSKESKANALGVENI
GGVFVVLLCGLALAILVAILEFCWNSKKNAQTDRQSLCSEMAEELRFAMRCHGSRQ
RPALRRSCTRCSPATTYVPAALDLPHLNGEGVILPMMDLKKSPISYDIDTX
>IdupIR47
MLTPLTLIFHLQIVVTTSEIAPIIARHTKVNNCIELTLKTQGLALILPTPHSINKDEFNYL
LKAISSCQIPVFILNEPYELNKWRWYTVLSTDTLTGQMEHVRLPKFYQTKLVNHHNY
TELQLIPPQFKHTNKSFCLTMFESAMVVRNGDLFTGINVEIITLLFEYLRIPYEFSTPKD
GHTFGDEDNMTGAIGQVIRGEAEMVVNTQILVLKRHLVDYTYPFLRDDVIIVVPRSY
ASHQTRFYLKMVAILVAVFSTAVFAYFKTFSRLTLSEITLVFVMIFTFKPIRIYHLKYF
RIFYATVLVGAFFTCTLAGLQIFSRQTAQRSLERIKTLNDFAKSTTQIFARPGIVALAK
DALKNHPDYSKIVKRIQTVSFGPDEFTYEWDVMVNCSPRAIICRKGTFALYTAKHSK
EMCYYPLVEPLMPSLNSFVINYGNVYYDKINTGLGLLVEAGLIFKPIKLNEIHQQPIA
YTADYFIGRKTWLIFGLAMVVAIFVCIGEVIWYRWNTHNKX
>IdupIR48
MLPTFILHIVTMILFGLFVLYAGSVGCKMKFALPGPLSTNLSAFHSCLRRFSQSQPVW
SWVILHEPDGNQPFDFILQTIYKVNVPVYNYNNLEANKRGFTWFPAGPLRASTLAQE
SKVFFLSKSKFHINLMGNGSGSFRTGDKILFTNYNQGSRISFEKVINFFRIKTSTTGLTR
YNLLPIVSFLFINETGEADPVTYIPIDRRDMRKVQKLHLSCSQIFSNSIDKLIFEKDYFT

VAAFQTANAMKSGNEFFGKDAIIAKILIGCTGFPYVYSAPSDGMTYGSVVGEYSGVY
GAVARGEAQMALNSRLFRTEIITDNVVEYTYPYHRNDLVVLIPVEHTTSHHLFYFTT
YSYVFSTAIIVCLYLYINRMGQNKQSFIEILLIYLLICTGKPIKEFERRYFRIIYASMMV
VAFYGYTLFNIQIISALALHSCDLGVKSIKQFAQTDYKIYSIPTLIGILNQSLQNVDESS
LILSRIQTAQSEQESTRTIKDIIINCSNHAIICDSSTAEYFKDLSYLQNKEFCFETVSEAIV
PGLVTFVLSYRSPFLDKFNEALARIIEGGLVANYTKNTPSLTGKLKNDFEPYGMKKPR
EKFPWKVYVVGISLAFIAFIGEILFMFTCTKKVEX
>IdupIR49
MNLLTKLCFFIVLYLLTKSEPIVVQSHLAEDELHQCIGSIFKTFNPTGFLEVIVTNLEEK
LLDNNIPLTRIRFSDFQNYTEAFQIWKKVRLYILDFRPPNHYITIRRILDLIPRSYLQNQ
FYFIVRTTEQVPFEFLAENYIDNVLVFTETGSIFTYFPYKFGNIKNPDLEAIQIGSCRNT
NDVSITKKETKNWKNSTLRAILRVTTPYVNGDGNGVEEKLLQLFQNQLKFKMSYEY
LEFGQTFAPYLKQKQQIKTQEADLFGGHLTISEDDAWNFSITPAYLQDTMRFVCPRPL
NRPMWKRLLKIFPFNFWIIFSILMTIQTALAKIFLNIRQEKLMLAMFQILVESPTVEAET
KITYKRITLASWFLSFMIMSTIFKNGLLIITETIRKEPAIETLTDIIQSKLPIYPTLNLSFYY
HSEKEIKDFDSITIRRCQNYLDCLNDIAKNQKSITTGGREILRYYTVPKYFINNGELLV
HIADEAIYSFHISFLFSRGHPLYVQCSKIISYSLASGWFVNVFSRLRYELFLKTYQKYRI
SSKTFTLNDFKDISILWFSGVLIASLALIGEHVWYYTRKKYCARYTFSQX
>IdupIR50
MNISKWLFPYLVLLKVINNQANSPVKTCIEYLLNTIQPQWMSQILTNFEIGLKYPVVI
VHNLTEFQKSPLANVRFDVVIMNTSLLSVEDFLTWNRQKEYFNSKATYILLSQNISSK
PFQILANHFLKSAYILDGNLSIYSYHPYRYENVDKPDTTPVKIGECYSHYIKVPEKYN
VLWRNTTVKVGYRVVEPYTTENGDGLENMFLSLVQQILKTQLIGIHASGQFIRDETV
SKQGIDMLKKGEADLLIGYNSPTEITVKHFDVSDLYYYDVLTWVYPNSQKMSHWKR
IFHIFSGELWFFLLFAIIIWISLVYYIEKKICLCQLFFVVYQVLFENAVSKSLFKNRKAL
KLLFSAWIMAFLIISTMFKNSLLIILGTEKKLPEFLTIQDLLDLNFNISSHSDLSTFYIDN
MTPITDIYQIENCSSVLVCVNRTAFGKNSITTAPRSIVWYNWIPKYFITDYGEVLVKAS
STTIKPIYINIIFRKGYPLYEQINYIIENMRDHGWFNYVYLEMRLRIKRATNHLVFEAN
KIGMSKFGFVFILWCIGLSMAAIVFIFELALHATLHLTLX
>IdupIR51
MQLLLFLLSLHYLAASQRQSFLSVNASGSVITVSLFKATFKNCTSLCYIHEKDIENRDI
VLKHIKIPILRVEFNGKVVTTDLVNQTVCQGYILNIKNLQAFKMLLQSRDPSVEFIKS
HRRMAILYNGKAPVRFQELLGLYAIDVMEFEIYYNEHMEFMGCRVVRLYDDKVLFL
WEHPQPDYYIDPENFGYRSWDMRKFFNTTNYKFRFSVFNCEPFIYIQDGKLLGGTEF
NLAQTITRGFPVEFVFTNEKRNPWSRVLQLVETRQVDMAGCSQFIKNLWKKHVDFT
LNQNQICNTFLVPKAEPITSFTFLIEPFHIFVWFVLSLFVLMGYVIFIIRHRRVMKQLRR
NPQYSLVFLSIVRLYSFGNVHIFWKATTDMPLRFCFVCFLIHSLLMSTYYNAGLSSKL
AIPSLTRQINTLQDMVAYGITFQECTNIKTDFEFINSTLFSQLGKLYKEGDRASTPLDG
TTAITVKTLENAYVTDLDGFDLKQLQNYKALKECIGNMYMGLVLQRNSPFKMKFDE
IAGRIMQSGILTKWLMDRLYSQKKVQDSFFNSYVSNMKFSIITADRLSGGFTLLVTGY
VVSIIVFVLEMFYTRYTYPFTEX
>IdupIR52
MAGCAQFIRDLWARNVSFTINQNQVCRTFLVPKPQMASETYFLYRPYQYGVWGCLI
GCLILLTFMSKVLFWLIGFKIDYSTVVISLVKIYSAGGITSFDLTWQSCRIFFLSVLLHS
FLLVTYYSARLSSSLAVIRYSRQINTLQDVADARLPILEFTDLKEDLTVINSSLFHQLA
QLYTNNVEAVDNPALAVRTFENSYVTGLNQISDSRQLTGYKALKECLFNNYIGFPLQ
KNSPFKNRFDAVAKRILEGGIMTKWVRDVIWSNRKEQEKFFSTEILGVGYARITSARF
LGALLTLMLGYLVASLVFVFEFLVGRIHX
>IdupIR53

VNVHISNLAAQTGVSRFTQINSTWITTELNYVTDLLVVLASNYTLWTHKYVGSQGSK
PYMVDQWFSNNHSFLRNQDLFPDKLTNQMGRPLRVAMFSYEPYVIFEKEGLNAYK
YLGSEMKLMATLADYLNTTLVPVINEHDFWGAVWSNWSGTGMMGNVVQDHADIA
GGALYRWETPAKYLDYSKITVRSGITCLVPAPKLVDAWLTPVHVYSSQTWIAIMIAF
LLYLATLFVMGLLQEKVTKKTLTKFHKRKILGLSLTSIAKPFIMQPVSTKEINQYLSG
KYLMGMVFFTATVLSTSYDSGLATIMTIPRFEAPVNTIHDFLAKDLMWGGISDGWIM
SIENSTEPTLRALVSKFKAIPSDNKLRELTKQGDHAFAIERLLNGNYAIGPYIKVDIIQN
YHLMTTDFYFELCVLMLRKSSVLLDQVDMFVLRTFEAGLILFWQNEAATQYMDQN
VQKAIRYRQKVHEIVQLKLFHVQGAFGILIFGYLIASLVFLFELFYKKTVX
>IdupIR54
MSHMDVLIKIILSQYFTKSSCIAVVQDGLNGLNIETTTPLLWFTMNHTRNLFNHIGCH
AIVVQVQDPVETFQKLELEIKLHPDRFNKRRYLFIHDSQTDNWINIFRTTELNYVTDL
LVVLASNYTLWTHKYVGSQGSKPYMVDQWFSNNHSFLRNQDLFPDKLTNQMGRPL
RVAMFSYEPYVIFEKEGLNAYKYLGSEMKLMATLADYLNTTLVPVINEHDFWGAV
WSNWSGTGMMGNVVQDHADIAGGIIFVMGKFRKKRX
>IdupIR55
MRATLFRLVMLYGVLEFSLVGAKQKVKIDVAIFLDAGEHAQATRSVITNALNVIKA
KTKYQVIGHMPSLEKENTFEENTFVAGQIVCDKALKGLAAIFGPESSDINEIIQSTSTS
LEIPQFQTFWNPNWQQSPTTFNLHPSAGSLSQALATLVRDNDWKRYTVIYENEDGLL
RLRESLKQRKPNDLTLTFRKLGPGPDYRSVLKQIKNSEDFRFVLDCKADHILEVLRQ
AREVKLLEDYHSYILTDLDAHSLNWNEFNDVSSNISTIRLINPDSDTARYLGKLWKIN
PNQLKTQTALMYDALNVFMTGFRDLAQNEEPRVTPLSCSGMDVSEHGATLTKIIKKP
SKHSRPILPGFLSGPVNFDATGQRSNFNLQIVEMTKTEPRNFRVTGTWSSASPKEIVY
KLTSEERQKELQKEIQRKNFRVVSRLGAPYLMQKTPEYGKENFGNDRFEGYAMDLM
QEICSILNCSFTFELVPDEKYGNYDPVKKEWNGLIRHLLDRKADLAVCDLTITYERRI
AVDFTMPFMTLGISILYAKAVKEPPELLSFAHPLSLDVWLYMATSYLVISMIIFLVAR
LNPNDWENPHPCDPNPEELENIWNIRNCCWLTLGSIMAQGCDLLPKX
>IdupIR56
MFLTLLYITLTFHRAENIIHSLKPDVSNIFPQTYLNKLLLSSKINQTGILVINSNLQFKVP
TMYFNTNQVLPNLGSLYQCKRLYVFLDATGGQPIPKSLNKFAEYVGLLEKSYIVVLL
SKQPDRTVLSFFIQNKIFNVAFVKLNDMNAEFFYMNGNHFERSKRFRWPGIPRNLTV
CYVDVVPYTSLGPNEKGLEFLIVDEAARSVNLPVTFARLTRSRITETNLGVFEQIRNR
NCDVMQGEVPPASVVPLSMSSTYFLTDATVLVAPTFRCARHVGLVMLTSKLLIAFLL
VLTGVCAFGVSQSKLTWKLLVHTLENSLRVVVESDKIKVKLFVATPVLLVTVVVLG
SLQAQLYDTLVRDQQICEVKNLKDIIKRKLVTTVQKGLLENLMEISLPNVDSEALRPF
LIQENNLTKMNSQQCHILREVMFKYLVPRYYLMDNGQSRFKVAQIINRGVYMLLLR
RGYQFVKDINLGLLRVQQSGINVFLEENVCGRIHLKDIDKYSMLLSYYTPVQLEEIG
WLLYVVFGLWMTALLVFILEIIVSKWGNIX
>IdupIR57
MFLLIKVLMVFEPIVLSICQQQFKIVNILQRDQLSNVQYFEDVLLGLRKNDVTFTRVM
IAESDEEQKKICASLSSGFGLILDFTWSGNEAAQELVNNMSLPYLHIDVSIAPFLVLLD
SYLDSRNSTDVLVIFDKEDYIDQALYHWLDSTRLRMVISDVIDRTVAQRIKQIRPIPTS
LAIIASSSNMNKRLHEVLSEGLLKLPDRWNLVFTDFNLDHFDRTYLENLSVSLLYLN
KLLCHDFLQQASCPSNFDLNSNFLHWLAMSLNRIIDTIFDDYLEFPNEFQCFKAVFPE
NTKQRFDEILEMTVADNNNVIELQQGKTLRVNVKGSVEKMVNETFQVIAQYKNGRL
RLEQNKQFEPIKAFYRIGITHALPWSYKEQDKKTGEYYWTGYCVDFAKKLSQIMNF
DYEFVEPVFGTFGEKINGTWNGVVGDLAIGDTDMAITAIIMTADKEEVVDFVAPYYE
QSGITIVMRKPVRKTSLFKFMTVLKLEVWLSIVAALIVTGFMIWFLDKYSPYSARNN
KKAYPYPCREFTLKESFWFALTSFTPQGGGEAPKALSGRTLVAAYWLFVVLMLATF
TANLAAFLTVERMQAPVQSLEQLARQSRINYTVVKESETHKYFINMKFAEDTLYRM

WKELTLNASTDDTRYRVWDYPIREQYGHILLAINDSNPVENASEGFRITNEHLDADF
AFIHDSSEIRYEISKNCNLTEVGEVFAEKPYAVAVQQGSHLQDDLSKVILDLQKERFF
EELQSKYWNHSAQGDCPSTDENEGITLESLGGVFIATLFGLGLAMITLAGEVLYYRR
KGQVEKEKLKLHKAGNIPATIRGLMKPPGKIGVAPREVGKRSVTIGSSFRPANLRGN
LTKELETLNVSQVTLYPKARNRITRVDX
>IdupIR58
MKPSKLFELTLFWIGTGYCQIVPLSTIPEHYEQLSRILKTQLTNFNGNIGNNVGIFIADN
VDEDAILIISDLLKIKHSRGIQIMTILPTNETTNMPAFTDTIILMDASNIQDVDDILNPML
NQTFIWPRDGHYIMVLTNIVNTKTAKQLIKRLWIFSRLINYALVCMHESSPQILTYNP
FNNQII
>IdupIR59
MFVVNILCFTLLFQFGSANQKIKLGFITTDNEDLNVNWNVLTDTLDSQLEADIQTLQL
KEKEFLAATNTICNTANQEDGLAACVDAGSKDLSNNIRAVADHLGVPYINTDWTPR
DSKQFDTTFSVYPEAALLSQAYRAIVESLDWAKFAVVYENEESLIRMQDVLQLQEY
KSGMDNNTMLIKKLDENGDSRHILKEIRNTKVYCIVLDCSVDKIMDILLQAQDVNLL
EFLSTHIFLASLDAHTLDYSPLNTKTNITTVRMFDPSLPQVQNLVAKSFPEIRADQLTV
KMALMHDAMLLLAESLNAIKSTHADGEIVYEPLFCNASEKYNDKYGLVSTLQEVTV
TGITGEINLASGKRDSFELNMVELSKSRTKLDELIGSWSAQDPDQVIHKRNATETEAA
IKERIRNHQFIVTSKTGDPYLMNNPDPQAEGNDRYFGYSMDLIKEIAKDLNITFKFMI
TKDNAYSNLQEDLTARRADLAICDFTITPQRQADIDFSMPFMTLGIGILHKKTEMEEE
ANIYGFMGPLSTVVWMYTGALFLFISLILVLIARLSNEDWENPHPCDENPAELENIWN
LKNSLWLTLGSITTQGCDILPKGACSRIATASWWFFSLILTSSYTANLAAFLTMSKKD
DSIKTVEELAAQSKIKYGLVKGGATASFFEYSNNSLYQRMWNTMINEKPSVFEATND
KGVERVESTKNGLYAFFMESTSIDYQMERKCDLRRIGDLLDSKSYGIGMPLNAEYRH
SINSAILKLQETGKLAELKQKWWKEEREGEPCPSGPPESSDALALKNVGGVFIVLAA
GIGIAFLIAILEFLWNVYNVAVEEHISYLDALKVELKFACNIFVTKKRAKPLLSESSSI
SSDSKSNKGILIGAGSVLNINTSILNRIGASMEPNARDSQTKKSSKASSX
>IdupIR60
MGDDLTSGLADIGGLSLFLYEKLLSVVGYPHGFMNIVVAMDKAHSPTITNLFNIFDL
YTWILTLAFSIIYRSLILLRTRIFKLVKEKSDVSLRIIILAGTILNIILSQTFQSWITTFLIYP
NPTHVIETVDELIESKLPVYTYATWETLVTERLNDQY
>IdupIR61
MNFFYFLLGHDFVTFNLDESIAERYRVIKEAIHSEFIAFVVRRNFVLLPKINQIVHRLR
QSGIIDLWTRQATSSRFVIYKKPQQAKNLSSRPVILTFEHLLGAFYILLIGQGISSLVFV
LELFNQEKX
>IdupIR62
MGLQKAISVVIKMTVQQPVSRKDVAKYFTKSWMMLMLAILAIMVVNIAYSGGYAS
VLTKPLYQNSIDTLEDFLNSDLPFGSRQTADWIETKFHKNIHSIFETFPCLHISFDATKV
RPVSRIVTVX
>IdupIR63
QKYNLLISIHIKSQASHTSSLLIGARNCISFFVVGLAIMLLVLVTVASLTITISCTEYTID
SNLSKRALQKSHEKPRIQKLSNLFLEPDFYSEYPSDSSLINLLSKIAKDYLYDCTTAIL
YDNYTNTQDHVFLRKFFQQYPLTHVHAAIPENYHIPIGELAFQLDNKCVHFIIFIKDV
MLCQDIISKRSEKVVVVAKSSQWRVQEYLTSEFSREIANLLIIVKSEKYMSQKQEAPY
ILYTHKLFVDALGSSQPLIVGSWSEGNFSRNVTLFDSKLKHGFSGHRFIIATAHQPPYV
IKTRRNELDEFEYSGIEVRLVDLLSRMFNFSTDFKDSSEIKTMGSGDAVIKSLKGGNV
NLGIGGIYISHSRLNAGTFFWHSEDCASFISLASTALPRYRAIMGPFHWTVWLALIAV
YLGSIPVFTYSDKLTLKHLLKDPLECENMFWYVFGTFTNSFTFSSKNSWTRAEKGVT
KFLVGVYWLFTIIITACYTGSIIAFVTLPVYPAVIDTTDQLLSNRFQIGMLNKNGWPD
WFQNVSDKTSQKLLRKVDYVPNVESGLVNVTKAFFWPYALLGSREELSYIVKTNFSI

GNKKSLLHISQQCFVPFKVGIILPSHSVYSEVIAEGIQNILQSGFHIKIKNDIEWETMRS
ATGKLLAANSRGGGLKMLPPEDRALTLDDTQGMFLLLVIGYVVGGGILLSEIFGGCF
NLCKKSKSSRASSTSSIPSNPRFHERQTNRERHQSIHLGSSDRRQSFHSMQSKEFALAK
IASKSQSFQLEFEEAQNLDKAEEESQVDYSDTINELFDNALSLHNDTVSELEIHSSNSF
KDSKX
>IdupIR64
KISVVKTNIKLFVTVPLLLLTVVLFGYLQAQLYDLLVTDKHKCGVKNLVDIVEKKLV
TGIEPPLIQVFELLGVPHVNLNTLKQYLTPDNVVNLRIKLDSDKSHVIRTILFKYLVPR
YYLKENGDMKFRIVQTIHRRSYLLHVRKGYPYMQDLNLGIIKMQESGINKYLEKQV
TGYMDLKYIIKRSTLLSYFTPVKIEEFYWLFIFVLIMWVISVVVLLLEIAVHNWYNLFL
CYYIGKFX
>IdupIR65
MWGLLCGHLVDFKAPKSWPNKFLINVWGGFSVIFVASYTANIAALIAGLFFQNTNSN
YNDRSLLSQKVGAPRSTAAEYYIQKGNPILWQHMNKYSLRDADEGVQRLKNGSLDI
LIADIPILDYYRATDHGCKLKKFGDTITEDTYAIGMTKGFPLKVGX
>IdupIR66
MMWSVLFNVLWPLLTLANQTFHFDLIKSLTDQNRIISFICNKTASFTEYKRLSTSGYK
IALVSEPDFSAVSPHTDQNEHLLFMVDFNCHKSRQFLQVAEANKWFASPFKWIIDYG
TKELNSTEFLNYSILVDSDFNLLLYVDMNLEISVVKLFKTHRKSESVEIENYAQWTRE
YGLVYDSSSRLRRRKILNVTLNACLVITNNDSLNHLTDKRYILKSTILFMESIYNFRDK
HIDSIAKVNYVLVKTVATIYNINLNFSVVDTWGYKDNDSQWSGMMGELTRNEADIG
GIKEKSANKLCSMYSX
>IdupIR67
MVDFNCHKSRQFLQVAEANKWFASPFKWIIDYGTKELNSTEFLNYSILVDSDFNLLL
YVDMNLEISVVKLFKTHRKSESVEIENYAQWTREYGLVYDSSSRLRRRKILNVTLNA
CLVITNNDSLNHLTDKRDKHIDSIAKVNYVLVKTVATIYNINLNFSVVDTWGYKDND
SQWSGMMGELTRNEADIGGTSLFLLKERVDLIEYIAMISPTRSKFVFRQPKLSYVTNV
YTLPFDSKVWYSTIVLTILMALALYGLMKWEHAKNHFLSENASHSNQAELNDSIKD
VVFVTIGAMCQQSASALPYSVPGRVATLILFITLMFMYVCYSANIVALLQTSSNSIKT
LEDLLNSRIPLGVDDTVFNHFFFTVSVLTX
>IdupIR68
MYGFMRPFSWTVWSYIITLYLMVSFAILLIARLDPDDWENPHPCNSQPEELENIWGIK
NCLWLTLGSIMTQGCDILPKGVCSRIATAMWWFFSLIMTSTYTANLAAFLTMSKKDE
SIKSVEDLANQNKIKYGVMSNGSTQTFFENSNNSLYQKMWATMKNENPGVFEENN
DRGVERVLSTKKGLYAFFMESPQIEYELERHCGLKKIGQYLDSKSYGIGMPLGAEYR
HQINSAVLRLQENGKLNELKEKWWKHEDLEEKQCGPKGVEGKKDELTLANVGGAF
IVLAAGIAIAFAFAFIEFLWNVKTISVEEHMTYFEALKCEIKFACNIKVTKKAVKHVIS
ESSSEKSEDGRSMARTVLAGAGSILNINASVLNRLAYQEHX
>IdupIR69
AINSSKPLVRFTVKNFKENIGKLHFKPSMYIFQFLDPNTLNYTLDVLVNSEAFTQSQF
LVLCHPNTLQDLVDVLWMFRLPNTVLITVSEQFKLYTIQLYKCGAKSTPKYITTLNN
SNTICSAGLKYHLRFNFKESFMGCPLKVLWIRYPPFVNQANESQKGIFIDFLDSLAHIT
KRELNMNSDDSEYFEDVAEHFIYDSVVEDLKEADLFLSSLIGTTSIFYLGPVVHIDNIL
FIVPKTPLNYWKSFSDVLWKIVVVVTVTVIAVSSVIYYLGKDYRSLTNVLKLVLFFY
GTLLGNGYRTQLPRFSLLRLYFSHLLLLFLVISMFVQGNLVSVLSGPLFEPPITNIQQL
VDSQMPFKTTELFKTFFQIHPGFITDRAIIQLVDRLQITNEPILLNTLDDVIKRRNYATL
SVNALVMLRPNLEKTFSIFPLTQMFTCFAMDRTSFKSPQFYDWILRGVETGFMEKYK
KLYHFYLALKWNVEAERKIFVLSMDQVQPVFRTLLILHCVSILTFLLETVYYYMKSV
IGVMYGKTTVQSRDX

Ips duplicatus Gustatory Receptors

>IdupGR1
ITSGVGLLLICFILSHVNFFPILINKLNSIVFSMNQIAFHYLIKQPVVLLQVFGFFPVKGL
TNPDPDYLSFKWWSWRVFVNLISLAGSMFHLICFVLDMKFVNTVELNALEFHLEGV
LLNLIFLYMAKTWAAFVREWSLVEKSMKYYEGPKNAKIRIFVAMVSILGIAFLEHTV
VTSQIALTSIRNNPGSFFNASREYFVHHEFVEVFHYVPYSIWVGLFFKLLIIQKTFIWSF
IDVFISTVSICFYCKMVQISKKVARLSAYEEKNFMIWRTTREDYTKLSKLCQIVNTKL
RWLIILSFFNNVYHILSQLFNSLRPNDDVVYKIYFCISFTLLILRLTTVCVYAGSICDEQ
DRLITVLTTAPSGVYNIEVERFIMHLDTYEMALSGSNFFKITKGLLLKITSAIVTYELV
LIQFNQKEFQNALLX
>IdupGR2
MLDKSVFPSRSDLANVEVWYNVAKFFAVTPINNNYYLSLLYPSFLFLIFLGSTMFSFY
ERTFFIYKHFNISQIILDTLQGITEFMFIEHVVVVSVVKKPLWEKLIKRIFQMEQQFNST
LLSDIRDSSKKNFYVCLIVCHFVYFTSHIFDTIANWESIYYSLAFIIFRFTTYYIMFSTLFI
TFVCTWIKNRYLYLKRLLKSSSPPRIKINLLQNEEIMQKLSEFSHTYKSLYLVVQEVN
YIFGTYFIFIPICTVLEILNAVNYGMPTKKGTLQNAVLADNMVYLVLYAMCNIQIVMS
CQRVLDANNQVWKTCLILHQQMKTPELREEYLTLAYFVQGLSPEFSATGFWVVNQT
NLSTLFSSVMTYLIIIIQFNMTLDKVFENDVFGNDTTTEX
>IdupGR3
FQDIDNYCVGYIVKHYRVLWLELSEILQKLGNAYTRTYSTYCLLMMTNIIISVYGFTS
EVVDHGVKFTFKEMGLLVDAIYCLVLLYIFCDCSHKASENIAERVQWTLMEINLRQV
DEATVREVQFFLRAIQLNPPKVSLRGYTVVSRGLITSIIGTITIYLIVLLQFKISLVNMK
NX
>IdupGR4
MYKNARVTYLTNTSVSNMAFYMDLGSSRKKFLWAIQIVLSFFHFFGLIFQIVGKLSFL
YDSLNFTDTTAQVMDLLRDGITTVTNIVGMYCLINFIVNLNKFATLHNRTGYLPNKH
VFTTIVVVLILSTISISIMHVYYISTHFKGMVWYILPSKIEYVFSNIVLFSMLYFARAVK
DHLTDVNEYIKGQIWPYFDRPLSFSVNSKLNMDKCLYTLREITGHHEKVCDLLDSVN
DKFLPLLVLIIVSVTLNALYDITMMLEFTLRSTTLNDESYALMFFGSMFLVESGQGAL
AAWIGEQIKNEAHETTKICYSLINRVPFSIPVSYQAQIEKQLSILLDQSKTRKVCLHAG
GFFQLNWGILGSVTSTVATYTIVLIQFLFKGTIX
>IdupGR5
GILVMLAQYYLQPDMLLWHTFGYYHILAMLNCLCSLWYINCTAKGRAAGWIAENL
SLALESKEDAAKKLNDYRELWVDLSHMMQQLGTAYSGMYALYCLLVLLTTIVATY
GCLTEILDHGLSFKEAGLFLIAFYCVTLLCIICNEAHAASRKMGPEFRERLLNVNLSSV
DVRTRQEVHMFLTAIEKNPPVMNLNGYANINRKLISSTVTSAATYLVMLMQFRLTL
MRNAAIANRKGNVTMIGHQNASFGFX
>IdupGR6
VQVFNWSWTIIWNYVDVFIISLCICFTYRLKQIESKIDFLVKLRVRDVTYWKRTRENY
NKLSELCSELEKSVSPLVLVSFADKFYTVLYQLLGILESVFKLFLTIHNKPITGNITVD
QNMDRKKTLEX
>IdupGR7
MTIIRKNWVQRTLTATCLIIYLIIITYVGLPPIWLSTLAIQVKCFHEIITIAYNYKKLPEV
PLFRTLNWYFLFVANYYFGGETFAQYLEAFIAKYLVVNLLFRYHRFLSFCLYMGGII
WFLNLLRRPVIRQQFSLLAWVHFLCIIVVLQSYMIIQNLFEGLIWVVLPVSLVFVNDIF
AYIFGRVYGKTPLIAVSPKKTMEGFLGGGFGALTLGTLIAWALCHIDHMVCPTRFILI
EDTIKMTTECNRSYIFQPISYNLGLFSINYYPFLVHVFVLTMFASLIAPFGGFCASGFK
RAFKMKDFGDTIPGHGGMMDRFDCQYLMATFVNVYIISFVRNYSVERMLARAMYL
DESDQVEFYHLLKNSLRSQGLLETTX

>IdupGR8
KWAVFMGIVCFWICANMSQSFLIAFYGEQITKEGEKTTRICFYLLNKVSFQLKNDDE
KLVKDELNSFAFQSYTHIPSLSAGGFFDVNFKVLGLMISSVTSYLMVIIQFLLRPQNN
X
>IdupGR9
LCALTVYLCNLTVMEQLRNIKVSFEKLQPNRLPSVSSLKCTKRLYLNAVEMTWLMN
EYFGPKLLIHFANSSIRLLWVLTSSIQVWNPEQFTNLLDGDWHQFYKAVIIAIFGLIPA
FIITMSCNKILKTVEDLISKCYELQYKYPHGSYEQRELQAFWIYATKNRLSLTALQFFE
IKPSVLLGLVGSITTYFIALIQFKAX
>IdupGR10
MLNPQNHQHNMTITKALKKLVLLAQCFGFFPVLGISLPEQRFTYKSFRMLFSLFSLVG
SVFLCVMQLNKAVRTKMGIDQIDYIAKYVSSAYVSVVFIELAKEWPKLMQSWRNVE
TDMKSYGFPRSLNRRITILIAFFMVTFSVEYILHQASRVARALNCHNDTAKSMKFFFG
NLTLSHVFDYIPYNIPTSLVFQYWFCQMEFAFTYTDIFVMVISMCLASRIRQVNNRIK
LASRKQVMNEAVWKSLRKDYTRLETLVEVVNDKISNIILVSFLPNIFTILTQVFSTLKP
KHNLLETVYFYVSLVFLLSRTVMVCICGAMVNEESRKPLHILNSVHHSVYNEEIDIFV
NQLLNFEMTMSGKHFFKIKRYIILELAGAIVTYELVLIQFNRESLNTQSKSTRCTX
>IdupGR11
MYNNQGTPYPPSMMIGVMDNQNIFLDGAGAYYNGRTVHPITKVAPAPPPRYSNGDL
MEAFDDSGELNSRIFSCLKPAYATLRLFGLMPVTQSGPVFHVTAKWIIYSFMLLCSLA
GFLGYLKYFNITITRSAEGRFEEAVIDYLFTVYLLPVALNLIAMYEAPKQAEVLTQIV
AFERIYTRTLRTRPTLDMGSKQLILISVLLILGCVVMVITHFTMANFIVYQVVPYCYV
NIVTYIIGGSWYIYCDVIGKVATSIAEEFQFALKNAEHSSRVADYRSLWMMLSKIIRN
VGNSFGYQLTFLCLYLFFVITLTVYGLLSQIQEGMGIKDIGLTITGVSAVTMLYFICDE
AHYASACVRTYFQKKILLVELSLLNEEAQQEINMFLRATEMNPTDMCLCGFFDVNR
NLFKSLLATMVTYLVVLLQFQISIPAGNDILTNSTLNSTKTSX
>IdupGR12
MSVQKSNFVFLGPLLVFTYVFCSFPQPSVKNSHETQRKVLIFFNRIFVFSCAVIWLILD
GVAIFSQSYKLSEALMTTPQSILLPAEIMGLILQGVLAIFSLTIVISFGFVHSKGITNMFT
VLDQIDAVIDFNIKEFRCQFYFNLMIFLFLPTVYEFYSLWFWKEMFQKPTVFSVYFLRI
FQTFQTSLIVTTMNTLCYHLYQRLKRLHSLLLKFDPTSKYFSVLQLKKIRSLLCDQID
NVNTMFGLPLLCFIVTSITIVCYYVICIITSITYADIETFNDRLRLYIVCSIGIFFTTMEVL
TLAWCGALLSTETAKTHKISFNLLNDLNRPVQMGPQLLVWKELKSLAVQSGFRKPF
VSAHGVFHVDFSILGVIIGSVTSYIIVVQQYMTQCDGFHHPSRENVSSELRFLHPX
>IdupGR13
MKLQPGFNPYLNSSRIKIKELRREPESLSFKVKLITFTSKHKKLTATVLHSIVVINLLV
MVHSIHGRYFVYPHVAVFGDDVVKLVNTVSDILLVAVNIFGLYCFTESDFLLETFSFI
DLNHVTFSVNTWKRSKSAKVFSGLFQILPVVPVITGIYLLDEEIDWKLYQYFLPRDIW
YLLMHLMICSSTDFFRKIKERFKVMNDHLERLLSRLVTSNSYQSLSVSEITRIVHSRTQ
FITDIQIITQYHNNLCNYLDKFNSFSKTFTVSSLVCISLNLLYNFTIIIQDVVRPYGNKN
NTKVATLATQPILAITSVGQAAIGAFVGESLEEEGERTSRICYTFLNKLNGNIKTENDI
LVQKELKFLLDQSKSRKICLHAGGFFKLNWGILGSITSTVATYSIVIIQFLLKX
>IdupGR14
MFTVMDTKTHQSDFVRTLLKGFHPKSTFYILMGVLPPYNFQAGHPIKSKYFKIKVFFL
VLSCSVFNYATIYERYVIGRKYQLALMANILSYILNWLASMTFVAFTVIKEKTFYNVF
GELGTIERHSNSPDSTLREKALARKINCQLGVFHIMFLQHMIVYHAWYFRWIHRSVT
VSFNFFRMDLLYTYYTNIILCATELLLESCKYKYQELNHMIFTVKHTKIYDEAILSKQI
SEIRFVFKKLSKIVVMLNDILGSLFIIMPLMCTTLLLELCIYVKHVWPLIPDSSYLTTKV
TKVLIISVGTSKILILSDQIVTESKKIMTTCYNIQDCISIYSKPYLELQEFTNKIPDFKVA

LTACGFYNIDRSIMFSIIGSVATYFIVAEQFWDYRVNKEFNITELFQNRSKNETDLETF
SISHYX
>IdupGR15
QIVFSKLLQSRMPNHPESCPHRLTSGQCVLVAWVGEQIAEEGERTTKICYQLLNSLPT
LPTEHSQLMEKQLNLLLDQSRSGRVRLHAGGFFKMDFGILGSIAATVATYSIVIIQILL
KGQIX
>IdupGR16
MYLAWRWQKILKEYTYVEVSMRNYNTSKNVKRLNILTSTFFMFFGLIEHILFIFTNLY
HGMTCIKYEEHPVEMYFKCAFPQWFELVNYSHWAGAVVEVTNFISTFTWNFTDLFII
LISISLREKFNLISNRIKHCKNPPNKFWKEIREDYYKVSNLTKVVDIQIAGLVLISFLNN
ILFLCIQLYNSIKX
>IdupGR17
MCALQSEAPKSDCFPLEIRFLNTIKRRKLIFRCLQICMVGLHVTLFIISQKGKCLIYDSL
EPFNNGVVKFVNCLSDFMLLSFNLLVWTVMPTTEVFVELVNFAWKNNLLMGRDCS
ALLTVVWVLRLSVFVPILINIYFLACLIGWDMYQYYLLRDVEYWGHNLVIVIYTFLA
LRLVKCFSNLNNVYLREFSDQYLMYNRVSIIQKSDTNKLINVNGLLTTNEYLIKLIKL
RQTFNNLCDIVDKINESTKQTLNVMIMCIIGNIMYDCTTLIEFGIKPRIIKGFEITPYIFAI
HLFFGGISVAQAALVGYVGEALKDEGQNTTKYCYSLQSKLPAYPQSETHKLIEKQLN
QLLDESKSRQVCLHAGACDITWGILGTIASTVSTYCIVIIQFLVKX
>IdupGR18
MIFIMIIMKQLLSACGIITINVICMRCSNSVTDCLFSTMEHYFNYEILTQVSTSLWGLLL
LAHQTFIIINFSLTNIVLKGRFNKVPDNKKIPYYETAPETTLKIITKLHFKAVKAANMS
SCTYRFQILIILVFTLLRIVENLHYSLNNRVSSLMTIGAELFWLIMIMANLFITFFIATKV
EQIAKKSSNIINMVLCYHNPALQRERDMFLLQSFHQAVFLQAGKFCPLNNNTFLTLW
GAALTYIILMQGITKX
>IdupGR19
MSRNSKVQPTENKTQTMFKERQNINTQYHHVKKVFRLLEKKRVVYPLTVFLLVLVL
FGHIYSLLGKPKPFLNQHVAVKTCEHVADFCLTASIVIIIVYLPKNGKNFFQFFTSLYP
EGNQLFGPLLAMKKHHFWRNLLICNVCIGALITLDASFFLIHFGWDTYKYLIFGDLQF
YVVYNLALLLLLALARKLELRFAELNELLEYKTENFLKSNNKKPENEIETHLSFILNSE
HFYSVRTFRVMHYDLCKLVKRFNEMFGRIILSTVVFSIANILAKVTFIIDFYVAPSVDE
EKNKWAVFMGIVCFWICANMVCIX
>IdupGR20
MKAILFKKIVIIKNHLETLFQDDKPPELLVMILATSYKKTARLVQLVLVLSLTYCYIYC
AYGKLLINSQMPQMHNNLLAFFTVVKDFLFVTANLIGIIEITQSSSLIRVFRSILRIKTL
QKKGSVTFKVYLTMLFVLPLISTISGFYFFGTYFKWSVLKLYVPKDLCYLGVNLTVIS
IASLVNKTRERFTAFNNQLEVFCKQYDHSLKTSKSEDVCRLKNLQNYYDTLCKLIKQ
LNQHTSLILIVIGTCIIVDVLYNVTALIEFGIKPKIIDGIDLNAVSILQQILFLAATIVKNK
PKILNVX
>IdupGR21
MDFFFSVMHYWACISTLLIFEWLKFLTRRYAHINQSLQKAFSSPVLLTHYVVKELQII
GLVFTNLEEITSSLNELFSFHLLFLLVSSIFTTLYGLSFAIILFYSLQSSLAAIVYCTIFFA
NILVIVLAYEQLQKTSKNNIRTCLMLQEMLTPGHLRNELEILVIIMKKLQPKFSVIGFF
QLDLQPISAHTSIVFTYLIIVLQFSLTFAX
>IdupGR22
MILYMTNVYISVLALFKHQHFTSLFETVLDDVIASHNFLVHFLCFITTQLINTVLILYA
QMKRSNDLKYQLTTLFTDGFLTCRLMVAFFIMFTVVNNIKTRAMKLNSKLAHPHSW
IYVRQLRVEHNSLCDHIQSVNKLFGVTLLMVIFYFITTFLRHAVFISKNSDDNFVAIVR
CLWILTYGFHAGYLAQIGERIRNEAQETVSISYKQLLQNSHSKSQEVLRNYYMLGQQ
AFNRSSTITAAGFFNVDNSMLLFMATTFFTYFIVVMQLLSGRSKSFEX

>IdupGR23
MQLAKCWPKLLKTWCTLDKIMNIKYGYPKGMDLQIKLLMSVFVTLSLVDYLLGIGN
NYEQVAAAYGRNITAELVFASSFPQLFAYVPYSIFTTVFCTIITVHANLTWALNDVFIII
ASTAIAMRFRQITEKLQQEHSQKTMSILFWKGIREDYDRLASFCKELDSHISVLVLLS
YFLNIFFLLIQLYHGLESFTSTVRKVYFVSSFVYLIFKTTTVSLYAAWINDESKAPTNV
LNSLDSSLYNVEIRRLLTQISFDKTALTGCKMFVVKRGIILSVASAIVTYELVLIQFTQ
ANLYGKGQX
>IdupGR24
TMVIRLLYQQSNVANQVFRFYIFSGVLLTCFGCLSSISFLLAAGKTVSDSKFGLFNIVF
ACASRLVFLAIITLALDGVTVAAQQVSKMAVKCMAHVRNGAVVEELSYSAIYAEQL
VPIFTAGCFTVDRHLLSSCLTAISSYLIVITQFSTLPPDYTNVNQTKHFX
>IdupGR25
LVVTMNLGIPKFVRYAVIIGLLFQHFYASYCLYITIYIPTNVRPTIYIIDTTARTLLITSIA
ISIAVFDGTLNKFKKPLQNLTEDDELKRKFRYLRLLYGFLVSTVLVHNCFMKVNIYG
WGYYRYDWMQDVQLIIYTFILYCFIKVLDDVSNKFSVLNYHLNDIGNVIMTKSRVV
NDNAWDVNNMIFRKQIVTSTQCIAMRYNMLCKSVEQINDRFGYIMIVSVMYLIVSKL
FYTTFTVHYVINKVSMEDKLSNFFQALMIFILSLVMFVVNTIYLASVGNRIDVEICKS
KKACFNNINKYLEFSKVDDNQIPTAFLQLHDQIQSKHVSVSAAGCFEINLNLIGMECS
VLITLGIYALEFLLYYSX

Ips duplicatus OBPs

>IdupOBP1
MNWLSIVCLQGLLLASFNDLTHANYVPNVIYKISNFCVDDSVISTEIAEHLLQTPEKD
LIEQESCYLHCIYVEMGLLSENGDFVHSMFQKLNEEGELPKLYLECLATIQNVEKCN
EIPKLSECNLX
>IdupOBP10
MKLFILLVILVGGMGCVFGAMTESQMKAAFKLIRNVCQPKNKATDAQIEAMHKGN
WNQNKNGMCYMNCVLNYYKLQLPDNSFDWETGLKVVEAQAPPSIAVVAIEAINNC
KESVKTLDDKCMAALEISKCLYDTNPEKYFLPX
>IdupOBP11
PHTATLPPPPHKPVEPEPAPHADISAIKEHKPPVTHEGRCAIFCVNKKLGVMSEDGSIN
DFYTEWIDKVKVDDPDTYANMEKVYYTCREIEKKNDFCDTVIEFVHCMKIEVEKY
>IdupOBP12
MVYGSNISKSLRALVVNQVLIVIMLGKVLISTLFLLIPNVMGLSDEMQDLANQLHGT
CIEETGAAESAIDDARNGIFREAESFKCYIKCLLAQMAIIDDDGVIDVDAMVAVLPEE
YQERSEPIIRKCGTVKGANACDSAWLTHKCYYKESPKDYFLIX
>IdupOBP13
ICSKMLFLMLILVALSESLHDPEEEFLAMQHDCMIESGATDVMMSRAFHGHFPDVPE
FKEQLVCMGRKGGVISPDGTYHIEILKQKILPFIEDEPLLNFILEDCY
>IdupOBP14
EMEKQKEIQTGCLKETGVDPSFIEKAFMGEFSEDEKFKVQGLCFHKKLGAIDEEGKIN
EDKVVELLLKKFPDEELVRISVKGCIDKKDTPQDTVIEFTKCMYX
>IdupOBP15
MRNLVWLACVLVGANGLRQALIDEKKEMIMEHLLECIESEEATDADLAAMKSHKPP
VTHQGRCAIFFVNKKLGVMNEDGSINEPHTEWIDKVKADDPDTYAN
>IdupOBP16
MNFVLLVIALGYTGVCEGIQCGMSKLNSEQFKSVIAECIKDNETLTRIFDLSSAVSTEE
DDETSMSSDEDLPAPLPTRVSANSSAVTNNQSNNRNVKLVRNGRSTSNKIIKNSNQD
NQPPRFGTGISTSTDRSTSMEDQEEANVTGNNVADDPDDEMCTLKCIFEKLELTDSS

GSPDHKKISSALTSSATGREVKDFMQETTDECFQESEQSNTTNMCELSSKLFMCLAE
KGRSNCEDWPAGNLPFX
>IdupOBP17
MTESQMKAAFKLIRNVCQPKNKATDAQIEAMHKGNWNQNKNGMCYMNCVLKYY
KLKLPDNSFDWETGLKVVEAQAPPSIAVAAIEAINNCKESVKTLDNKCMAALEISKC
LYDTNPEKYFLPX
>IdupOBP18
MGGYPKSTTYLVLALVLVNLVSPNRGAESTTRTPISDDALEKTLSDKRYLTRQLKCA
LGEAPCDPVGRRLKSRFVTSHRINSCSSISLSPFPRKRVCIIALPYIQHTX
>IdupOBP19
NISKSLRALVVNQVLIDIMLGKVLNSTLFLLIPNVMGLSDEMQDLANQLQGTCIEETG
ASESAIDDELNGIFSEAESFICYIKCLLAQMAIIDDDGVIDVDAMVAVLPEEYQERSEPI
IRKCGTVKGANYX
>IdupOBP2
MKILILLACVLVGINGLSQVLIDEKKGMIMEYVMNCFESEKVIDADISAMKEHKLLV
THEGRCVIFCVNKKLGVMSEDGFINDPHTEWIDKVKVDDPDTY
>IdupOBP20
MAAGTVFLYFLVVLLLVDQSVSRMTEKQLAAAVKLVRNMCLSKEKAKLEEVDKM
HAGNWDVDHKTQCYMWCALSQYKLIGKDNHFDRESANIQVDTLLPESMHDYVVG
CMDKCENAATNFDDKCVAAYEYAKCMYFCNPKVRFIQKYFYLX
>IdupOBP21
HVEDVPDDELCTVLCMFVNFELTDSRGSPDHIKISSSLTCSATGREVKDFMQETNDE
CFQEIEQSNTKNMCELSSKMFMCLAEKGRSNCVDWPAGNLPFX
>IdupOBP22
MKTVLALVFLSAILTVKAEVTKEEMEKLKEIQTTCLKETGVDPSLLEKAFKGEFSEDE
KFKEQGLCFHKKLGAIDEEGNINEDKVVELLLKKVPDEELVRSSVKGX
>IdupOBP23
MALLTKPLGWYLCLVLLGTSLVHSQLLNGNGYVEKQLLCALDRAPCDNLGKQIKD
ALPEIIGKNCQSCDQKQYANAKRIARFVQNKYPDVWNDLVRKYGKTTNX
>IdupOBP24
DLAAMKAHKPPVTHEGRCAIFCVNKKLGVMNEDGSINEPHTEWIDKVKADDPDTY
ANMEKVYHTCREIEKKNDPCDTATEFVHCMKIEAEKYGLDKIFKDGEX
>IdupOBP25
MKILILLACVLVGTNGLSQALIDEKKGMIMEHVMNCFESEKATDSDISAMKEHKPPV
THEGRCAIFCVNKKLGVMSEDGSINDPHTEWIDKVKADDPDTYANMEKVYHTCRES
KSEYSSRIFLX
>IdupOBP26
MKGVVLFVFAFVSVAYAIDQALINEQMAQLVEYGLECAESEKATPEDIAELQKHKA
PVTHQGRCVIFCVYKKLAVMNEDGSIVEEPTNPMLETLKSKEPELYSNMQSIYKTCL
GKIQSNGDACDTAFELVSCVKHEGEKIGLDKLLSTX
>IdupOBP27
MKVFSVLTCLLYFSHGAPLTDDQKQKVHSYHQDCLKESKVNKALIEQAKKGVYTE
DPAFKAYLFCFSQRVGFQNSVGSIQKEVFQRKVANLVEDPKVLAQLVDQCTEEKLSP
EDTAYHIAKCMRGIVPAIEIFQISPEISYFSNEKAQKHLQVHQECASQDPLGETDIENA
RRTNVLFEDPKYSNYINCFFKKVGLQKEDGSLDREAFFKGFSEVINDNLIAEHLAEKC
LQPQKNEKETAYFVSKCLHENSPVHVELFPREIPVPDELAKRIIENGRACIRDTGVDR
EILDKSRQGIFVDDPKLKAFALCMARKSEIIRENGEIAVSVLREKFSPIVKDPVVVQKL
IDRCALAKENPEETAFQFQQCFYRNSPGHLSLIQFGAVSEAKKQKRVQIIKECAEETG
VPKGVVLRARKGNFEPDPLLEQYFYCINKRSGILNDDGHFNRETLKKGLTEVFNAEE
AERLVETCAKNLDNKLQTSYEGIQCFYKEAPEVAVVFX

>IdupOBP3
MWTVGKLVFVSVLVLIETSALQKSNNKCEIPTVAPKKIEDVINTCQDEIKIAILSEALE
ALNVNEHKASRKRRSTFNEDEKKIAGCLLQCVYRKMNAVNQYGFPTVDGLVSLYTE
GITQKEYVLATLQSVTKCLGKAQKTYPIPAQNGTASTACDVAYGVFDCVSEEVAKY
CGQTPX
>IdupOBP4
TKEEMEKLKENQTGCLKETG*DPSII*KSFKGEFSEYEKFKEHVLCFHKKMCAFDEEG
NIIEDKVVELLLKKVPDEELVRSSVKGCIDKKDTPQDTVFEFTKCMYSLAP
>IdupOBP5
PQFLAAKQDNPKLLAWFKNCQEESGASDDDFELIKMRKIPTTRQCMIILSRMMLTW
KLLTAYGLAVIALLPQFLAAKQDNPKLLAWFKNCQEESGASDDDFELIKMRKIPTTD
PGICMVQCLFSKLRIIENGQFNHKGFVVTFSPAAKGNLKKLAALREIGSVCQKEADSV
ENPQGCNITKIVLDCFGKNKDKMTIX
>IdupOBP6
MKCLILVSVCLFLTVQGEVSKEELEKLKEIHDTCLKESGVDQTMPEKAFKGEFTDDP
KFKEHLLCFHKKIGTIDDSGNLNEERAVEILTKKFPNEKLVRGGIKQCGVKKDTPEDT
IFEFSKCLFGFVPKGIDVKELILX
>IdupOBP7
MKTVLALAFLSVVLAVEAEVTKEEMEKLKEIQTGCLKETGVDPSLIEKAFKGEFSED
EKFKEQGLCFHKKLGAIDEEGNINEDKVVELLLKKVPDEELVRSRVKGCIDKKDTPQ
DTVFEFTKCMYSLAPKDAEADMKELMVX
>IdupOBP8
MLFLMLILVALSESLHDPEEEFLAMQHDCMIETGATDEMMSRAFHGHFPDVPEFKEQ
LVCMGRKGGVISPDGTYHIEILKQKILPFIEDEPLLNFILEDCYHPELTPQETAFRMAK
CVYIRLMEEX
>IdupOBP9
MYSSGVIFLISVTLVLGGKVDLPEDLQELLDDLHKLCLEKSSLQESDHIAFDIKDKNE
KLMCYMKCLMLESKWMKPDGTIDYDFIEIQAHPAVKDVLMAAVNQCRNIENGADL
CEKSYNFNKCIHVADPVNWFLVX

Ips duplicatus CSPs

>IdupCSP1
MKTITSALSLVALLVVYCGARPDQYTNKYDNIDIDEILHSDRLLSNYMKCLLDTGRC
TPEGQELRKVLPDALENGCSKCSEKQKDLGKKVLKFLIEQRRSYYDQLEVKYDPEG
KYRKRYDEEIKKEGLKLX
>IdupCSP2
MKTFLFLLVAVIGVTQAENPKYTTKYDNVDLDEIIKSDRLMKNYVNCLLEKGKCTP
DGTELKNVLPDALHTDCSKCSDTQKKGSRKIMRHLIDNKPEWWTELENKYDKEGA
YKKKYREELAKEGIKLX
>IdupCSP3
MWFTGAKVALSSLVAQKSVLILGTVLATWVPITHGGVTQMTQYTTKYDNVDINDII
HNDRLLKSYVNCLLDKGPCTADGLELKKNMPDAIETNCSKCSDKQREGSEIMMQYL
IDNKPEYWNPLQEKYDPTGSYRKRYLDNKKAEVAVEPVKSDEX
>IdupCSP4
DNIDIDEILHSDRLLRNYMKCLLDTGRCTPEGQELRKVLPDALENGCSKCSEKQKDL
GKKVLKFLIEKRRSYYDQLEVKYEPEGKYRKRYDEEIKKEGLKFX
>IdupCSP5
MGGYPKSTTYLVLALVLVNLVSPNRGAESTTRTPISDDALEKTLSDKRYLTRQLKCA
LGEAPCDPVGRRLKSRFVTSHRINSCSSISLSPFPRKRVCIIALPYIQHTX

>IdupCSP6
MQCLGLFVVLVLGCSLVAAQSPYTTKYDNVDVDKILKNERVLTNYIKCLMEEGPCT
PEGRELRKTLPDALASGCSKCNEKQKDTTEKVIRHLMDKRTKDWDRLSKKYDPQG
VYKQRFEKELSARKLAX
>IdupCSP7
MALLIVSVVILTVGLADAKPAVKHYASKYDHIDVETILNNPRMVKYYSACLLSQGPC
PPEGVEFKRILPEALQTNCGRCTEKQATVTLRAIKRLKKEYPKIWSQLSQMWDPDDV
YVRKFESTFGNRNKIPSVVVNNGPDLGTSTTSNADEPRPDTTTHQIITSPNIMSFTTSK
TSSTPITISSSTSNPSTKTSTTTVGTTTKPPSRPAPIPGLLPINTFFTNPPIPIRPIVNLNLGA
NIGATVSGLVRGLGAIGSRVMETGAEIAQVVFKNITKPLPLX
>IdupCSP8
MALLTKPLGWYLCLVLLGTSLVHSQLLNGNGYVEKQLLCALDRAPCDNLGKQIKD
ALPEIIGKNCQSCDQKQYANAKRIARFVQNKYPDVWNDLVRKYGKTTNX
>IdupCSP9
MKSIFVVIFAVQIAICFAQKYTSRYDNLNLDQILSNKRVLQNYVKCILDEGPCTAEGR
ELKTHIPEAVQTNCAKCTESQKNFVRKGARHLMQTSPQDWQRIARKYDPQGQYVSQ
FQQFLKAX

Ips duplicatus SNMPs

>IdupSNMP1a
MRFPAKLVIASACAFVFIILVGFIMFPKMVKGKIKGMVNLKPGQDIREMFLKVPFPLS
FKVFIFSIQNPEEVLLGGTPLLQEIGPFCYQEWKTKIDVVDIEGDDTISYNSVDTFLPV
WEQGCAPFDTEVTVPHPMILGMVNTVARQKPGALSLVGKAINSIWSNPNSLFVTVK
ANDLLFEGLAIHCGVQDFAGKAICTQLKGEEALVHINDDDLAFSLMGPKNATPGKRI
RAYRGTRDYHHVGKIVTYDGKLRLEVWNNSKCDAIDGTDGTIFPPLLKKEAGLASFS
PDICRSLVATYKNQEKYDGIPVKHFSADLGDPSRNQDEKCFCTTPDTCLKKGLMDLF
KCAGVPLYASAPHFYDCHESYLKGVKGLHPDQEKHAIKILFESLTGSPVYAKKRLQF
NMPLEPNQKIDLFKNITPTVLPLFWIEEGVELNNTYTKPLKSLFMMKTVVGVAKYLIL
LGSIAGFTVGVYLYFKSSDTINVQKVEPQQNGGKSPISTVFNGDSHVNGTTNQGYIH
DKYX
>IdupSNMP_c10
MKDYHMILRGLFGSTYDAVHTTDKQKTSNSLRDRAKKKSQNRKRALTFTLLGIGAL
FSGLFILLVDPYNVLYEWKLKFGPGGEIFSLWEKPPVELYLKVYLWNITNKNEYMSG
KDDKLKFEEVGPYVYREMMTHENVSFNDNGTLTSVPNHPLIWEPSLSEGRKEDDLLI
LPNIALLSIADVTSTKSMFTRLGLNLIIRQTKSQPLVEMTAKEFMFGYKSTLMDLGNT
FMPSWIYFDKLGLIDRMYEFRGDYETFYTGTQHGISNIGLLDKYNGEIKIPQWESPCG
DVTGSSDGTKFPTNLKPEDKPLFFRKSMCRAKYLVHVNDTVQDGFPAYVYNFDPEA
DDNGRVHPENRCFCKERDIDQCKPKGLLDVRSCYYGFPIALSYPHFLDGDKVLFDKV
ESGLNPDPEKHRSYFIIEPQSGTPLKLAVRYQINMSLGNLRNIANTEKFSNMVLPLLW
TEIGMYGLPTNLKIRFKLYLEIIPIAQTCLMYALFIGSALSLFYSIYKFWSSKKTEPISRS
PWIEDDLILNIDRKLSSYIPERKNSLTPKELDIFVSSLIAPLSNSAEEAX
>IdupSNMP_c12
KIHLFNYTNVKEFESGVVKKLNVQETGPYVYYEELEKVNVKFSKVDGTVSYQEKRN
YQFSPKLSKGSKDDLVTVPNIPLLAGAAKVKTTKFMVRLSYQTLLNVL
>IdupSNMP_c6
MWKKLVILRDQYYTGVISLAAVGLLMFCTSILMFVHNPRQSIVNMVLSLSEGSIFFNL
WTAPPYEVLLKVYMFNITNVKEFLSGKEKMNVTEIGPYVYREILTNENATVNDTDG
TVQYYPHREFSFQPDKSVGDPHMDHIMTTNIPLVGLQAFMTEKGLFPSFAFATVARS
LGSKPILNLTVHDYLWGYTDKLLTYANTLMPQWIDFDTFGIFQRLMSRDNANVATIV

NNPSKYVSNVQNLLTEEEKLAYYHIVKWNNLPGLKDWGYETLSPEEVTKCHLVEGA
FDGTIFPKNLRINQTLKIFRKAFCRPVPMVFVQDTVGKEGFRQFEYKLSDDMFDVTEE
NKCFCYKNQCMKGFQNIAPCYYDIPISLSQPHFLNAEPKMLETVHGLEPTVDKHGSF
CNIQPDAGVPLSGSMRIQVNLDVRRTIGNHQTVHFNGLQVPLFWIEITTLEPPTLVTFL
LHLLCNFMPVALEIIKYLLGLGGLALISGSALYTLVKTEVRIPGGMCLKNATEYTSIPII
SLPNEILDKC
>IdupSNMP1b
MPHPKNIALTGGTLAFGGVLFKVWLFDALVRFGVKDQTALRYRNEVRGIYLKIPFPL
NFKIYFFNVTNPEEIQNGAKPVLKEVGPYWYDEYKERVDVIDNDTEDSLTYTPYDLF
KFNRNKSTPLSDNDYVTIIHPVIVGMVNLLLRESPMLLKVVSKAIPYIFNDPKTIFLTG
RVKDILFEGVVLNCTSKEFASTAVCGQMKGQVPGLKSTPGQPNLLLFSLLGPRNATA
TGSVKVLRGIKHYQDLGRLLEVNGKKSVGIWGSDQCNRYDGTDSWIFPPLIKPEDGL
KSFSTDLCRNIKMKLVNETVVKKIPIKVFETNLGDQSRDQEEKCYCRDTCLKKGVFD
LTKCMGVPLYATLPHFLDTDEDYLKLVDGLKPVHDRHRIVVFFETLTGTPLIAAKRM
QFNLELQQTNKLELFSKLPTALFPIFWLEEGMELNGYLLKKIQTVFMLLLFAEVTIYA
VIASGLGVCGTGCYLYWKNTKSLSITPLSKEKIADNKENGLNTILNEPKLYX
>IdupSNMP2b
MAALQLTSGIQRLALAGCLDRILGPTGMDQVFIKKPVKDILFEGIPFAMANQSNLGY
ACSLIRTKMINITESVRVIEHVEDEGDYLKLAIFNYKTSNYLKTFPDGLYKVNRGIEN
ASALGTIMQWNQNTFIPTYGNAWTASNDSCHTLRGTDSTIYPPKAKEGEGLYIYNTD
ICRTVKLAFKSSEESYSGIKAFRYETEDTFLKTSSLIPEHDCFCTKSTKDASGQDNCYL
DGAMDFKPCLGAPALISQPHFLNADDSYLEGVTGLNPDKEKHGIYLLLEPNTGTPLE
GMKRIQINTVLRSQPLINMTTPTTMHNGIFPVLWLEEGFSLPEEYVDKLNNQYFKAV
KIATGLKYALIAVTSAFFIACIYLVWRKEYFKKX

Supplementary information 2. Predicted transmembrane regions from IdupORs.

The transmembrane regions were predicted using TOPCONS [101]. And the results show TM regions from 67 IdupORs. The boxes indicate transmembrane regions, whereas red and blue lines indicate intracellular and extracellular regions, respectively.

IdupOR19a

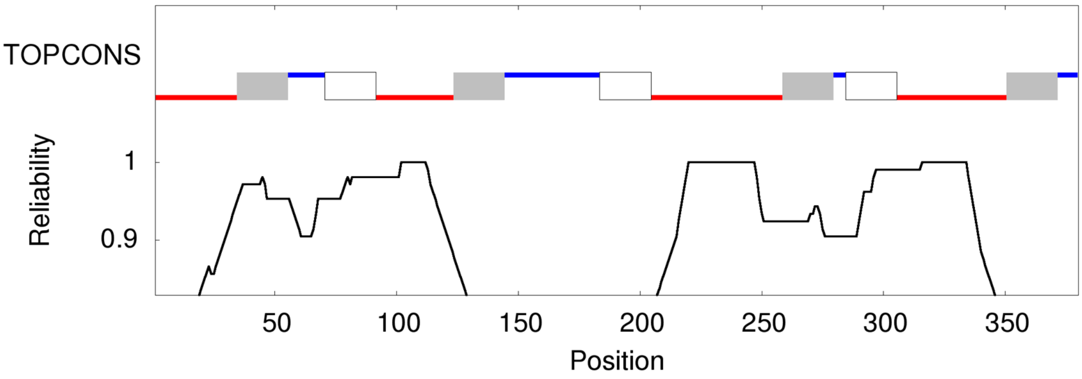

IdupOR13

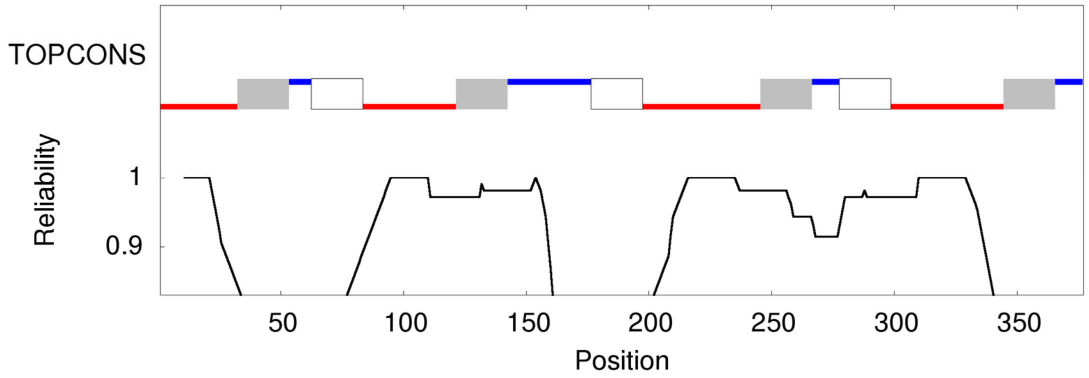

IdupOR12b

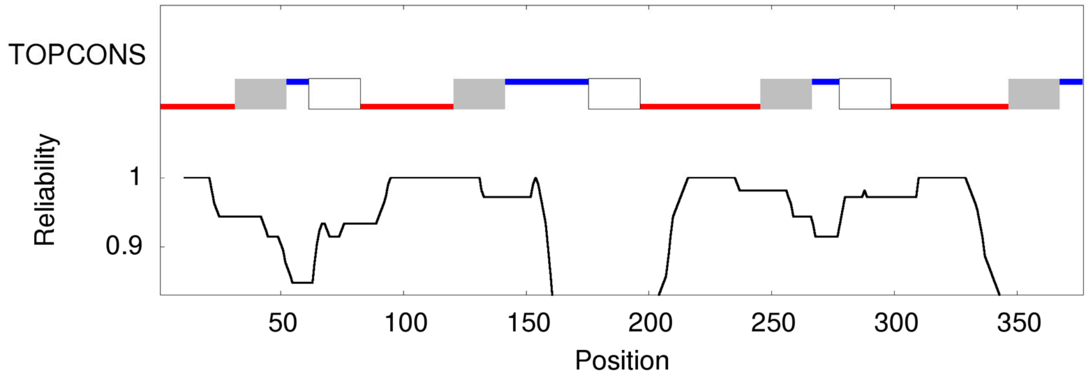

IdupOR16

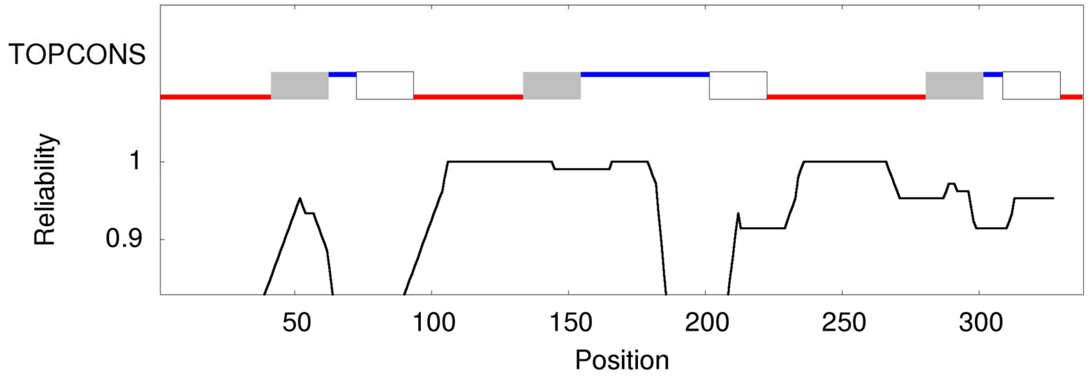

IdupOR17

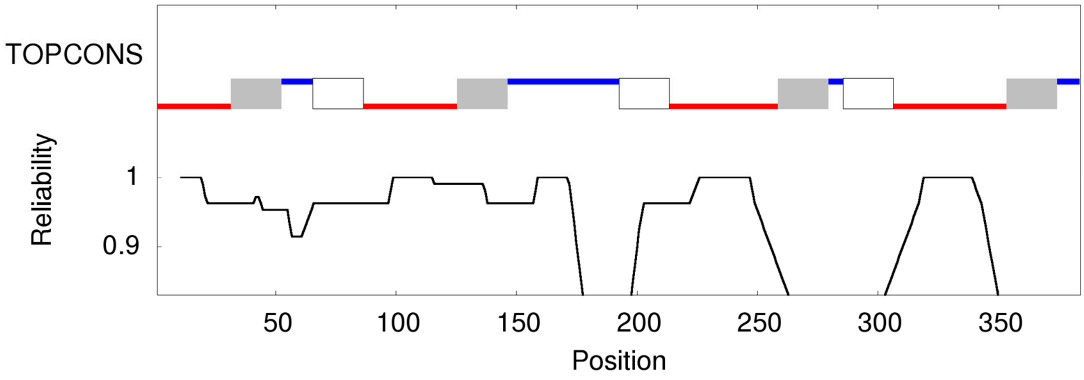

IdupOR11

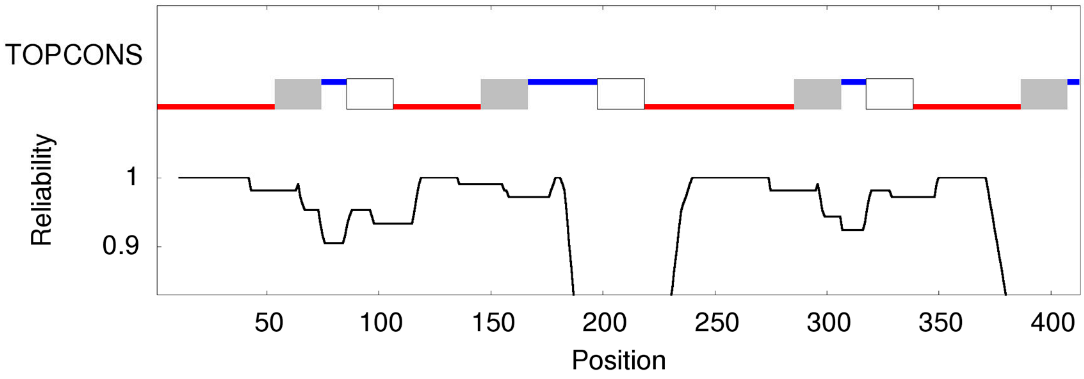

IdupOR12a

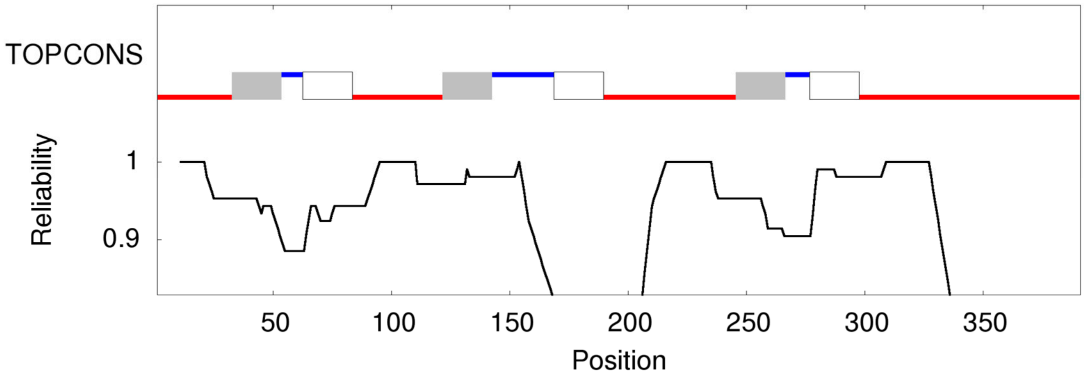

IdupOR20b

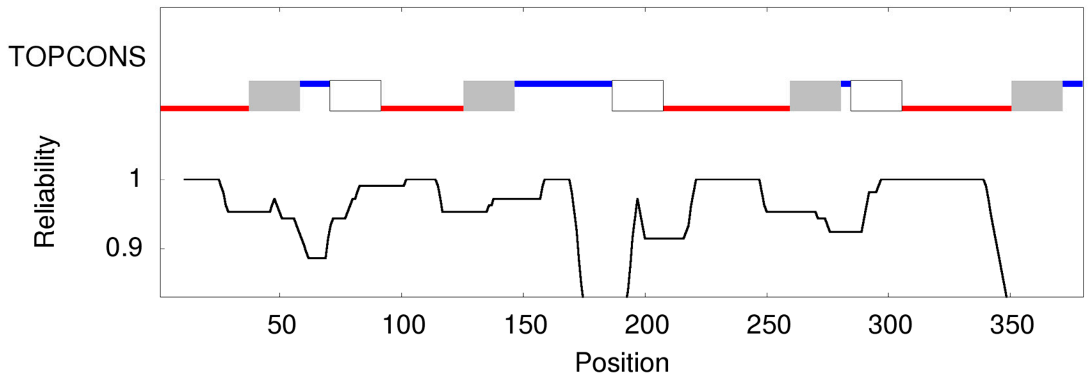

IdupOR3

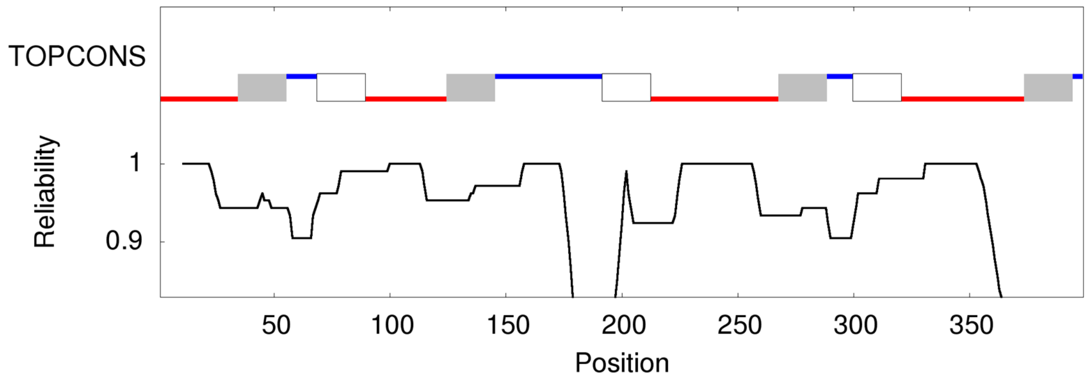

IdupOR32a

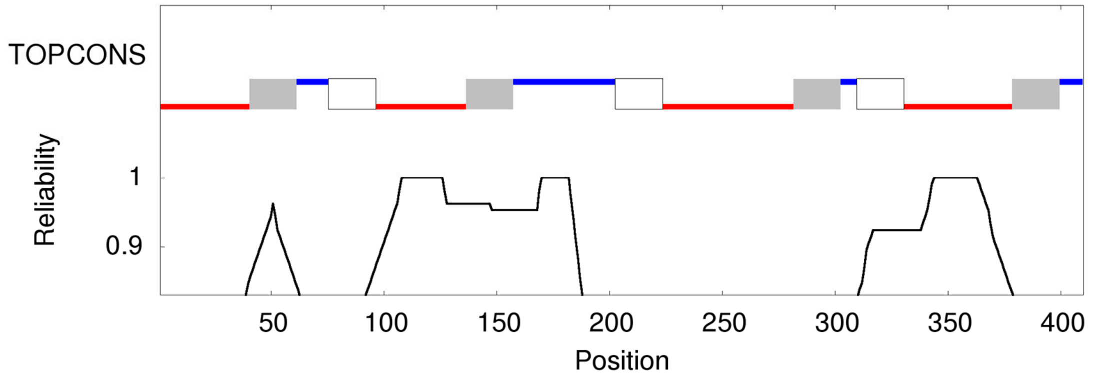

IdupOR31

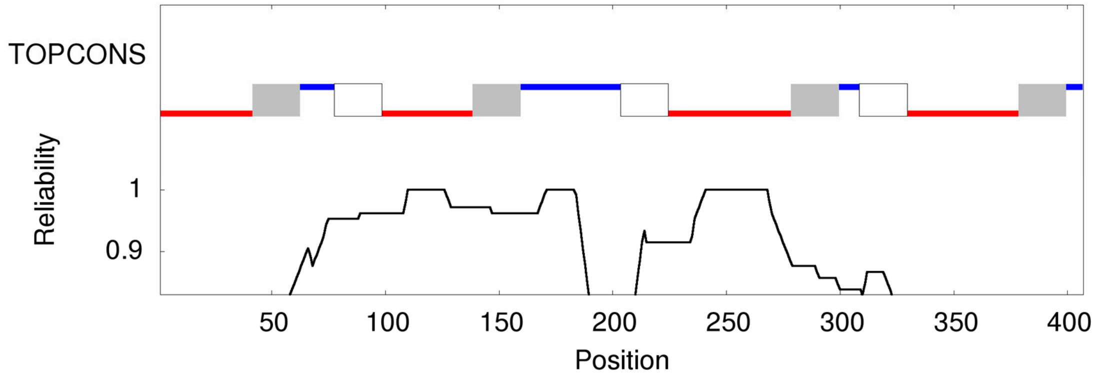

IdupOR2a

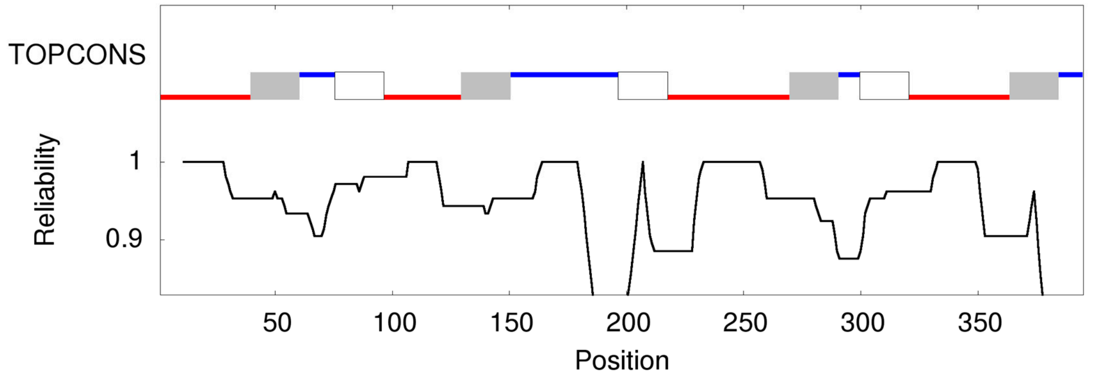

IdupOR2b

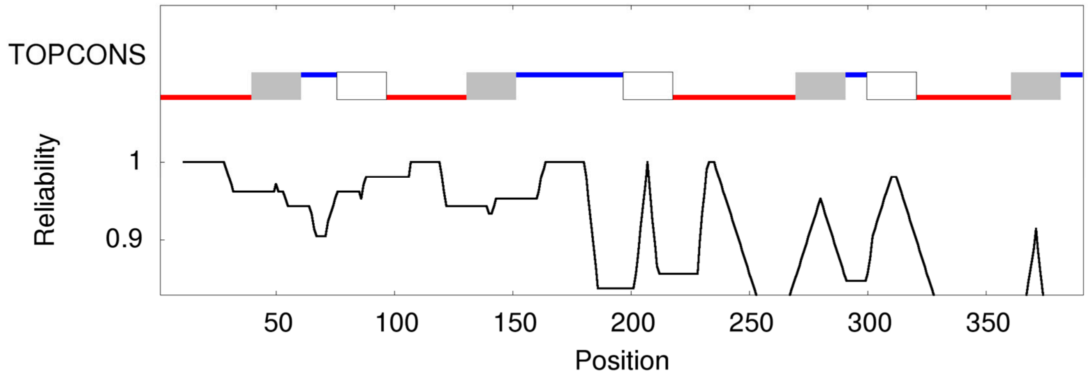

IdupOR23

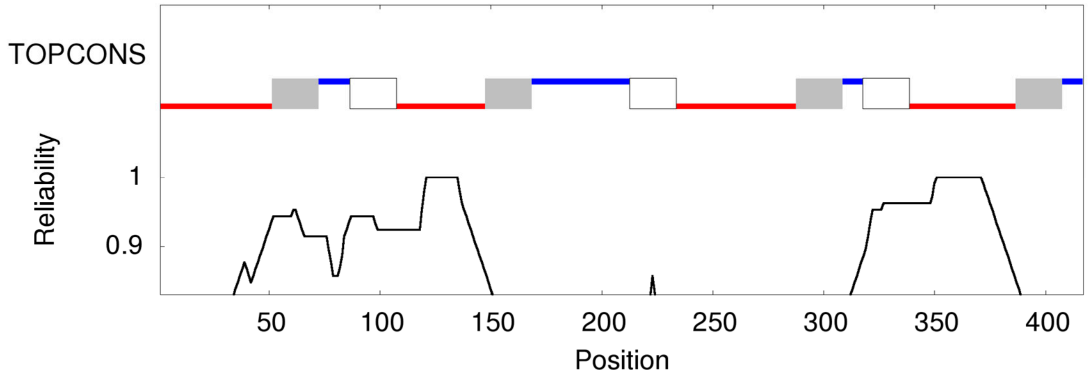

IdupOR27

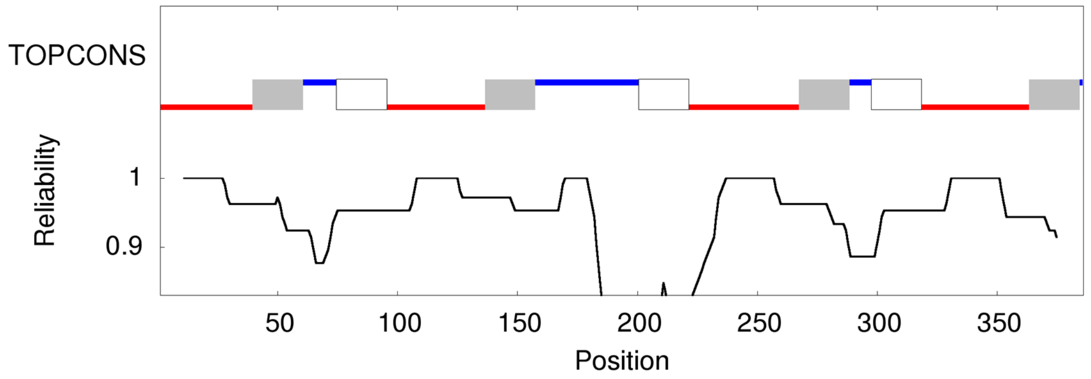

IdupOR29

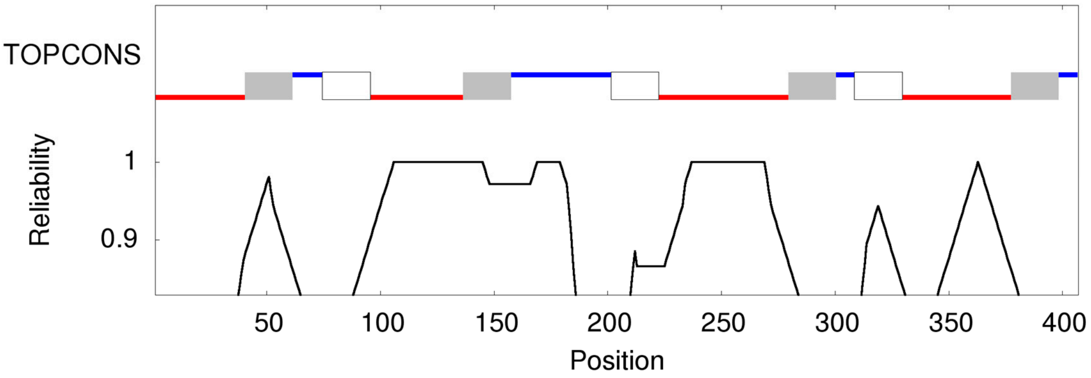

IdupOR28

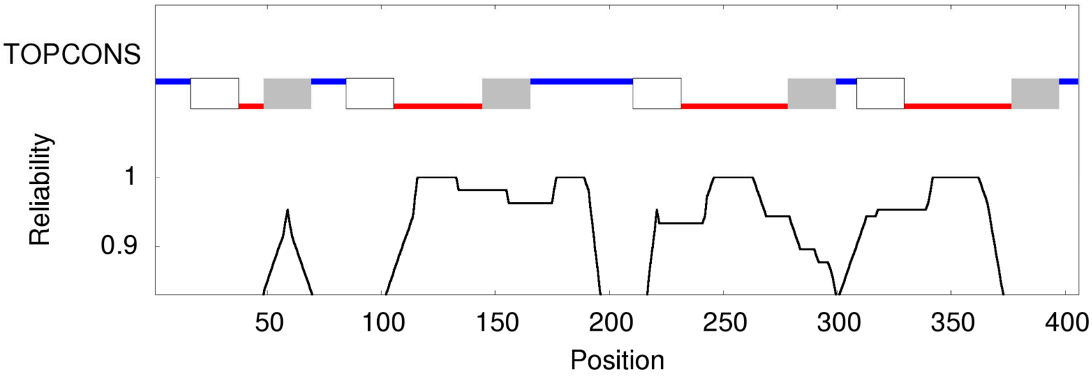

IdupOR25

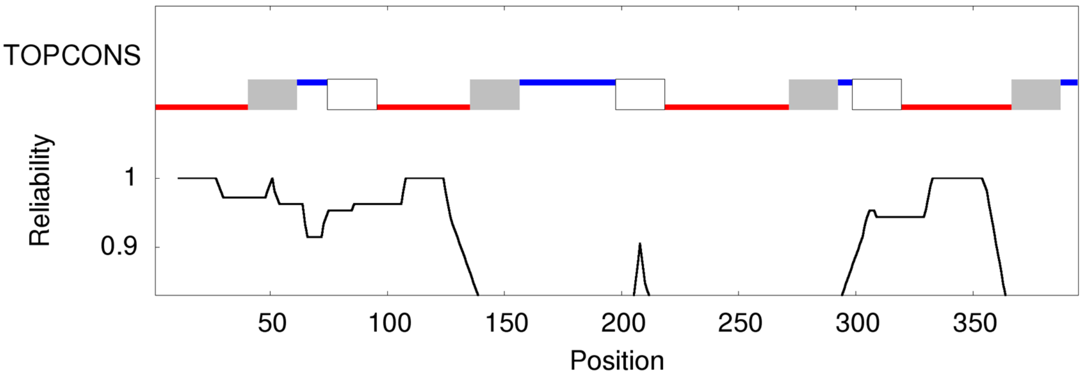

IdupOR22b

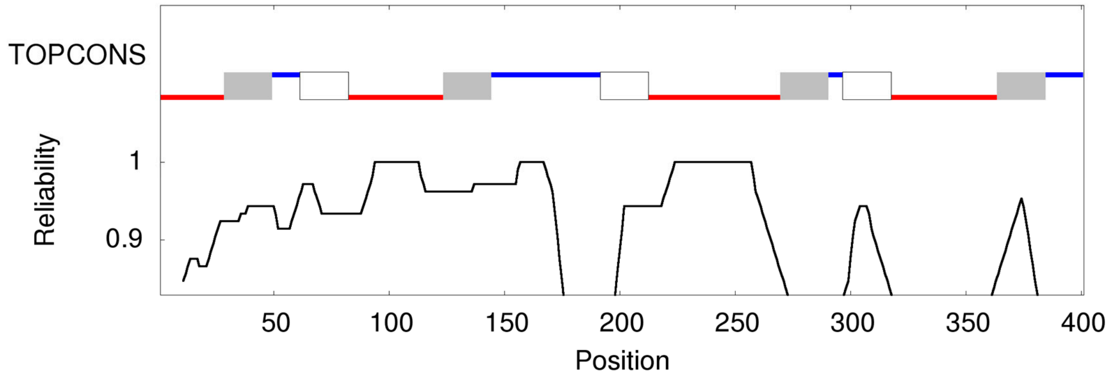

IdupOR35b

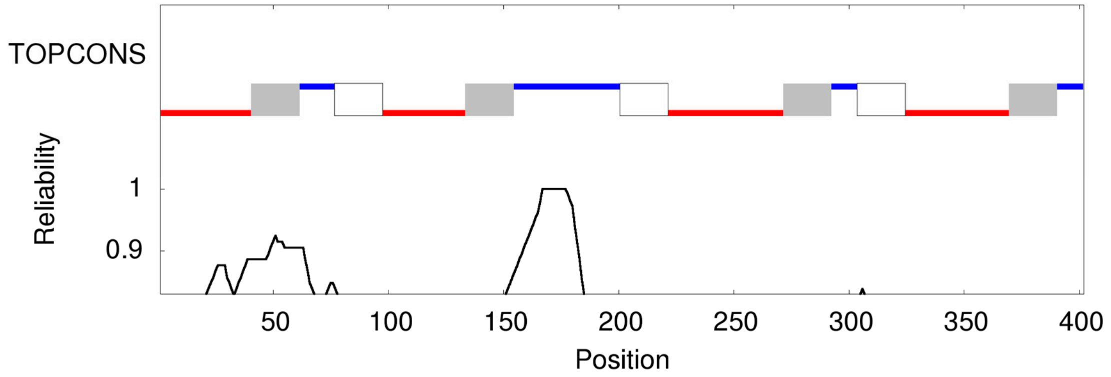

IdupOR36

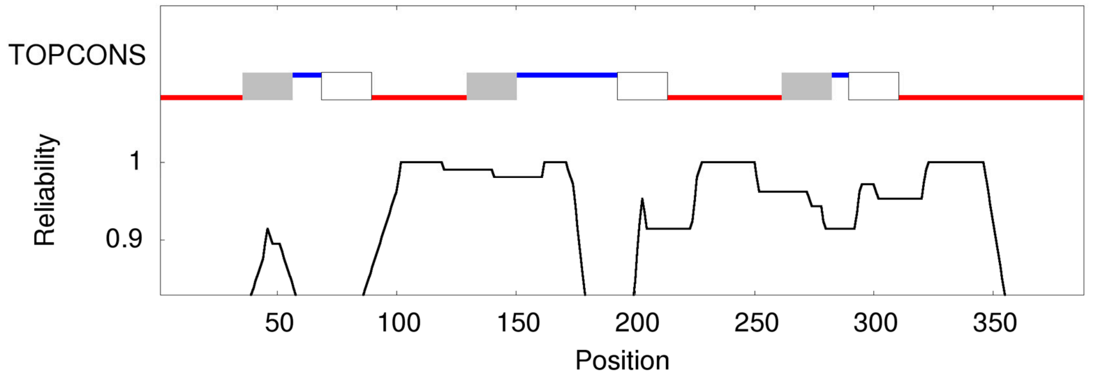

IdupOR37

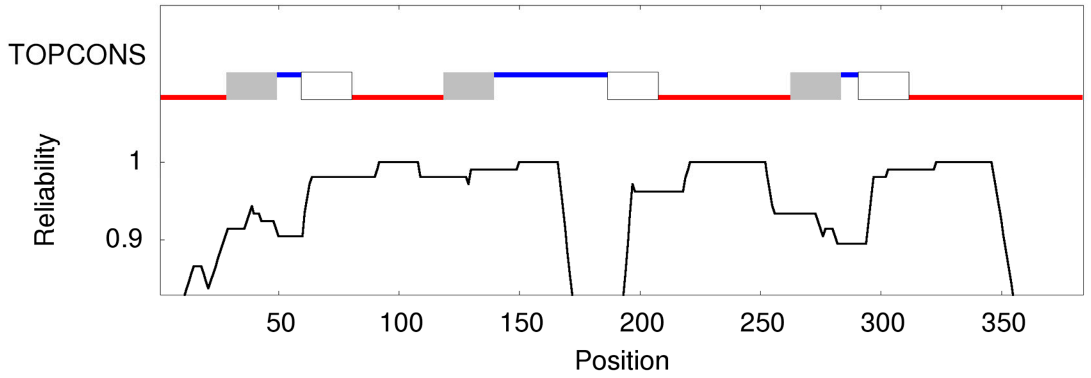

IdupOR40

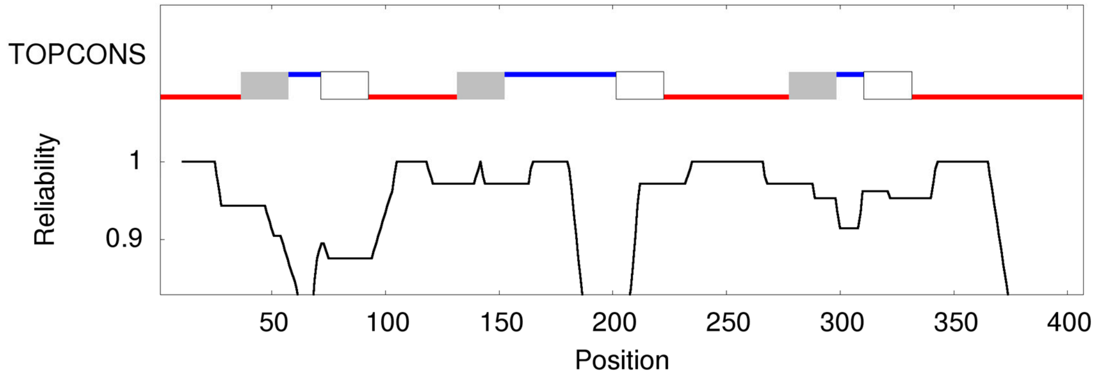

IdupOR44a

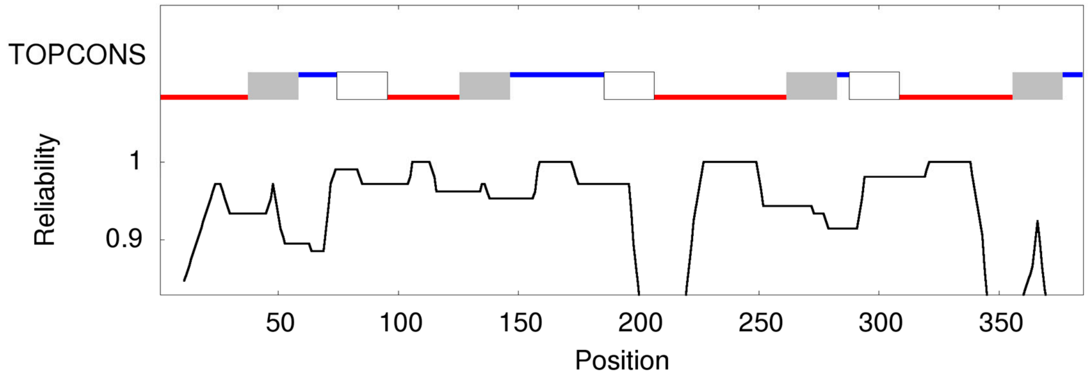

IdupOR45

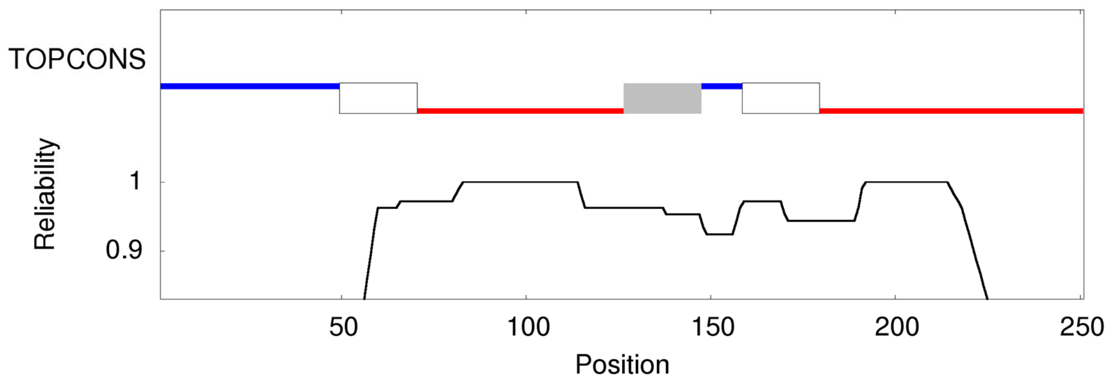

IdupOR46

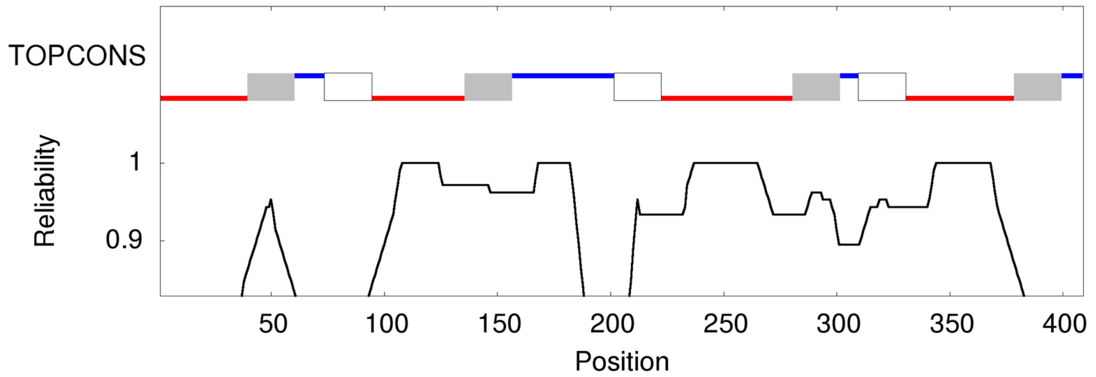

IdupOR47a

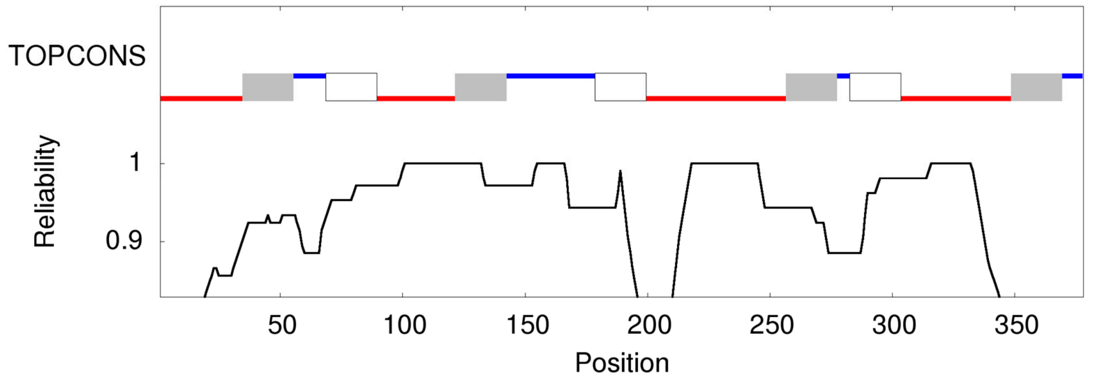

IdupOR47b

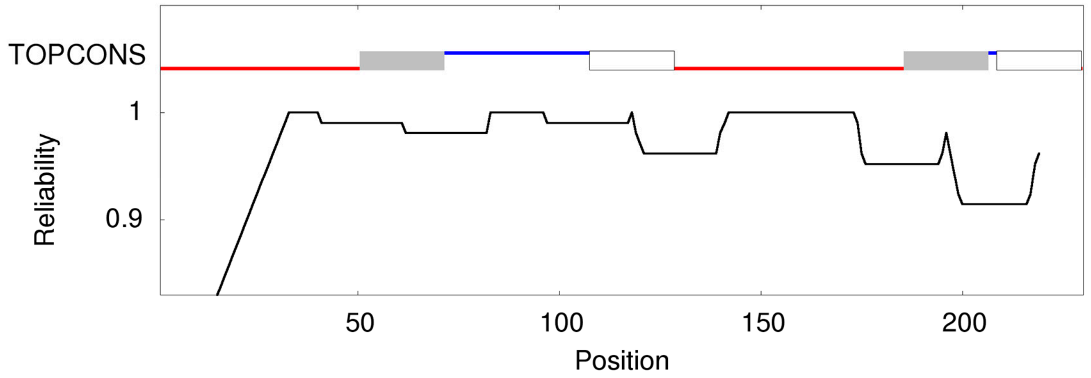

IdupOR50a

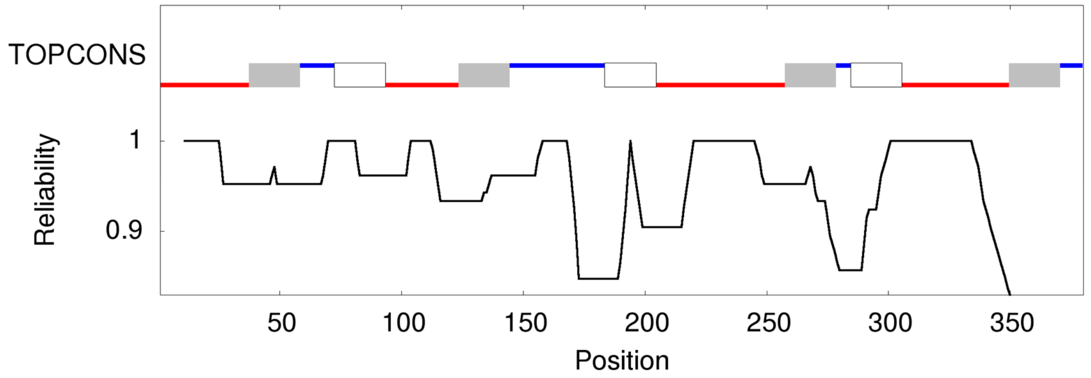

IdupOR50b

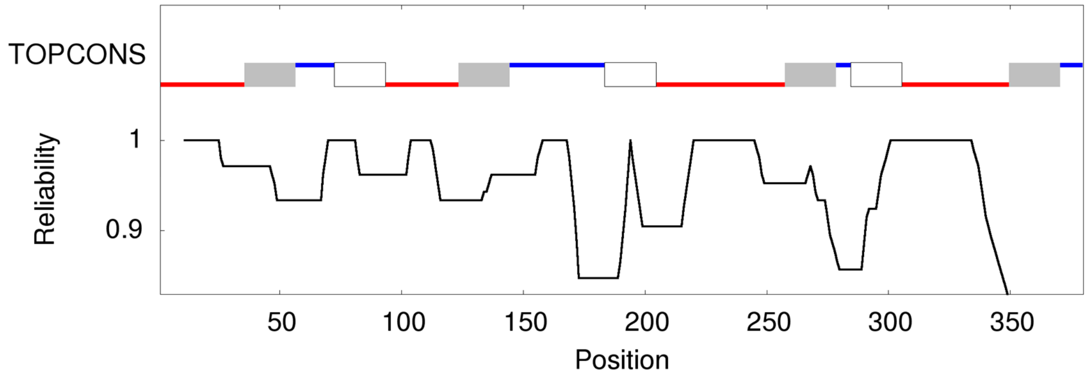

IdupOR51

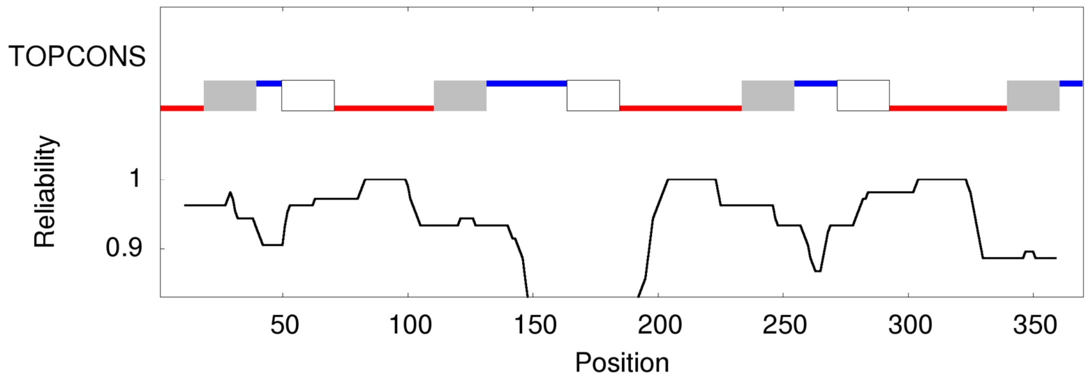

IdupOR51

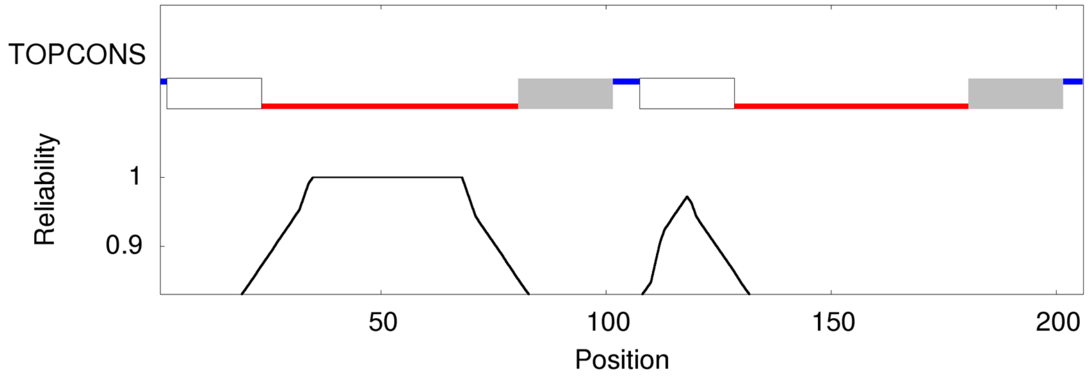

IdupOR53

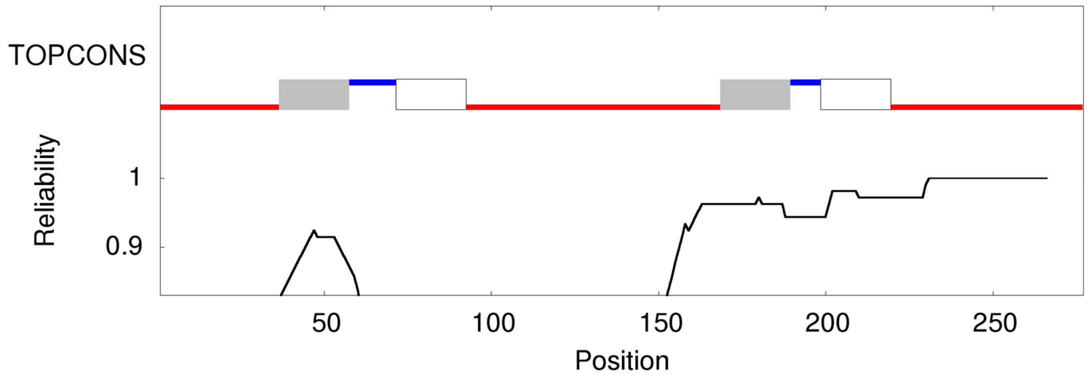

IdupOR54a

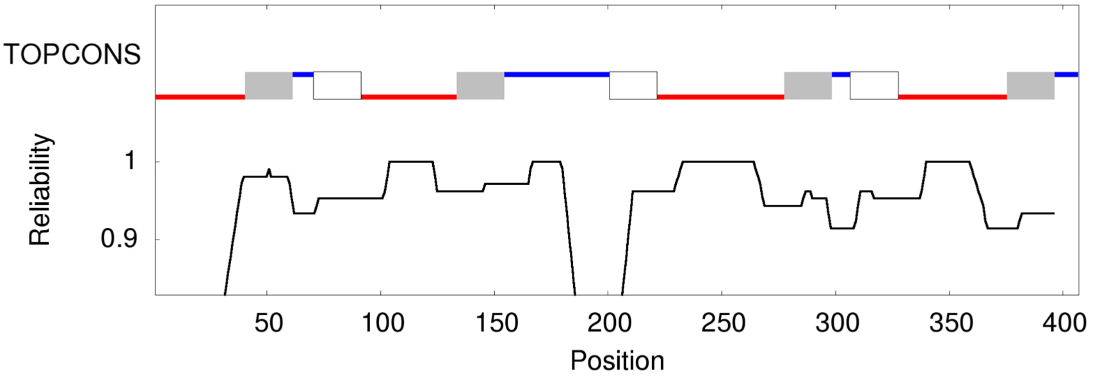

IdupOR54b

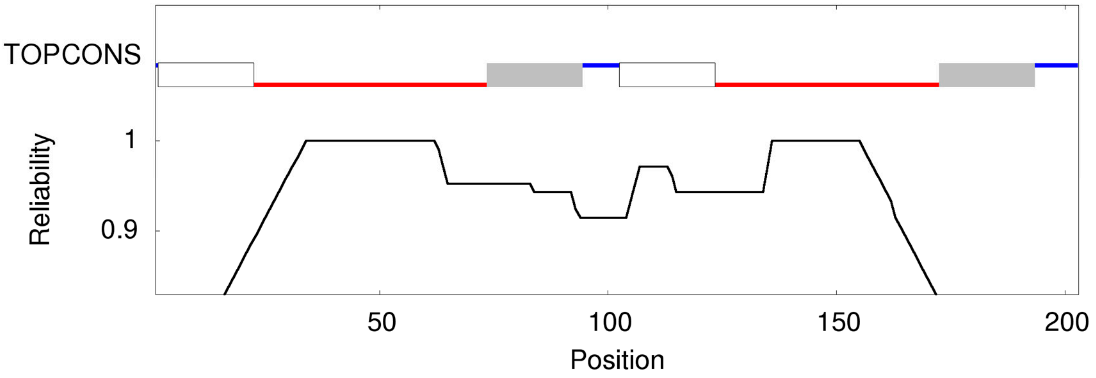

IdupOR4b

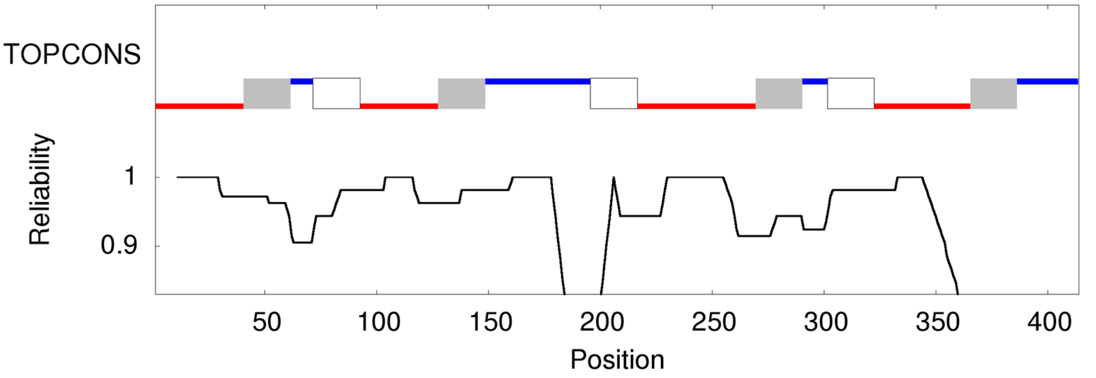

IdupOR56

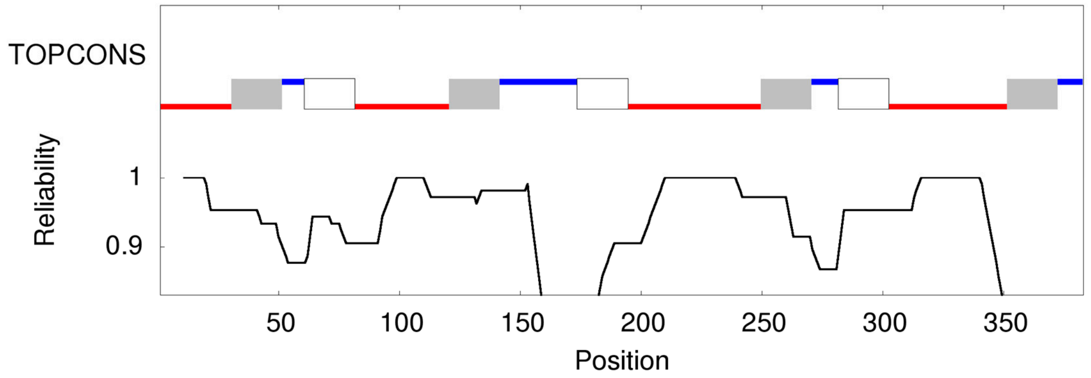

IdupOR49

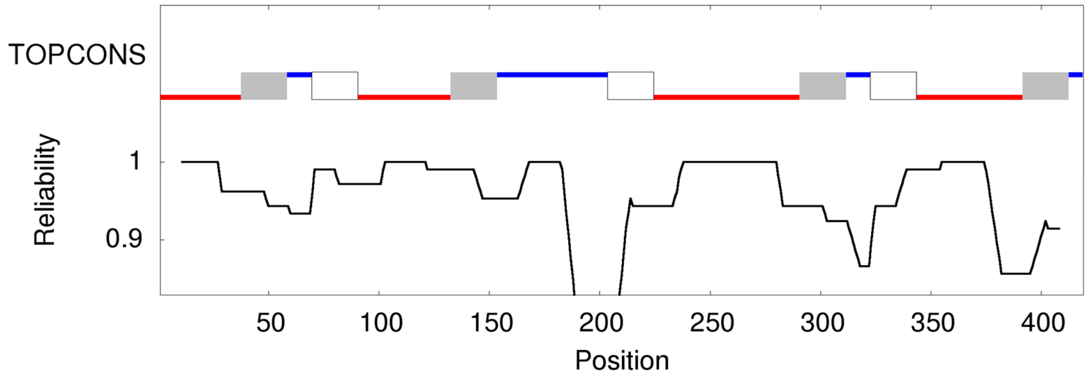

IdupOR5

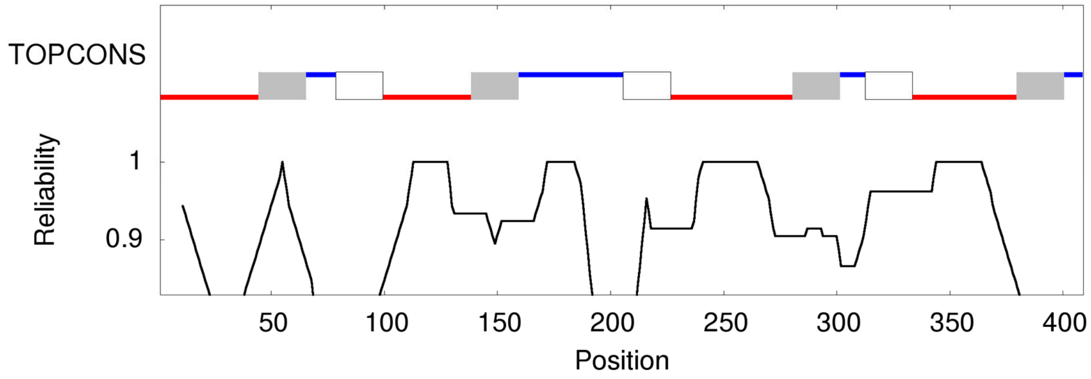

IdupOR48

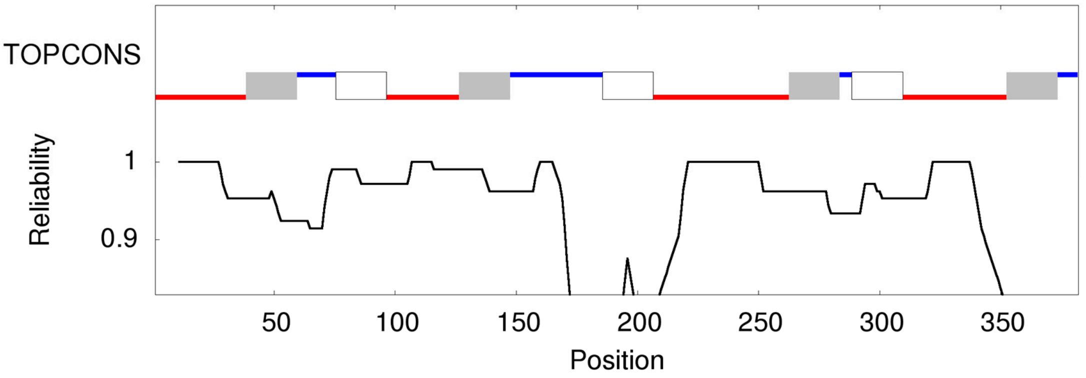

IdupOR44b

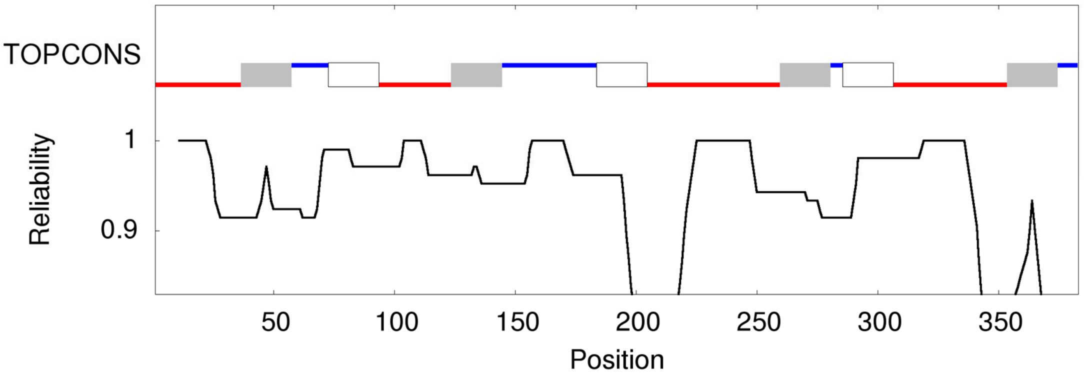

IdupOR4a

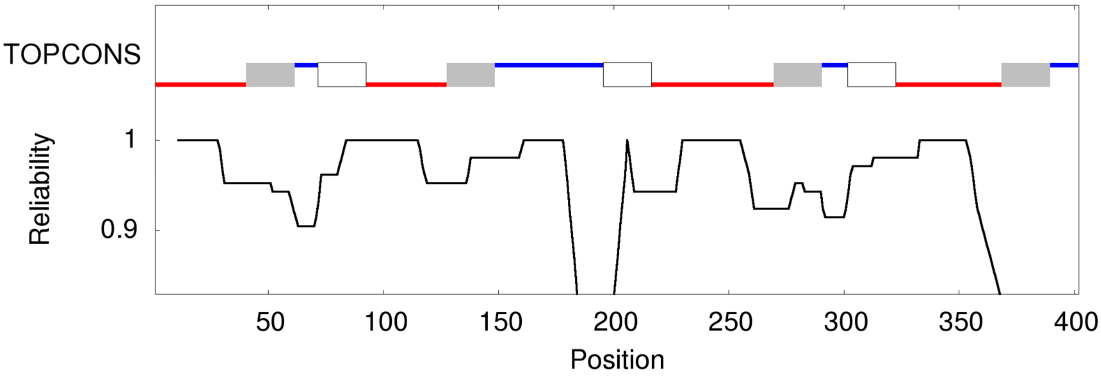

IdupOR64

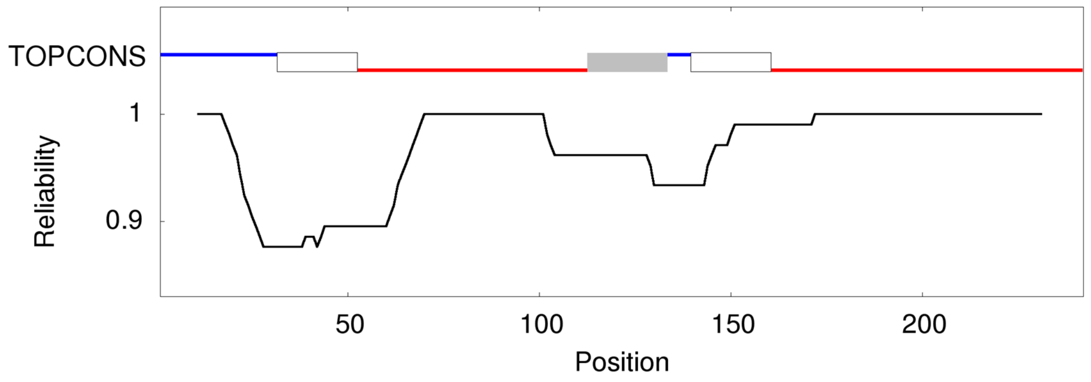

IdupOR9

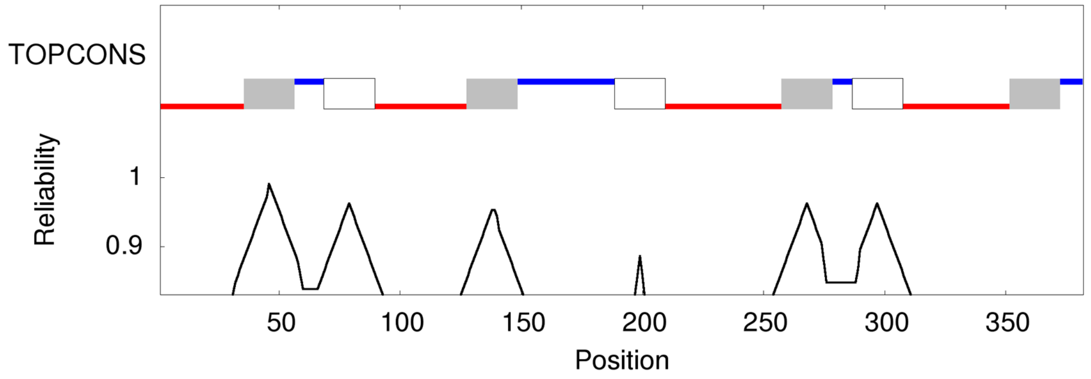

IdupORCo

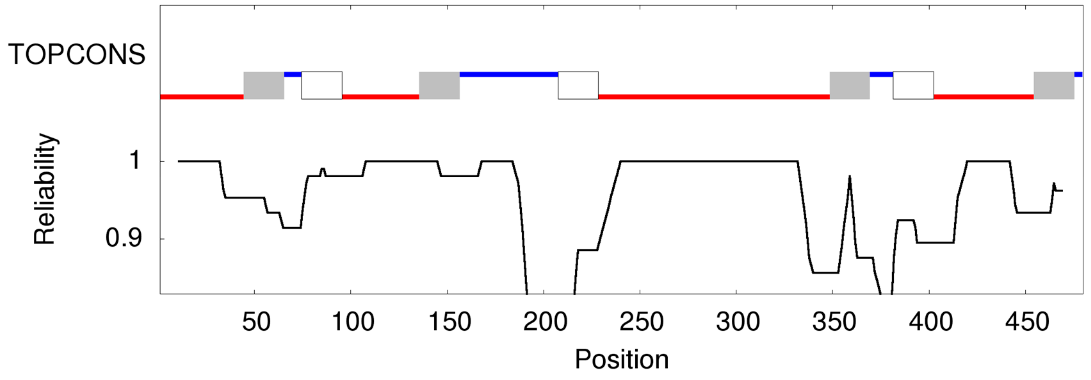

IdupOR33

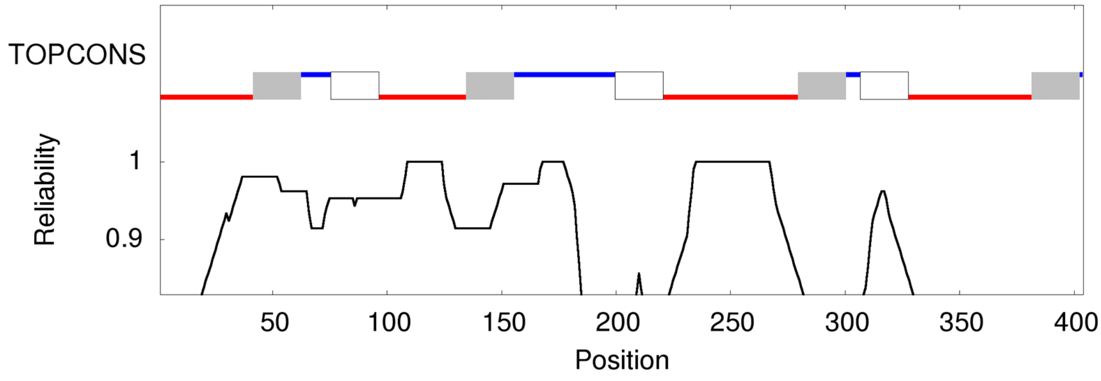

IdupOR19b

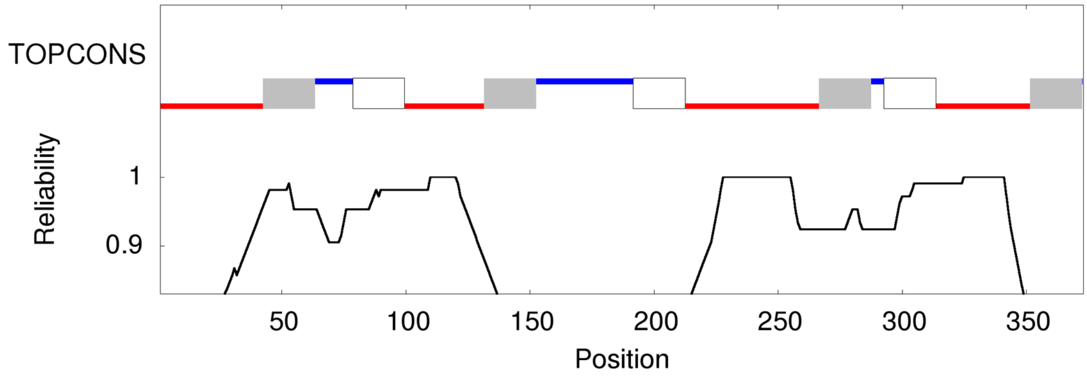

IdupOR1

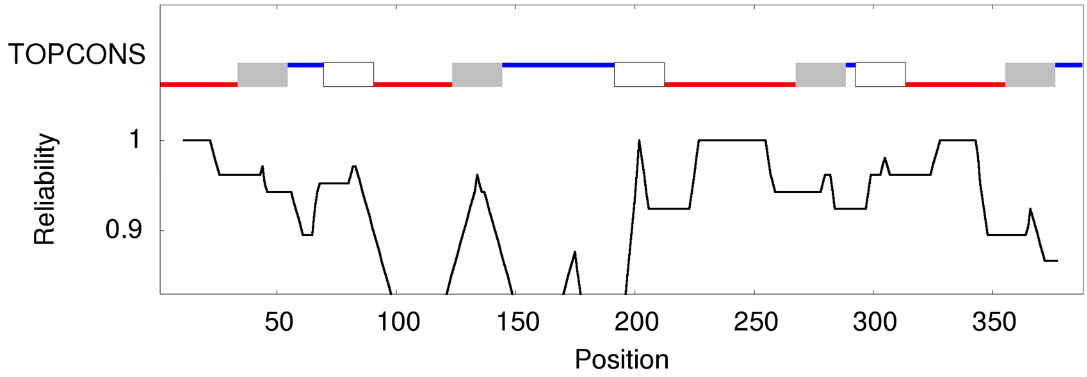

IdupOR20a

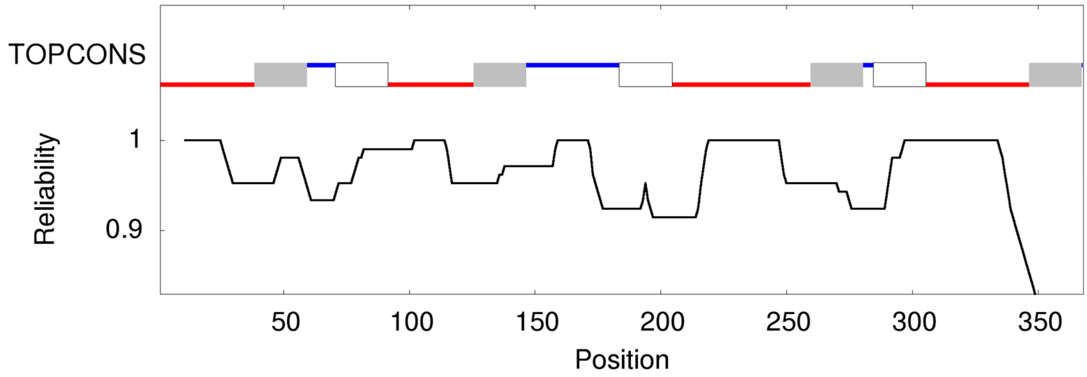

IdupOR61

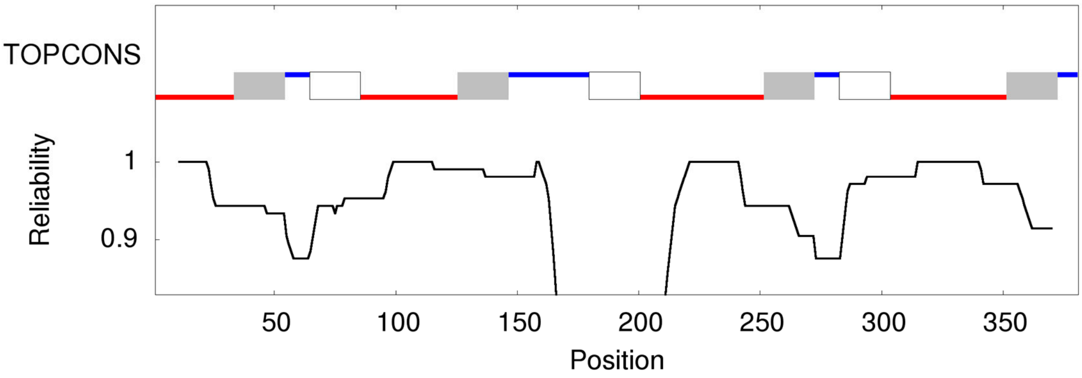

IdupOR60b

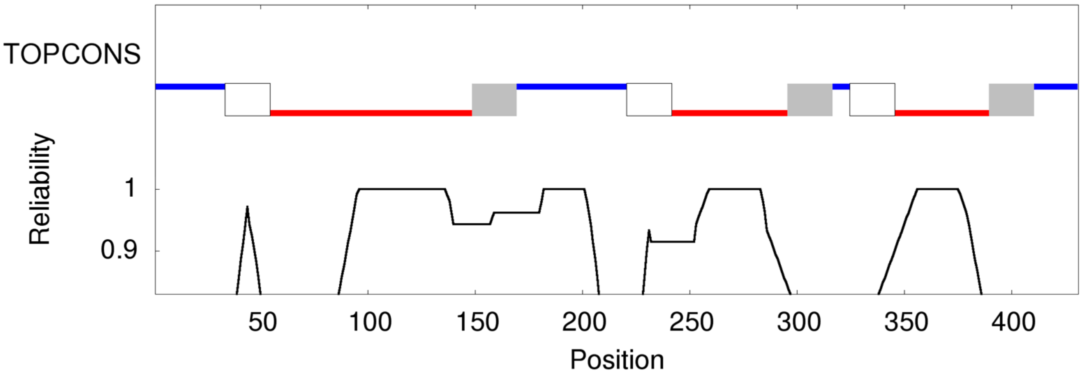

IdupOR60a

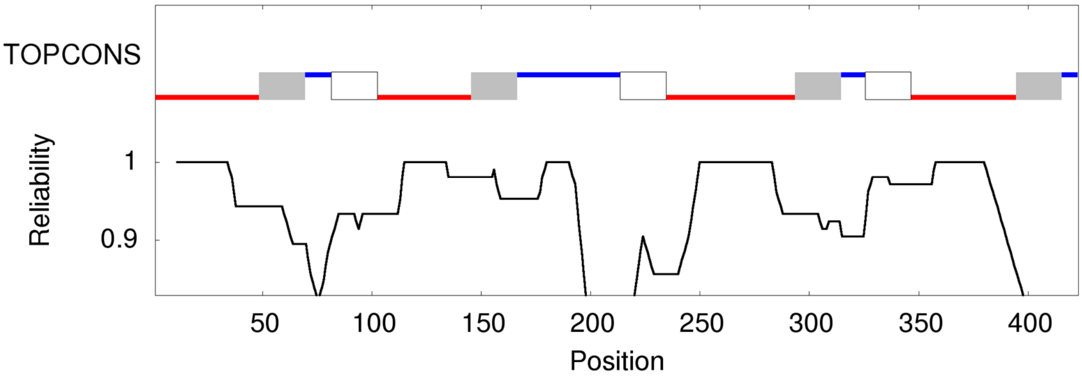

IdupOR6

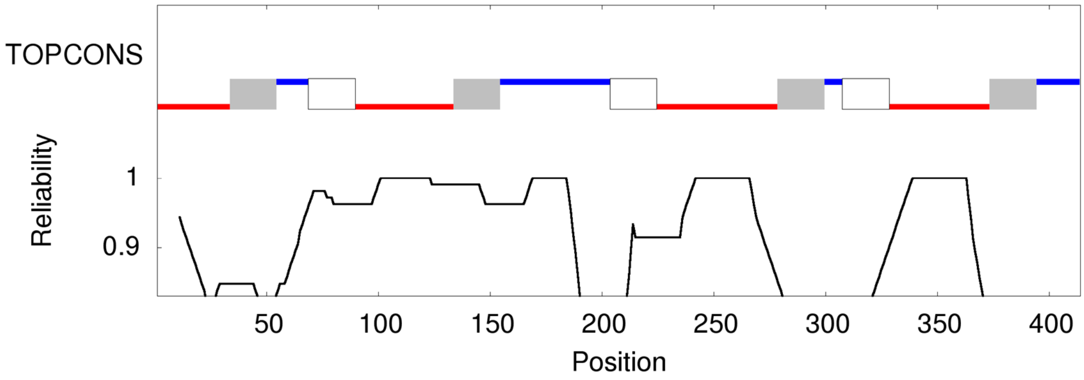

IdupOR10

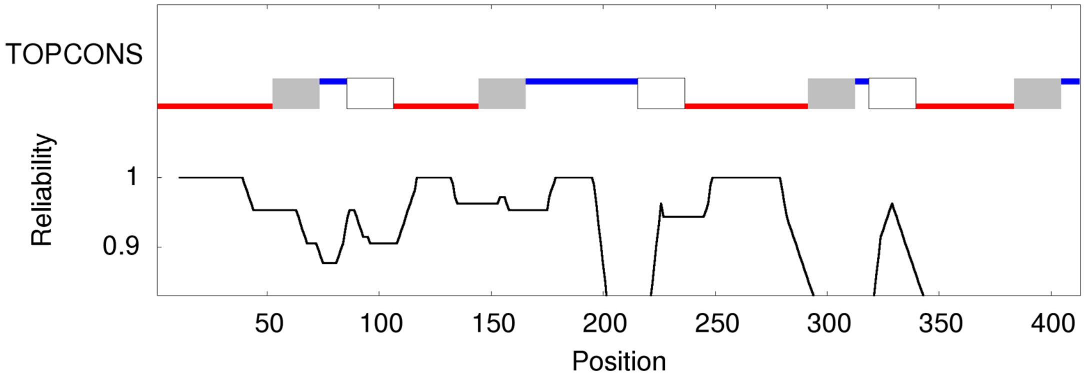

IdupOR34

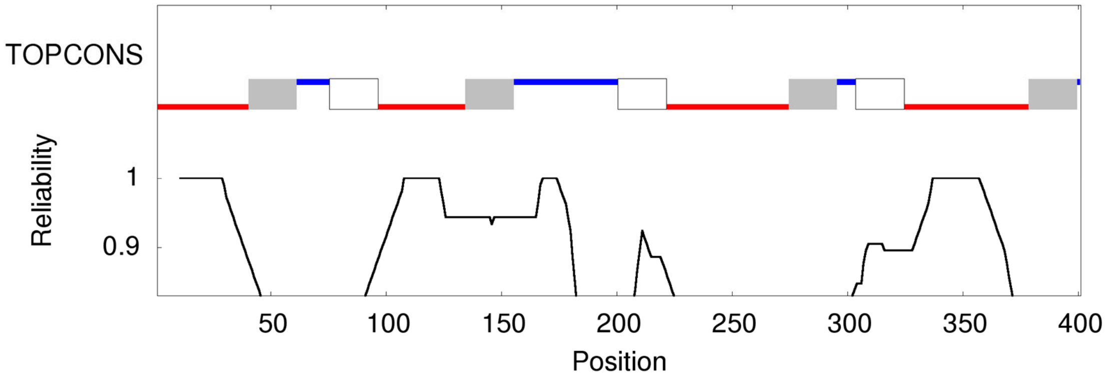

IdupOR43

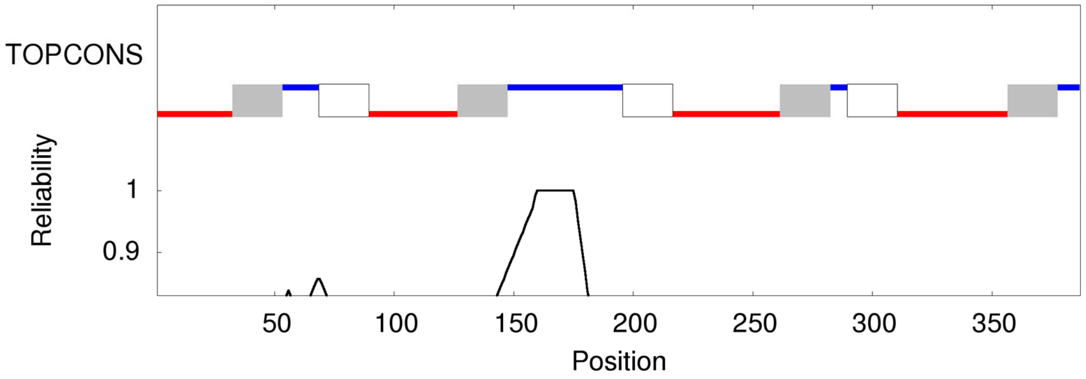

IdupOR22a

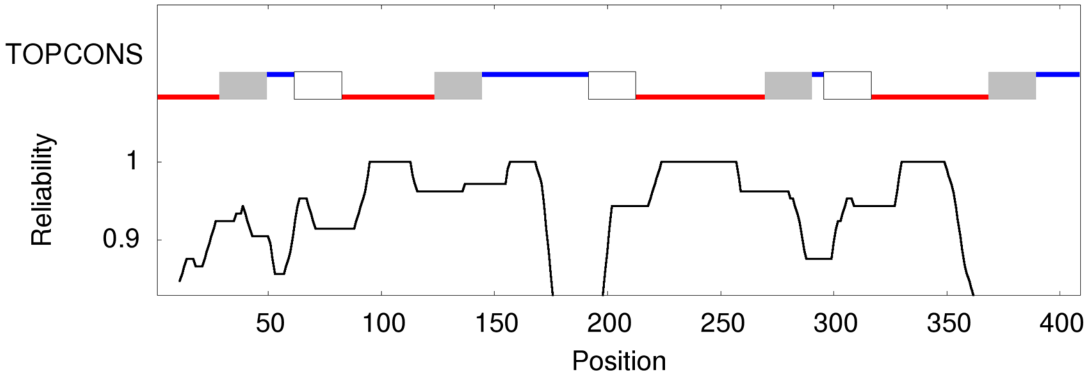

IdupOR21

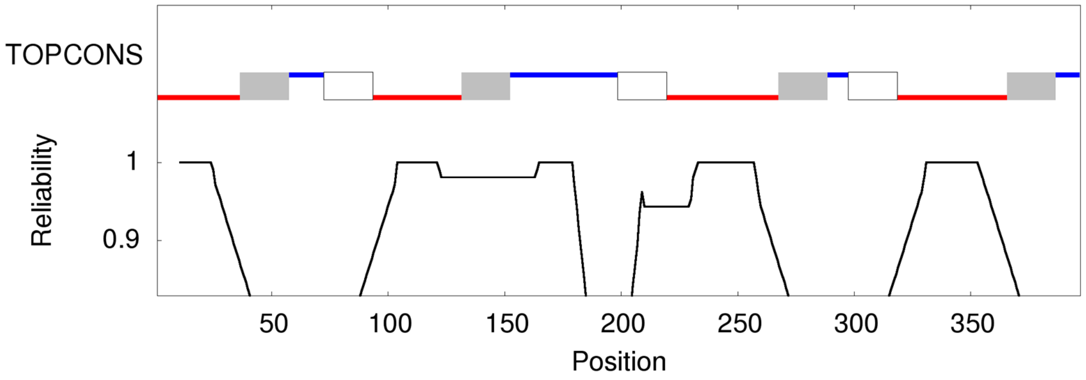

IdupOR35a

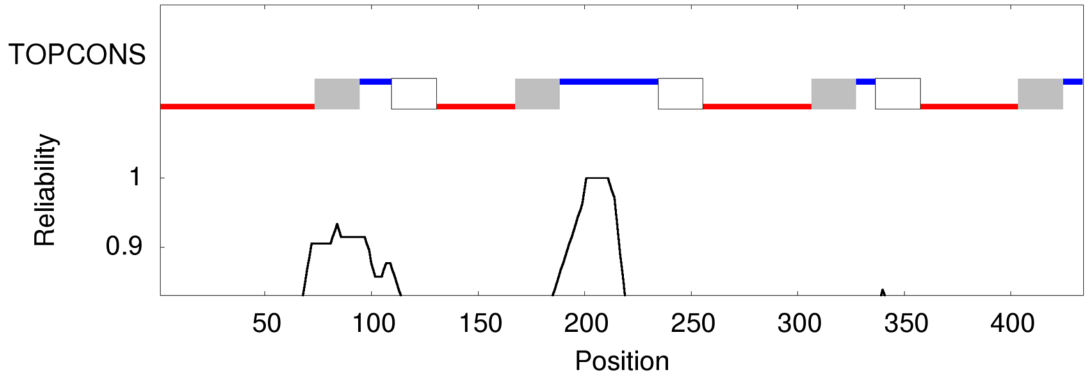

IdupOR41

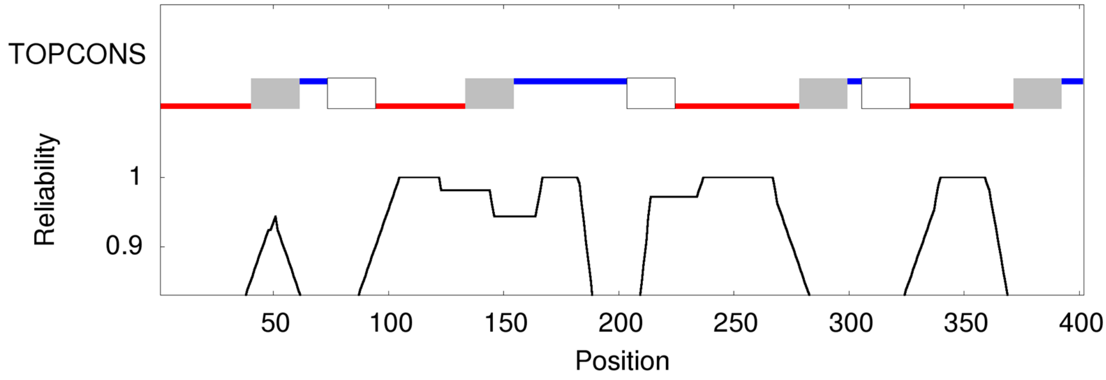

IdupOR32b

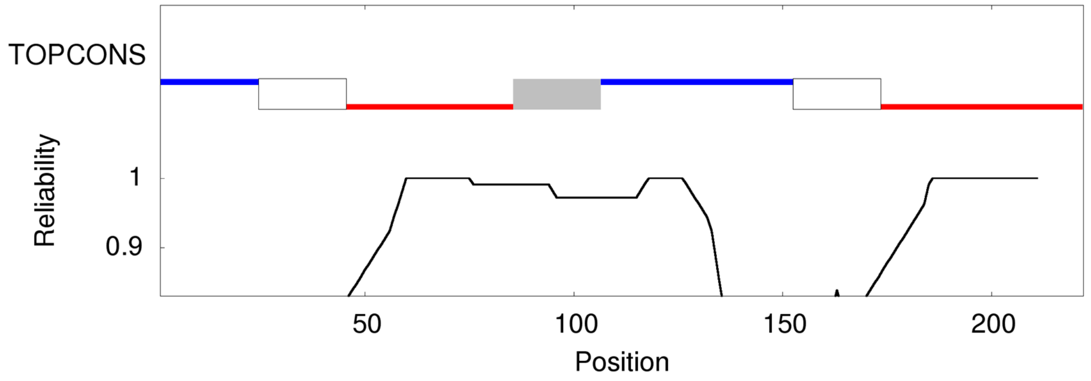

IdupOR73

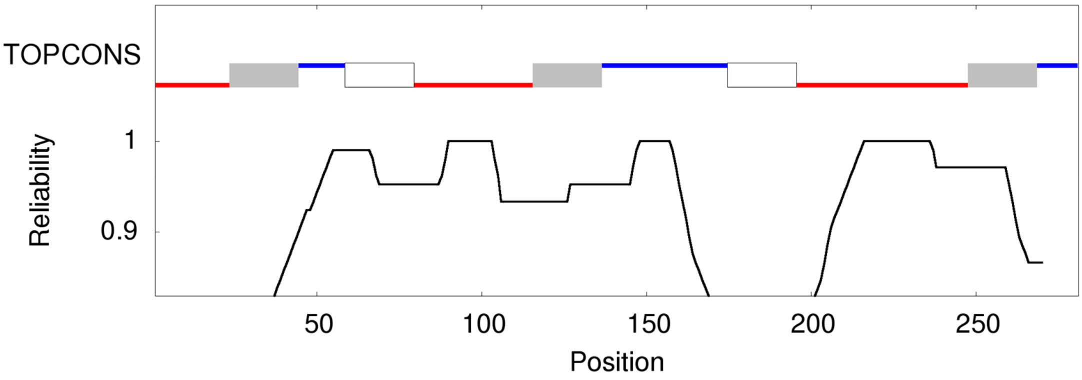

IdupOR76

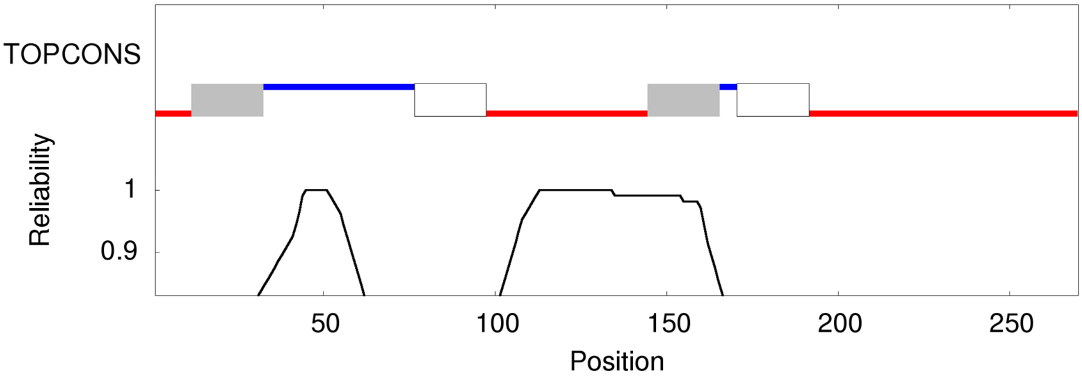

IdupOR77

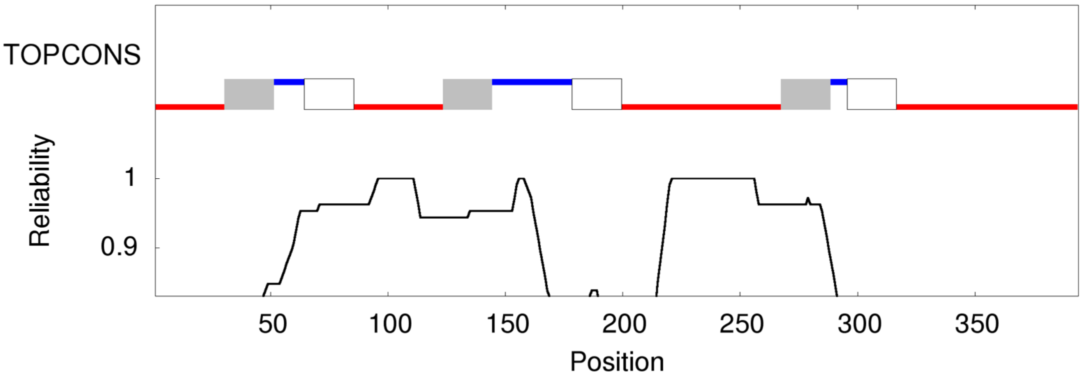

IdupOR66

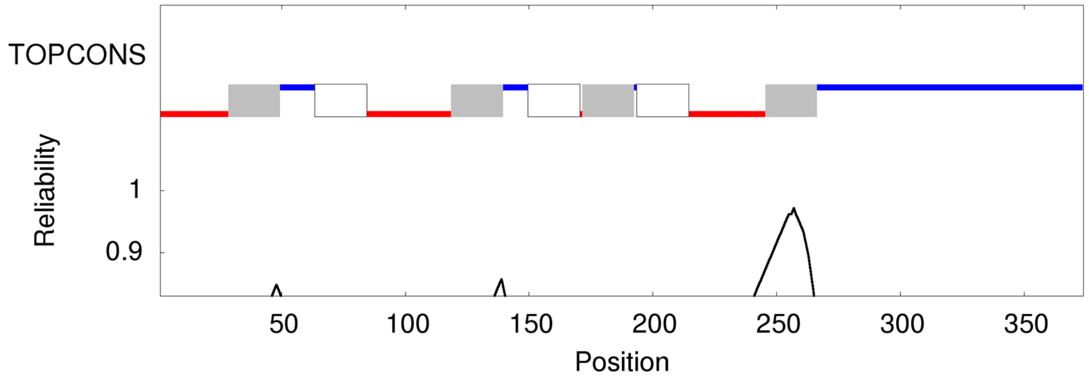

Supplementary information 3. Predicted signal peptides in IdupOBPs

The signal peptides were predicted using SignalP-6.0. [115] The result shows 18 IdupOBPs identified with signal peptides and the exact cleavage position. Prediction: Signal Peptide (Sec/SPI)

IdupOBP1

Prediction: Signal Peptide (Sec/SPI). Cleavage site between pos. 23 and 24. Probability 0.977640

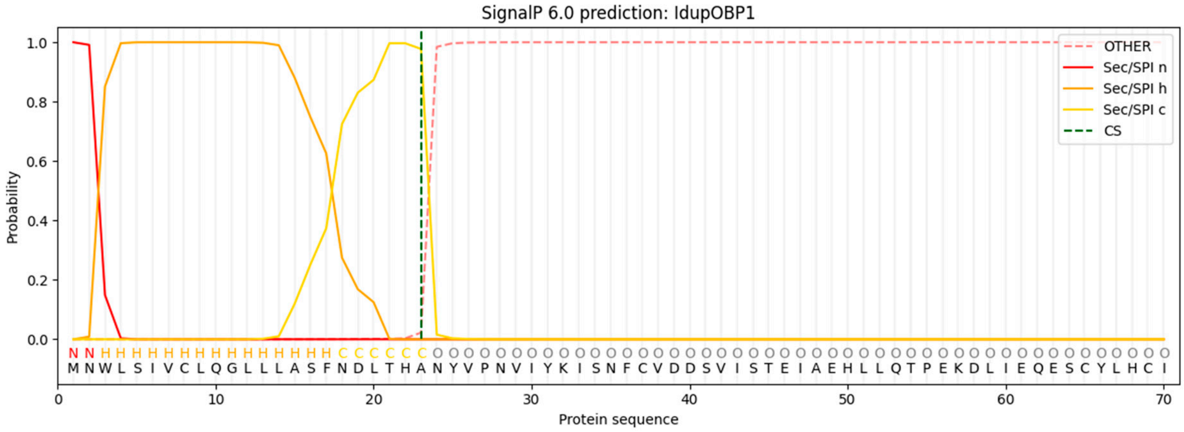

IdupOBP2

Prediction: Signal Peptide (Sec/SPI)

Cleavage site between pos. 16 and 17. Probability 0.977379

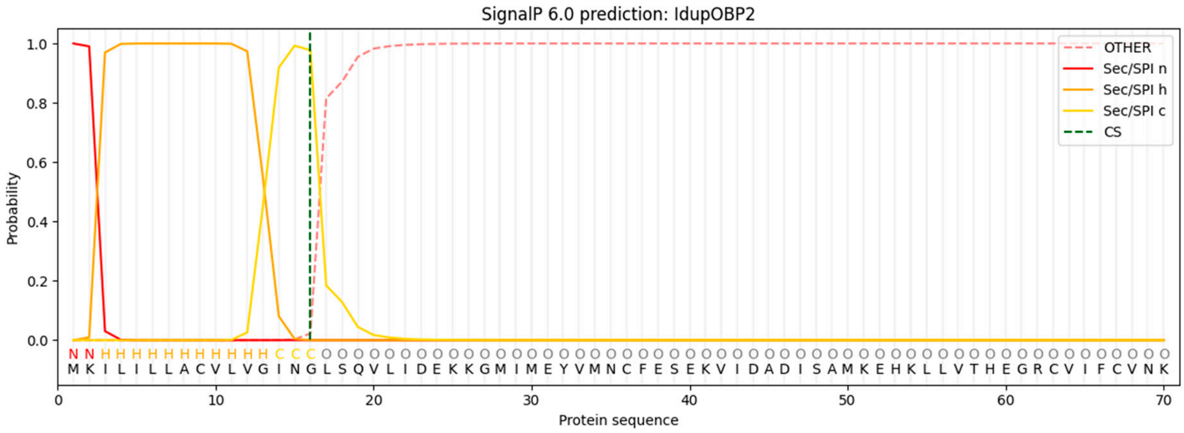

IdupOBP3

Prediction: Signal Peptide (Sec/SPI)

Cleavage site between pos. 20 and 21. Probability 0.977454

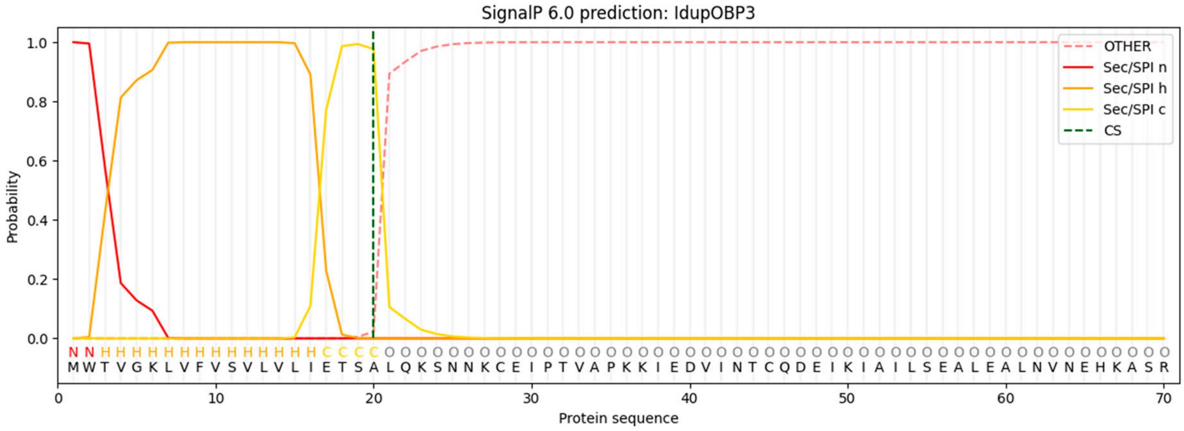

IdupOBP6

Prediction: Signal Peptide (Sec/SPI)

Cleavage site between pos. 17 and 18. Probability 0.983791

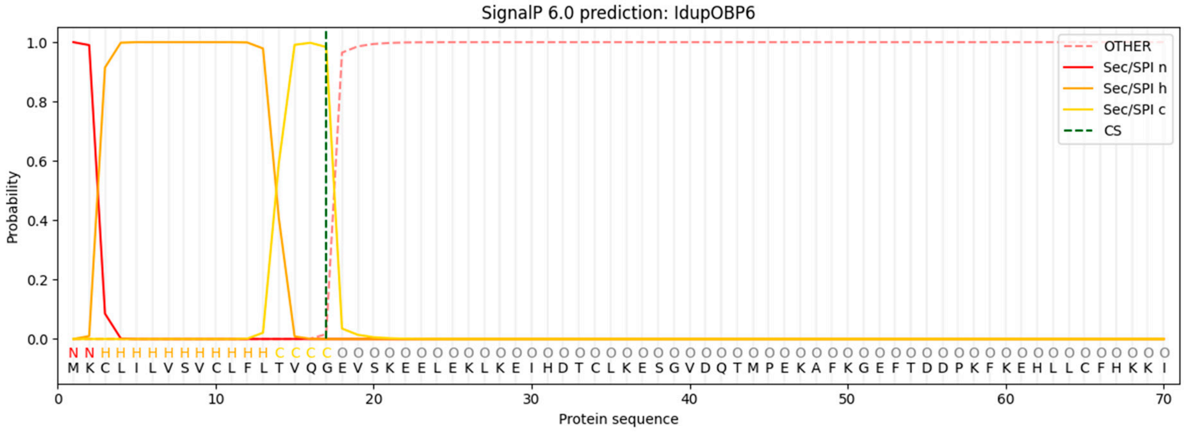

IdupOBP7

Prediction: Signal Peptide (Sec/SPI)

Cleavage site between pos. 18 and 19. Probability 0.967637

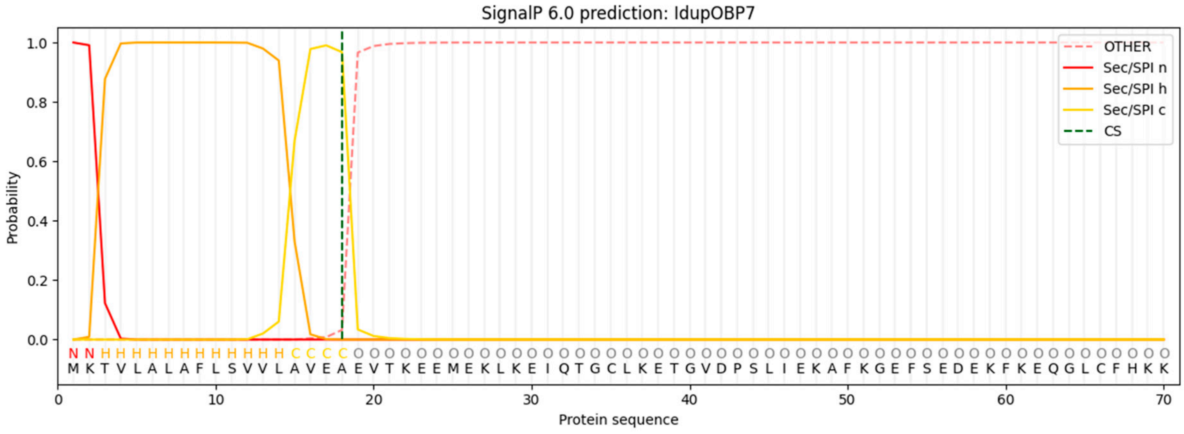

IdupOBP8

Prediction: Signal Peptide (Sec/SPI)

Cleavage site between pos. 14 and 15. Probability 0.870208

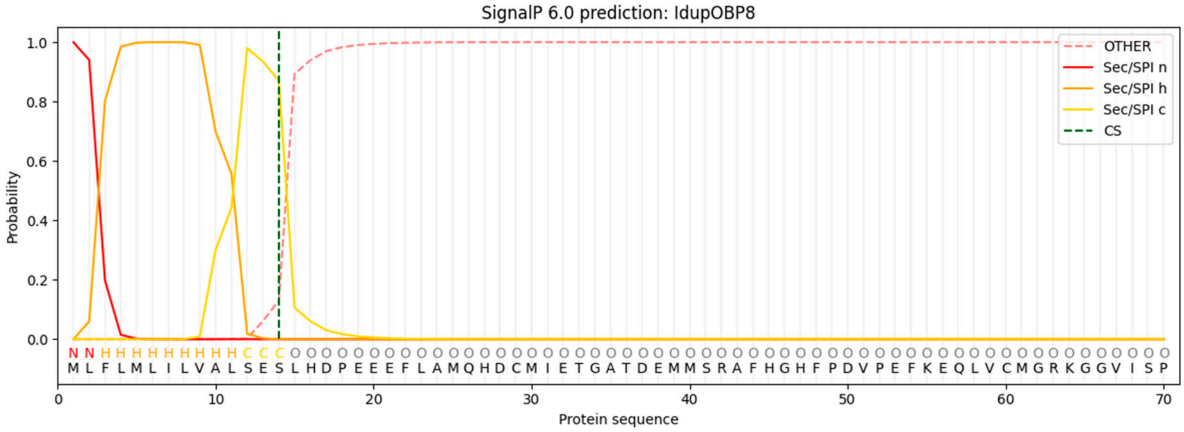

IdupOBP9

Prediction: Signal Peptide (Sec/SPI)

Cleavage site between pos. 17 and 18. Probability 0.981017

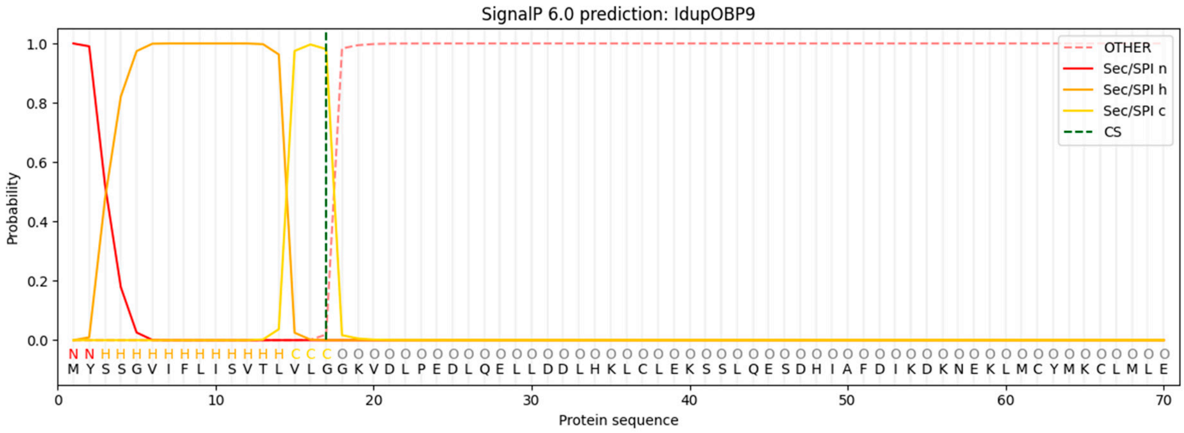

IdupOBP10

Prediction: Signal Peptide (Sec/SPI)

Cleavage site between pos. 19 and 20. Probability 0.977633

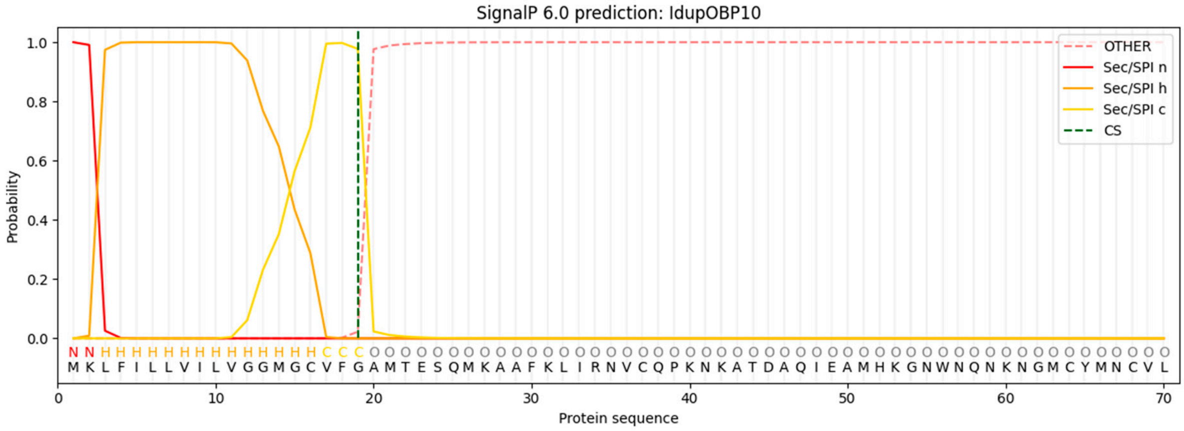

IdupOBP13

Prediction: Signal Peptide (Sec/SPI)

Cleavage site between pos. 18 and 19. Probability 0.975621

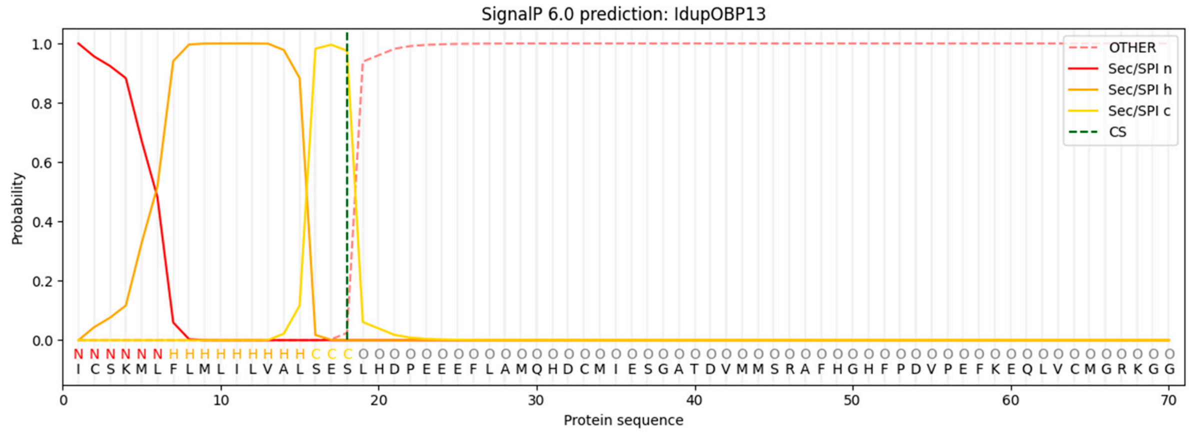

IdupOBP15

Prediction: Signal Peptide (Sec/SPI)

Cleavage site between pos. 16 and 17. Probability 0.967261

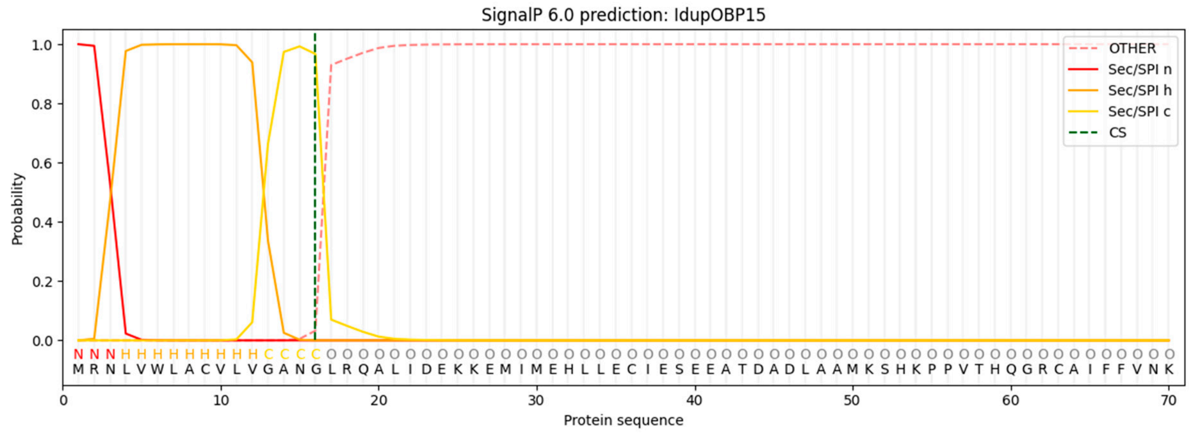

IdupOBP16

Prediction: Signal Peptide (Sec/SPI)

Cleavage site between pos. 18 and 19. Probability 0.970787

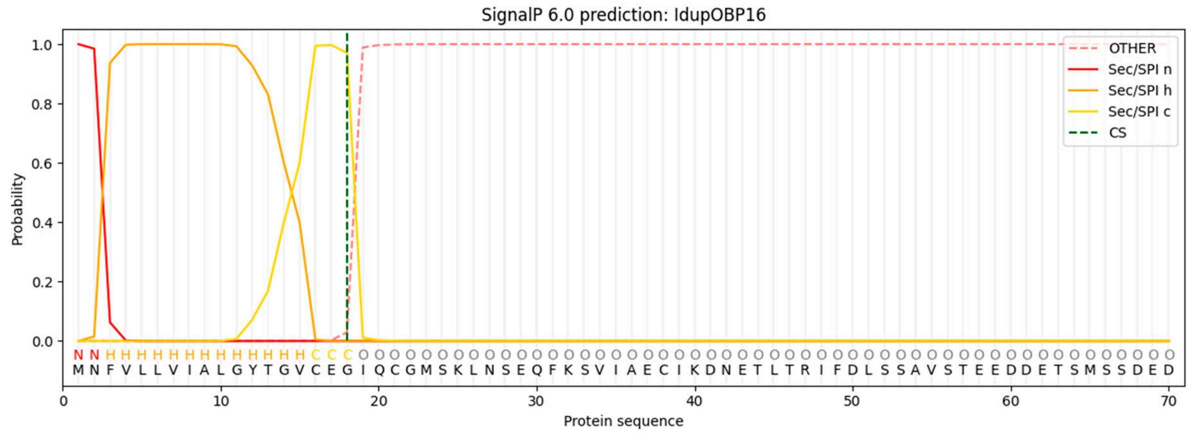

IdupOBP18

Prediction: Signal Peptide (Sec/SPI)

Cleavage site between pos. 26 and 27. Probability 0.971269

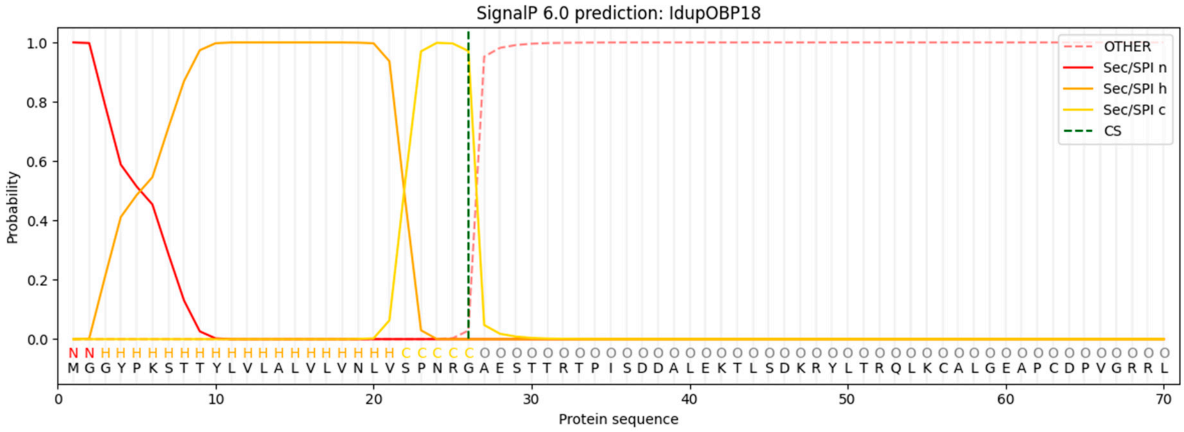

IdupOBP20

Prediction: Signal Peptide (Sec/SPI)

Cleavage site between pos. 22 and 23. Probability 0.977103

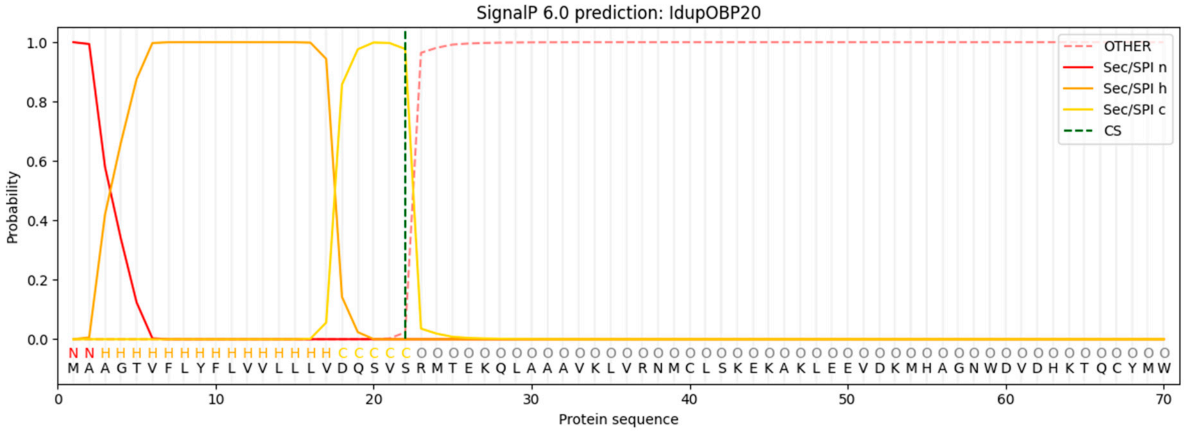

IdupOBP22

Prediction: Signal Peptide (Sec/SPI)

Cleavage site between pos. 18 and 19. Probability 0.982748

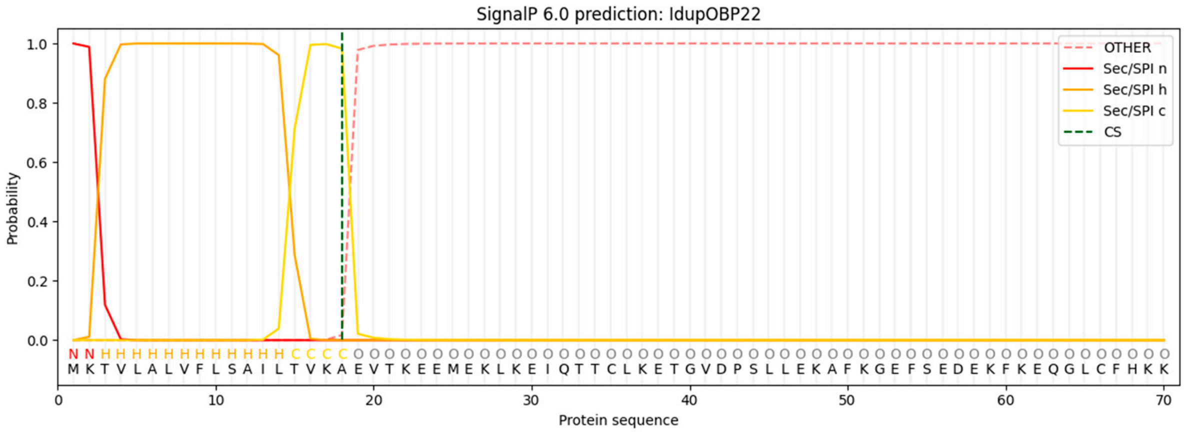

IdupOBP23

Prediction: Signal Peptide (Sec/SPI)

Cleavage site between pos. 24 and 25. Probability 0.976328

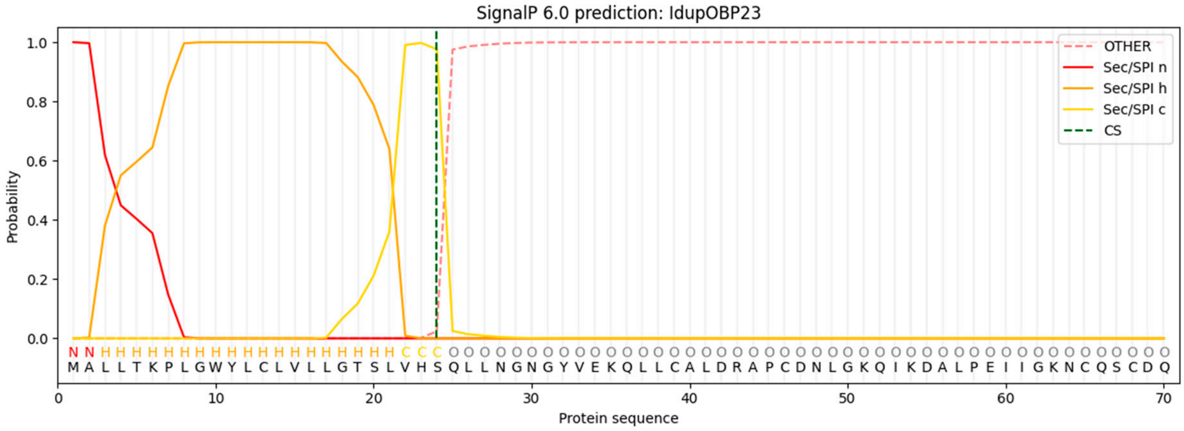

IdupOBP25

Prediction: Signal Peptide (Sec/SPI)

Cleavage site between pos. 20 and 21. Probability 0.786054

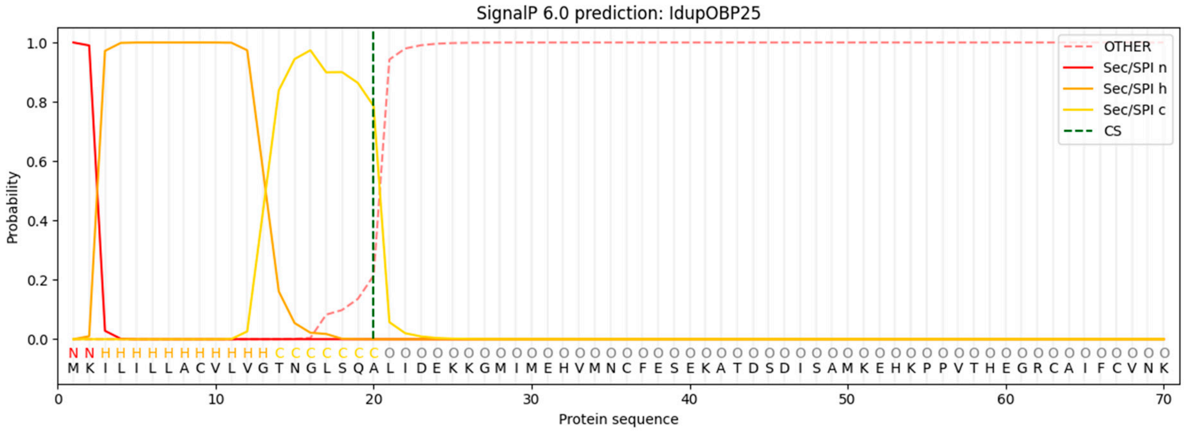

IdupOBP26

Prediction: Signal Peptide (Sec/SPI)

Cleavage site between pos. 17 and 18. Probability 0.979088

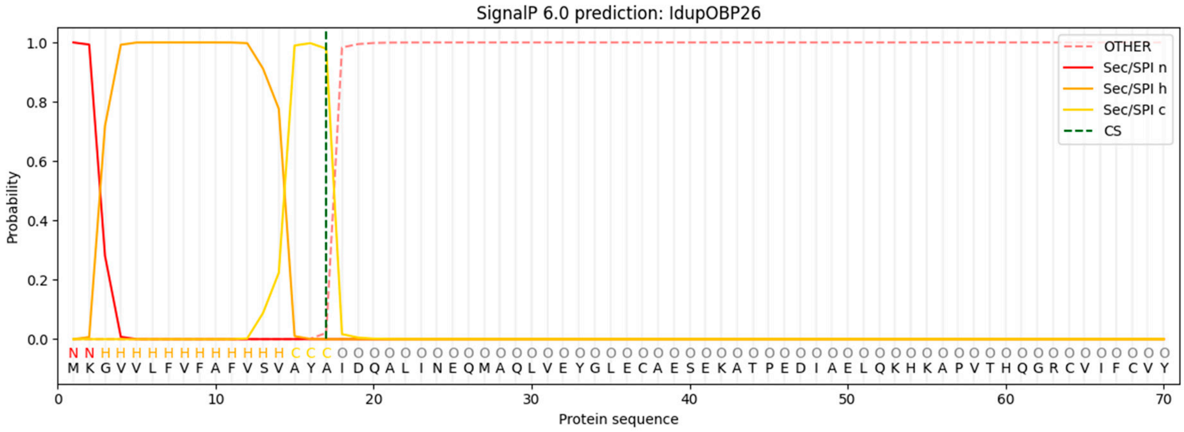

IdupOBP27

Prediction: Signal Peptide (Sec/SPI)

Cleavage site between pos. 16 and 17. Probability 0.981699

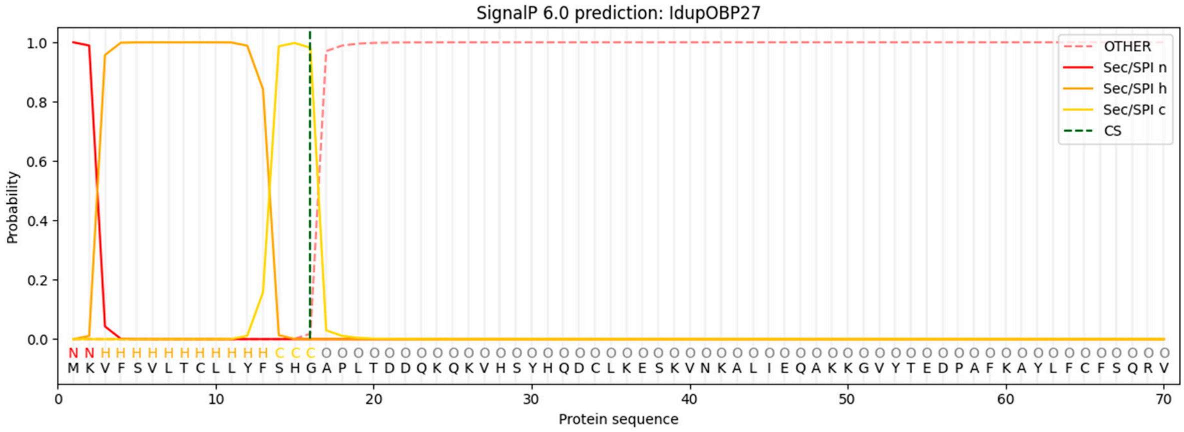

Supplementary information S4. Conserved cysteine residues identified in IdupOBPs.

The alignment was created using MAFFT v.7 [110] under the E-INS-i iterative refinement method. Conserved cysteine residues are colored in red.

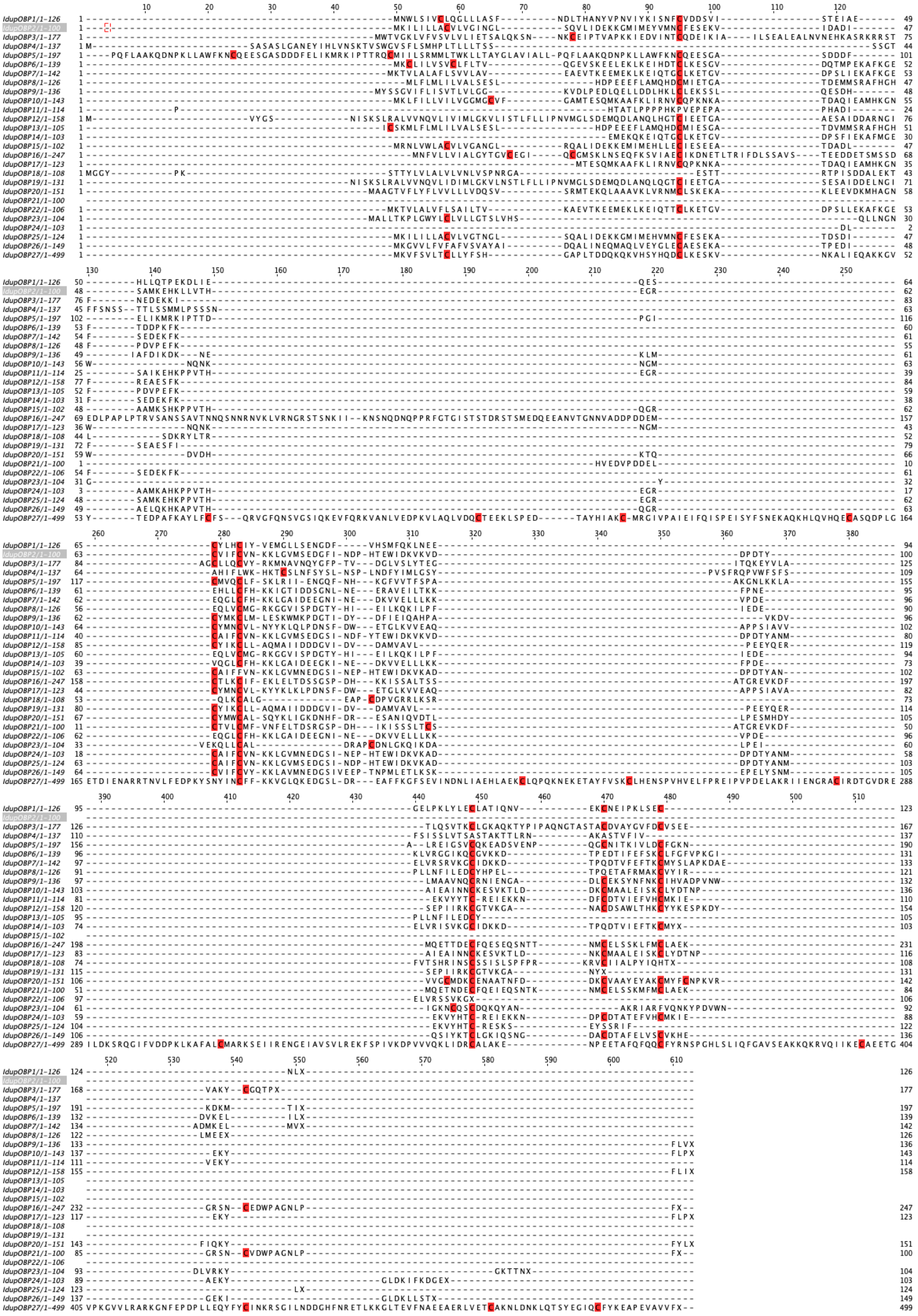

Supplementary Information S5. Conserved cysteine residues in IdupCSPs

The alignment was created using MAFFT v.7 [3] under the E-INS-i iterative refinement method. Cysteine residues are colored in red.

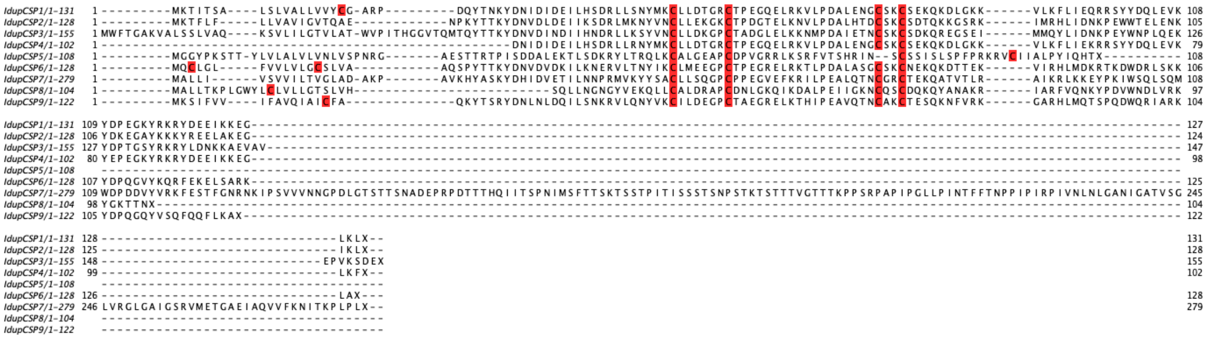

Cited Supplementary Information References from the Manuscript

101. Tsirigos KD, Peters C, Shu N, Käll L, Elofsson A. The TOPCONS web server for consensus prediction of membrane protein topology and signal peptides. Nucleic Acids Res. 2015;43:W401–7.

115. Teufel F, Almagro Armenteros JJ, Johansen AR, Gíslason MH, Pihl SI, Tsirigos KD, et al. SignalP 6.0 predicts all five types of signal peptides using protein language models. Nat. Biotechnol. Springer US; 2022;40.

110. Katoh K, Rozewicki J, Yamada KD. MAFFT online service: multiple sequence alignment, interactive sequence choice and visualization. Brief. Bioinform. [Internet]. 2017;1–7. Available from: https://doi.org/10.1093/bib/bbx108
